# Supplementary material for: Mesoscale, long-time mixing of chromosomes and its connection to polymer dynamics
Source: PLoS Comput Biol. 2023 May 25;19(5):e1011142. doi: 10.1371/journal.pcbi.1011142 (PMC10246856; doi:10.1371/journal.pcbi.1011142)
Supplement: S1 Text — (PDF) [file pcbi.1011142.s001.pdf]

S1 Text: Block copolymer model for *Drosophila* genome: Incorporating chromosome size, euchromatin and heterochromatin domains, and the interaction of lamin with LAD and non-LAD regions

In this model, the genome of *Drosophila* is represented by different sized bead-spring polymers, with each bead corresponding to 5 kbps of DNA. These polymers represent the four chromosomes of *Drosophila*: Chr2, Chr3, Chr4, and ChrX. The beads on the chromosome chains are divided into two types: euchromatic and heterochromatic, based on their attractive strength. The attraction between euchromatic beads is $\epsilon_{\mathrm{EE}} = 0.25\,\mathrm{k_B T}$ and between heterochromatic beads is $\epsilon_{\mathrm{HH}} = 0.5\,\mathrm{k_B T}$, with a distance cutoff of $r_c = 2.5\sigma$. Euchromatic and heterochromatic beads only interact through excluded volume interactions. Therefore, the Lennard-Jones potential between these beads is truncated at the distance where the attractive and repulsive forces are equal, which occurs at a distance of $r_c = 2^{1/6}\sigma$ and an energy of $\epsilon_{EH} = 1\,\mathrm{k_B T}$. Beads of chromosome chains are confined within a sphere with a radius of R_c. The interaction between these beads and the surface of the sphere is repulsive. The nuclear lamina is modeled using additional beads localized to the confinement surface. These beads, called lamin beads, represent the thin laminar shell and are considered as static for simplicity.

In addition to euchromatin and heterochromatin, chromosome chains are divided into two types of beads: LADs (lamin-associated domains) and non-LADs, based on their interactions with lamin beads. The distribution of LADs and non-LADs along the chromosome chain is taken from experimental data [1]. The attraction between LAD and lamin beads is modeled using the Lennard-Jones potential, with a strength of $\epsilon_{lp} = 0.5\,\mathrm{k_B T}$ and a cutoff distance of $r_c = 2.5\sigma$. Non-LAD beads do not have attractive interactions with lamin beads, only excluded volume interactions with a cutoff distance of $r_c = 2^{1/6}\sigma$ and an energy of $\epsilon_{np} = 1\,\mathrm{k_B T}$.

A key feature of the chromatin-lamina interaction in our model is the formation of dynamic bonds (modeled by a harmonic potential) between LAD beads and lamin beads when they are within a certain distance ($r_{\mathrm{bond}} \leq 2.5\sigma$). The spring constant for these bonds is $K = 10\,\mathrm{k_B T}/\sigma^2$. The equilibrium distance for these bonding interactions is $r_0 = \sigma$, meaning that once a bond is formed, it remains stable at that distance. Dynamics bonds can break when the distance between the LAD beads and lamin beads exceeds a certain cutoff ($r_{\mathrm{break}} = 2.5\sigma$). The potential energy of bonds forming and breaking is calculated using the Monte Carlo method in the LAMMPS software [2].

References

[1] Ho JWK, Jung YL, Liu T, Alver BH, Lee S, Ikegami K, et al. Comparative analysis of metazoan chromatin organization. Nature. 2014;512(7515):449–452. doi:10.1038/nature13415.

[2] Plimpton S. Fast Parallel Algorithms for Short-Range Molecular Dynamics. Journal of Computational Physics. 1995;117(1):1 – 19.
