# Supplementary material for: Mesoscale, long-time mixing of chromosomes and its connection to polymer dynamics
Source: PLoS Comput Biol. 2023 May 25;19(5):e1011142. doi: 10.1371/journal.pcbi.1011142 (PMC10246856; doi:10.1371/journal.pcbi.1011142)
Supplement: S2 Text — This text describes the definition and formula of the chromosome mixing index. (PDF) [file pcbi.1011142.s002.pdf]

S2 Text: Chromosome mixing index

We define the chromosome mixing index as the ratio between the total number of interchromosomal contacts and total number of intrachromosomal contacts. This is easily calculated from the experimental Hi-C contact map. The maximum value of the chromosome mixing index is theoretically given by

$$\text{Chromosome mixing index}(\alpha) = \frac{\text{Total interchromosomal contacts}}{\text{Total intrachromosomal contacts}} \tag{1}$$

$$= \frac{(N \times N) - (n \times M \times M)}{(n \times M \times M)}, \tag{2}$$

where N is the total size of the genome, M is the length of the individual chromosome, and n is the total number of chromosomes in the genome.

$$\text{Since } N = n \times M, \text{this gives } \alpha_{max} = n - 1. \tag{3}$$

In the ideal limit (where there are no interactions) a given chromosome can mix with any of the $n-1$ chromosomes. In the calculation of the chromosome mixing index, the number of intrachromosomal contacts enters into the denominator. Therefore, if total intrachromosomal contacts are zero, as in the case of rigid rods, the mixing index will not be applicable. The chromosome mixing index is most appropriate for long polymers whose persistence length is significantly smaller than their total length ($l_p << L$) so that intrachromosomal contacts within a single polymer will be formed; the confined environment of the nucleus also increases this tendency. This is because in such a scenario, the chromosomes are able to mix effectively without diffusing too much in the larger space.
