# Supplementary material for: Mesoscale, long-time mixing of chromosomes and its connection to polymer dynamics
Source: PLoS Comput Biol. 2023 May 25;19(5):e1011142. doi: 10.1371/journal.pcbi.1011142 (PMC10246856; doi:10.1371/journal.pcbi.1011142)
Supplement: S3 Text — (PDF) [file pcbi.1011142.s003.pdf]

S3 Text: Calculating the contact map, contact probability, and chromosome mixing index from simulation data

In every $10^3\tau$ time steps (corresponding to one frame, with a total of 1000 frames), the simulation uses the Langevin equations of motion to generate the position of each bead in Cartesian coordinates. From these coordinates, we calculate the distance (r_{ij}) between any two beads i and j. The first step in generating a contact map from the simulation data is to define the contact criteria based on the distance (r_{ij}) between any two beads i and j. We choose to do this by saying that when the distance between any two beads i and j is less than 1.5 times their minimal distance due to hard sphere repulsion (σ), they are considered to be in contact ($r_{ij} < 1.5\sigma$). By calculating which pairs of beads are in contact, we generate a contact matrix with $N \times N$ elements, which includes all the contact information of the N beads in our system. Since we do not need the details of every contact of every bead for our analysis, we coarse-grain the matrix into blocks of 500 kbps resolution, which reduces the matrix size by a factor of 100. We use the block average method to coarse-grain the contact matrix [1]. In this method, at the mth level of coarse-graining, an $N \times N$ matrix is divided into blocks of size $m \times m$ each. Then, a new contact matrix of size $N_m \times N_m$ where $N_m = N/m$ is constructed in such a way that the value of each element represents the arithmetic mean of the elements in each block. By setting $m = 100$, we coarse-grain the original contact matrix into a 352×352 contact matrix. Because one contact matrix is generated per frame, 1000 frames result in 1000 contact matrices. We denote by C^t_{ij} the contact matrix of the tth frame; then, a mean contact matrix C_{ij} is calculated by averaging the contact matrices over the last 500 frames. We represent the resulting contact map (CM) as a heat map of the mean contact matrix ($\text{CM} = \log_2[C_{ij}]$) in order to visualize the contact matrix.

We use the contact matrix (C_{ij}) to calculate the contact probability ($P(s =\mid i - j \mid)$) between pairs of beads (i, j). We use the full contact matrix (C_{ij}) to cut out four matrices (IC_μ) along the diagonal, each of size $N_m/4$, representing the contact matrix of the μth chromosome. We write $IC = (1/4)\sum_\mu IC_\mu$ which represents the mean intra-chromosome contact matrix of all four chromosomes. The contact probability ($P_\mu(s)$) within each chain is calculated by using the following formula

$$P_\mu(s) = \frac{\sum \text{diag}(IC, s)}{(N_m/4) - s}, \tag{1}$$

which is then averaged over all the chains given by

$$P_c(s) = \frac{1}{4}\sum_\mu P_\mu(s) \tag{2}$$

where $\text{diag}(IC, s)$ are the elements of the sth diagonal of matrix IC. Note that $s = 0$ represents the main diagonal and $s > 0$ is above the main diagonal.

The chromosome mixing index (α) is calculated from the simulation as the ratio between the sum of the number of contacts between chains (**not** including the two nearest-neighbor beads to which each bead is bonded) and the sum of intra-chain contacts. The chromosome mixing index (α) for the tth time frame is calculated by

$$\alpha(t) = \frac{\sum C_{ij}(t) - \sum\sum_\mu IC^\mu(t)}{\sum\sum_\mu IC^\mu(t)} \tag{3}$$

where $\sum C_{ij}$ is the sum of all elements in matrix C_{ij} and t is the time. Here, C_{ij} is the averaged contact matrix of all the chromosomes and IC^μ is the intra-chromosome contact matrix of μth chromosome, as defined above.

References

[1] Shi G, Thirumalai D. From Hi-C contact map to three-dimensional organization of interphase human chromosomes. Physical Review X. 2021;11(1):011051.
