# Supplementary material for: Mesoscale, long-time mixing of chromosomes and its connection to polymer dynamics
Source: PLoS Comput Biol. 2023 May 25;19(5):e1011142. doi: 10.1371/journal.pcbi.1011142 (PMC10246856; doi:10.1371/journal.pcbi.1011142)
Supplement: S4 Text — In this text, for an unconfined single chain, we discuss how the attraction of the beads characterized by the parameter ϵ affects the radius of gyration (Rg), mean-square displacement (MSD) as a function of time, and the contact probability scaling exponent (γ). (PDF) [file pcbi.1011142.s004.pdf]

S4 text: Unconfined single chain as a function of self-attraction strength

Here, we discuss how the attraction of the beads characterized by the parameter ϵ affects the radius of gyration (R_g), mean-square displacement (MSD) as a function of time, and the contact probability scaling exponent (γ) of an unconfined single chain. In S5 Fig(a), we have shown 3D snapshots of our simulations of unconfined, single chains of $M = 8810$ beads by varying the attraction strength for the cases of persistence lengths of both 1 and 5 beads persistence. To better understand how the different chain segments mix in 3D, we divide the chain into 10 different colors along its length, from blue at one end to red at the other. In S5 Fig(b), we plot the radius of gyration of a chain with a persistence length of 1 bead as a function of the interaction strength. For $\epsilon = 0$, the radius of gyration obeys the power law scaling as a function of the chain length, M: $R_g \sim M^\nu$ with $\nu = 1/2$, consistent with the pure random walk of a phantom polymer. For small values of $\epsilon > 0$ to $\epsilon \leq 0.25$ the beads repel each other and R_g increases with ϵ indicative of chain swelling due to exclude volume repulsion. For $\epsilon > 0.25$, R_g decreases with the interaction strength signifying collapse; we observe a sharp drop starting at $\epsilon = 0.25$ and we estimate that at $\epsilon = 0.3$ the chains undergo a collapse transition. This is consistent with an estimate of the theta-point (where the second virial coefficient [1] for the LJ potential vanishes) based on the LJ potential (see S6 Fig). This defines the critical value ϵ_c of the collapse transition.

For larger values of ϵ ($\epsilon \geq 0.5$), the radius of gyration as a function of chain length follows the scaling exponent $\nu = 1/3$ indicates that the polymer is collapsed since it is in a poor solvent. The case of the persistence length l_p of 5 beads is similar. S5 Fig(c), shows that the radius of gyration follows power law scaling $R_g \sim l_p\,(M/l_p)^\nu$ with $\nu = 1/2$ for $\epsilon = 0$ and $\nu = 1/3$ for $\epsilon \geq 0.5$. for the case of the 5 bead persistence length we observed the first collapse at $\epsilon_c = 0.4$ instead of $\epsilon_c = 0.3$ for the case where $l_p = 1$ bead. This means a chain with $l_p = 5$ beads requires is stiffer over a longer distance and requires a larger attraction among the beads in order to collapse.

From these simulations we have calculated the contact probability that follows a scaling law $P(s) = s^{-\gamma}$ and present the results of the scaling exponent γ as a function of ϵ in S5 Fig(d). For a unconfined single chain of persistence of 1 and 5 beads, the contact probability scaling exponent is $\gamma = 1.5$ for $\epsilon = 0$ (random walk chain). This is indeed consistent with the behavior of a completely random, Gaussian chain where the normalization of the probability yields $\gamma = 3/2$. In S5 Fig(d), we plot the contact probability scaling exponent $\gamma > 1$ for an open chain ($\epsilon < \epsilon_c$) and $\gamma \leq 1$ for a collapsed chain ($\epsilon \geq \epsilon_c$). For the collapsed chain the values of $0 < \gamma < 1$ signify intermediate level mixing while $\gamma = 1$ shows no mixing of different parts of chain that are far apart.

In addition, we also calculated the mean-square displacement (MSD) of a bead (averaged over all the beads of the chain) by varying the LJ attraction strength (see S5 Fig(e) and S5 Fig(f)). For $\epsilon = 0$ and 0.25 when chain is open, MSD $\sim \tau^{1/2}$ and bead moves like a monomer in a Rouse chain [2]. For $\epsilon = 0.5, 0.75, 1$ when chain is strongly collapsed, MSD $\sim \tau^{1/4}$ and bead moves like a monomer in a reptating chain [3]. Every bead in a collapsed chain must cross the crowded environment of other beads in order to move, so the reptation model is more appropriate. We have also shown these results do not change by changing chain's persistence length from $l_p = 1$ bead to $l_p = 5$ beads (see S5 Fig(e) and S5 Fig(f)).

References

[1] Hirschefelder JO, Curtiss CF, Bird RB. Molecular theory of gases and liquids. New York. 1954;.

[2] De Gennes PG. Quasi-elastic scattering of neutrons by dilute polymer solutions: I. Free-draining limit. Physics Physique Fizika. 1967;3(1):37.

[3] De Gennes PG. Reptation of a polymer chain in the presence of fixed obstacles. The journal of chemical physics. 1971;55(2):572–579.
