# Supplementary figures and images for: Mesoscale, long-time mixing of chromosomes and its connection to polymer dynamics
Source: PLoS Comput Biol. 2023 May 25;19(5):e1011142. doi: 10.1371/journal.pcbi.1011142 (PMC10246856; doi:10.1371/journal.pcbi.1011142)

(a) Initial structure of single chain

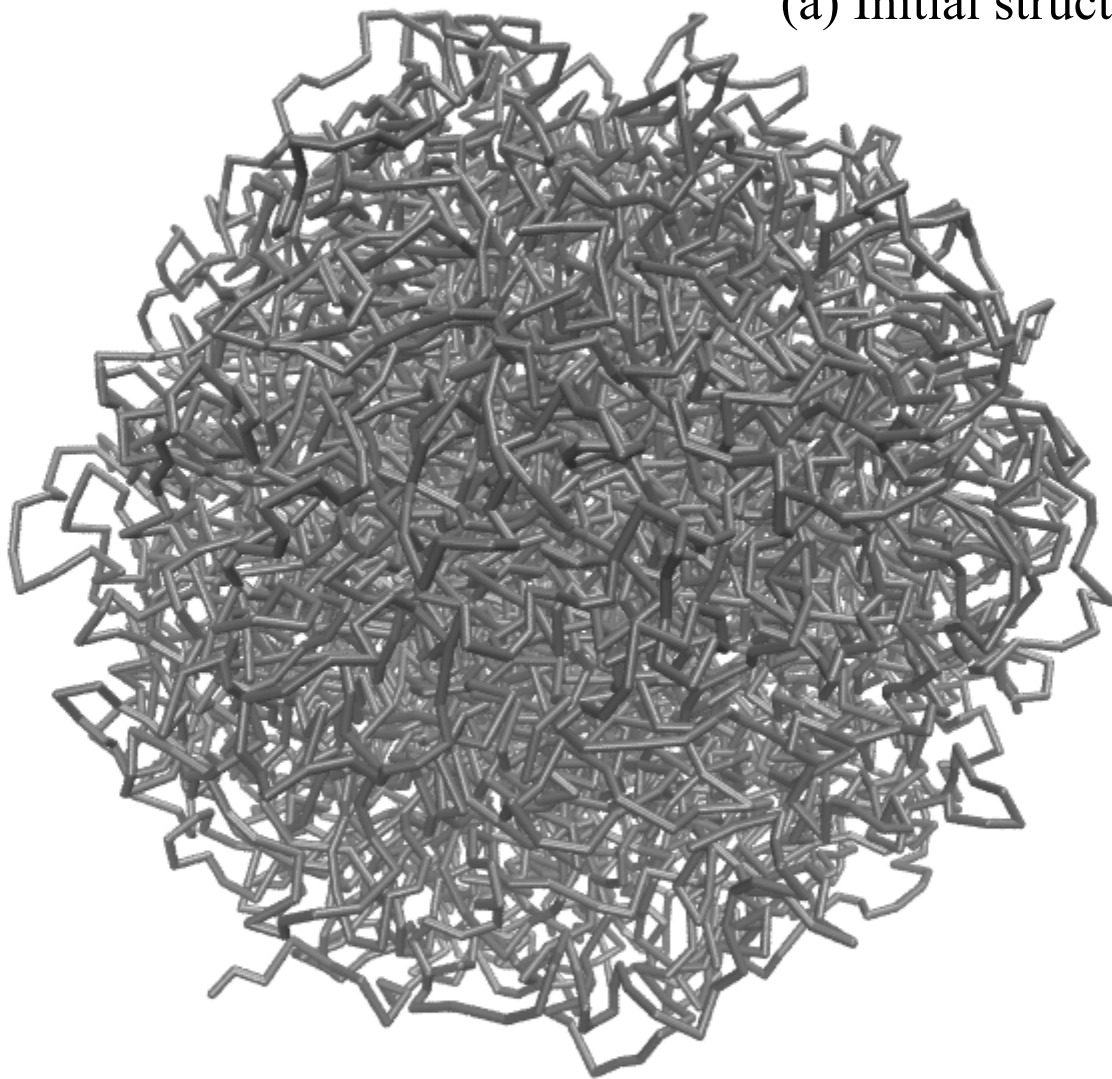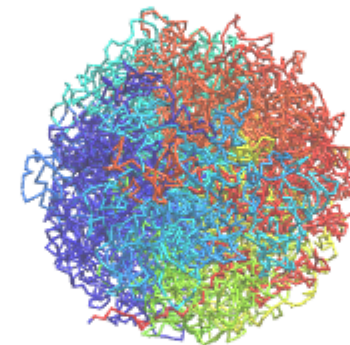

(b) Late time snapshot of self-avoiding chain

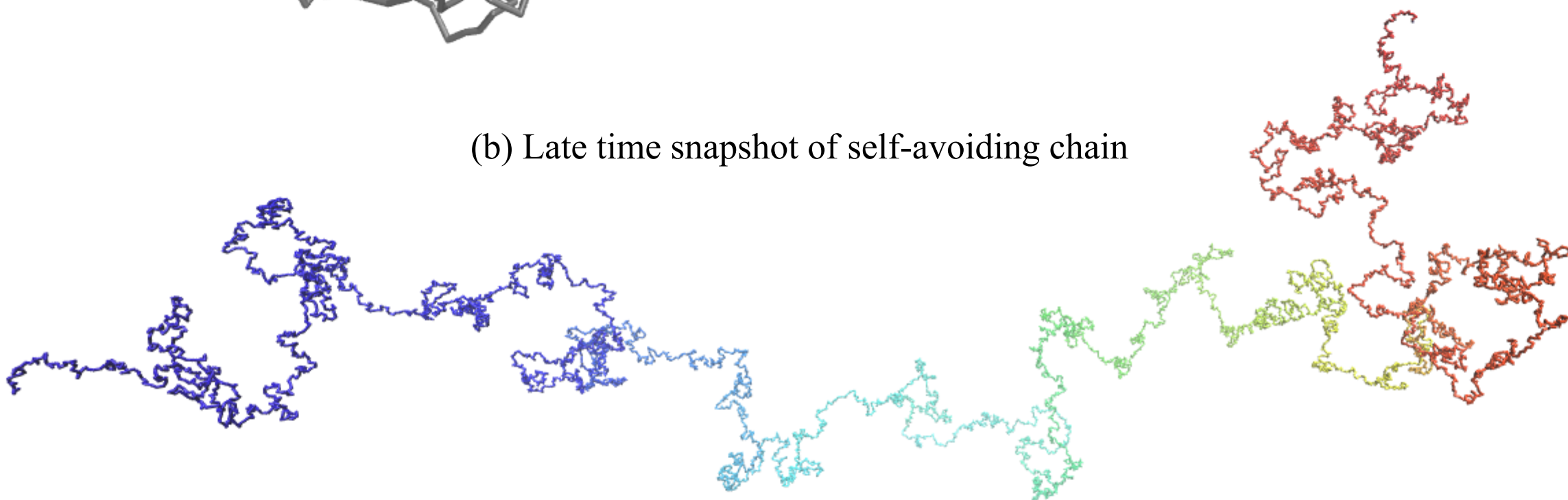

Supplement: S1 Fig — (a) Upper left: The initial structure of a single chain of 8810 beads was generated using the moltemplate software from python code interpolate_coords.py [85]. This code uses cubic spline interpolation to create a smooth polymer structure within a given size of the cubic box. Upper right: The initial structure was colored along its length using 10 different colors, ranging from blue at one end to red at the other. (b) A late-time snapshot of a self-avoiding chain from simulation. The simulation was conducted with a persistence length of 1 bead and a simple Lennard-Jones potential with a strength of ϵ = 1kBT and a cutoff of rc = 21/6σ. The snapshot shows an open chain structure with separated colors, indicating that the initial structure was unknotted. (PDF) [file pcbi.1011142.s005.pdf]

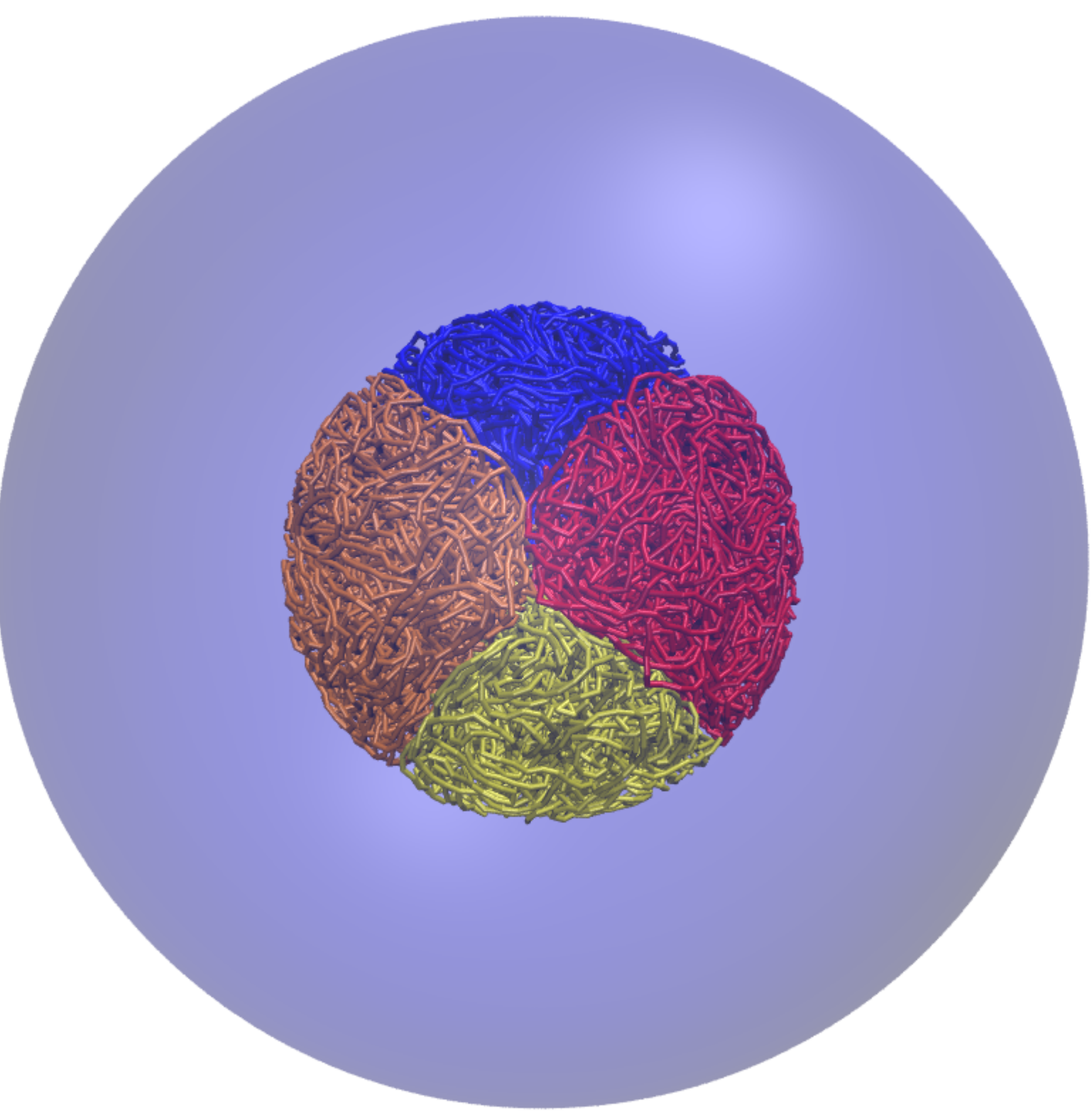

Supplement: S2 Fig — The initial structure consists of four separate chains confined in a small space. By using moltemplate [85], one chain was generated, and then, by using packmol [103], four copies of chains were placed in a cubic box with a size of L = 100σ. The polymer system was then compressed to a small spherical confinement radius of Rc = 20σ (or ϕ = 0.6) using indented walls and Lennard-Jones (LJ) interactions. The force exerted by a spherical indenter on each bead is represented by the equation F(r) = −Kindent(r − Rindent)2, where Kindent is the specified force constant, r is the distance from the bead to the center of the indenter, and Rindent is the radius of the indenter [83]. The intra-chain interactions were attractive, with a strength of ϵintra = 1kBT and a cutoff distance of rc = 2.5σ, while the inter-chain interactions were repulsive, with a strength of ϵinter = 1kBT and a cutoff distance of rc = 21/6σ. A snapshot of the initial structure is shown in a confinement with a volume fraction of ϕ = 0.1, which illustrates that the chains were initially separated and condensed. (PDF) [file pcbi.1011142.s006.pdf]

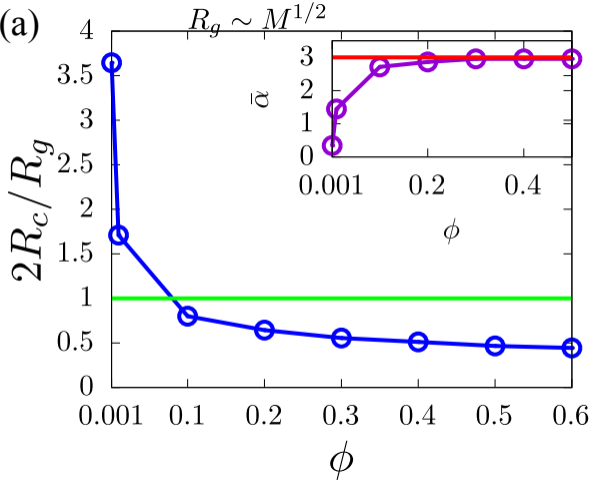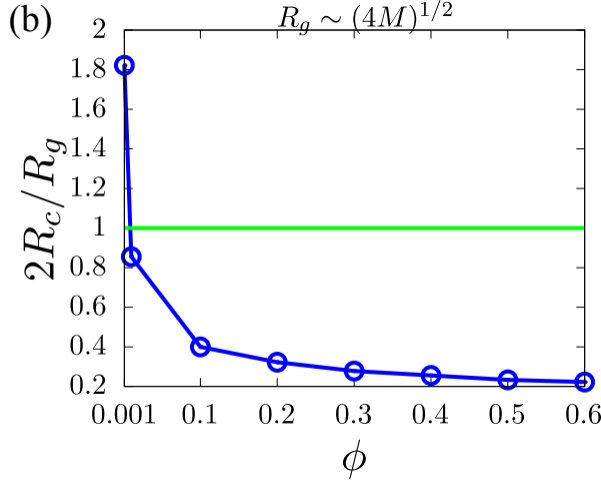

Supplement: S3 Fig — (a) In the phantom chain case (ϵ = 0), the ratio of the diameter of confinement (2Rc) to the radius of gyration of a random-walk chain (Rg) is plotted as a function of the chain volume fraction in the nucleus. S5c Fig shows that the radius of gyration of a random-walk chain of M = 8810 beads is Rg = 90σ when the persistence length is 5 beads. The inset of the figure shows the time-average of the chromosome mixing index (α¯) as a function of the chain volume fraction (ϕ) in the nucleus. From both figures, it is clear that the chromosome mixing index reaches its maximum value when 2Rc/Rg < 1. Thus when Rg is large relative to 2Rc the confinement is effective; in the opposite case, the confinement is so large that the chains hardly mix with their diffusion in the large confinement volume greatly impeding mixing, even for phantom chains. (b) In the case of excluded volume chains (where ϵ ≠ 0), the confinement has an even larger effect. The radius of gyration for 4 chains of M beads each (4M beads in total, equivalent to the Drosophila genome) is taken to be Rg = 180σ. It is noteworthy that for ϕ = 0.01, in the phantom chain case (a) 2Rc/Rg > 1, but in the non-phantom chains case (b) 2Rc/Rg < 1. (PDF) [file pcbi.1011142.s007.pdf]

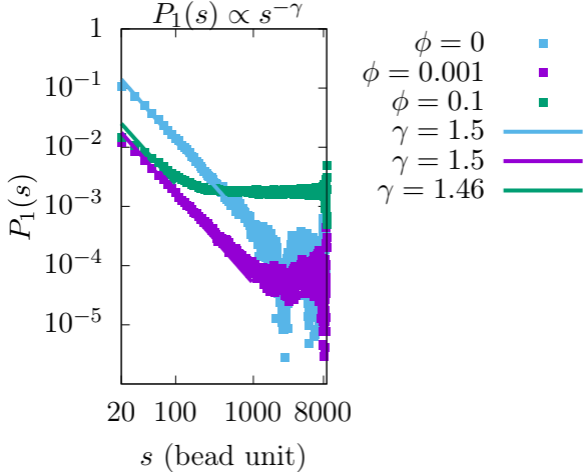

Supplement: S4 Fig — The contact probability of a phantom chain, which is defined as the probability of a chain’s beads coming into contact in the 3D space, is shown as a function of the volume fraction ϕ of the chain. The contact probability follows a power law relation with the contour distance s as P1(s) ∼ s−γ. The exponent γ is plotted for three different volume fractions: ϕ = 0 (unconfined single phantom chain), ϕ = 0.001 (4 chain simulation in relatively large confinement volume), and ϕ = 0.1 (4 chain simulation in relatively small confinement volume). For the unconfined polymer, ϕ = 0, the exponent is γ = 1.5, which is same to the theoretically calculated value of γ = 1.5 [39]. In a relatively large confinement volume, the exponent is γ = 1.5, and in a relatively small confinement volume, the exponent is γ ≈ 1.5 between 20 < s < 200, and saturates to a constant for s > 200 beads, as expected for an equilibrium polymer [87]. Note that these results are based on the contact probability P1(s) calculated for the first chain from a simulation of four phantom chains. (PDF) [file pcbi.1011142.s008.pdf]

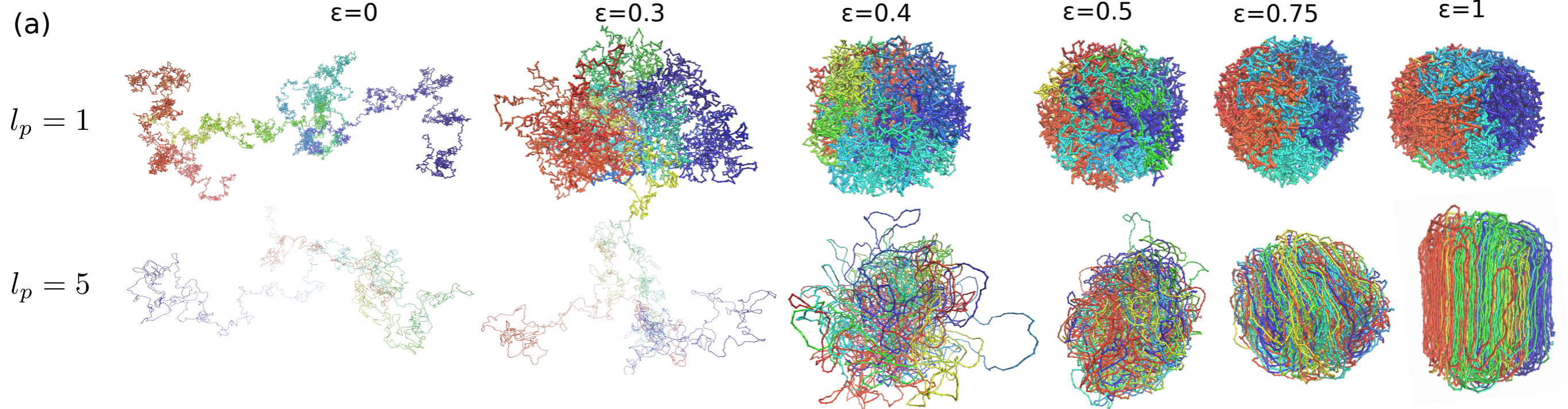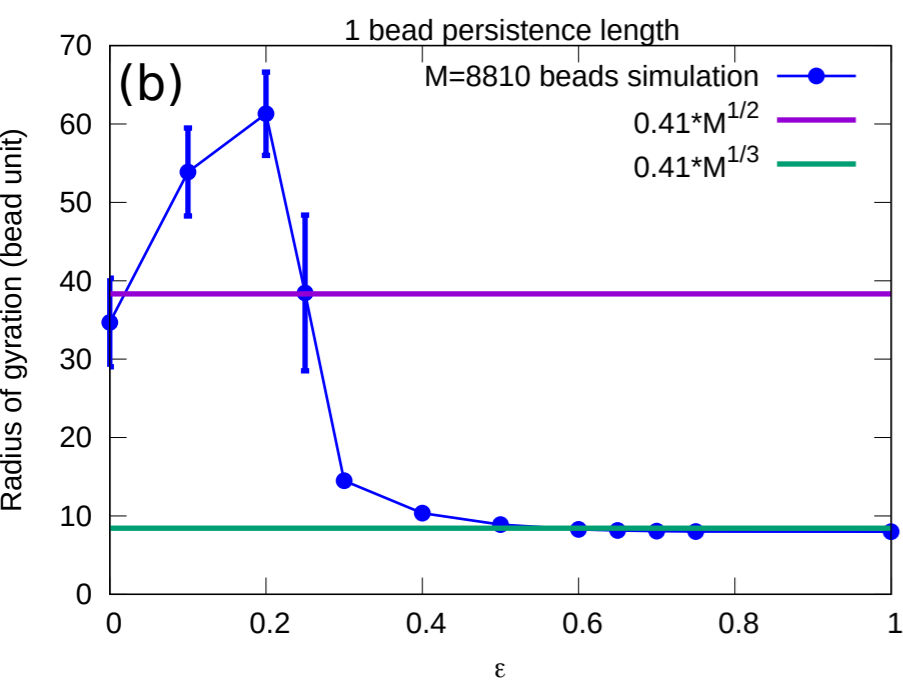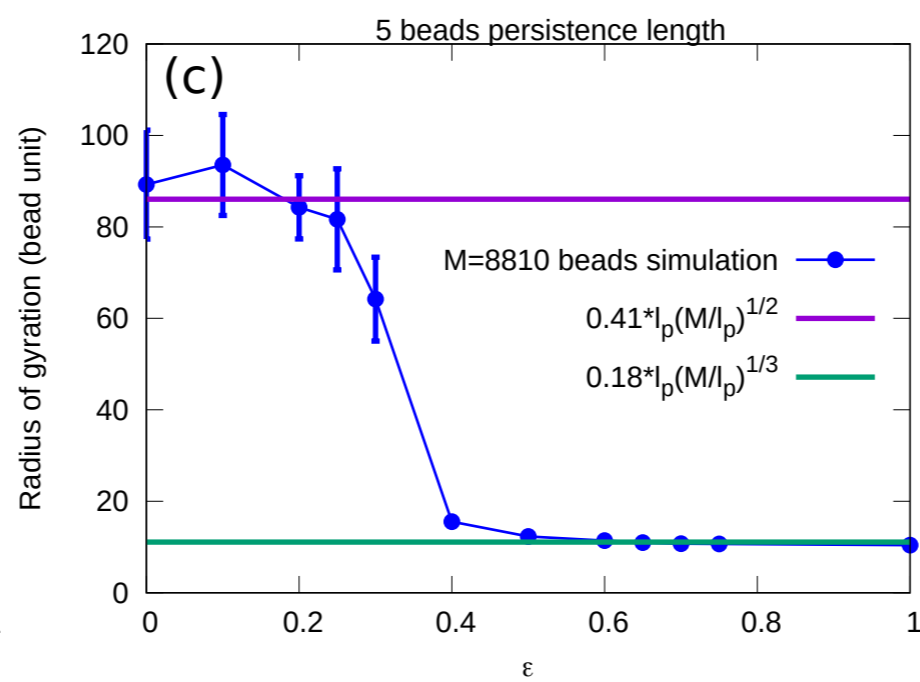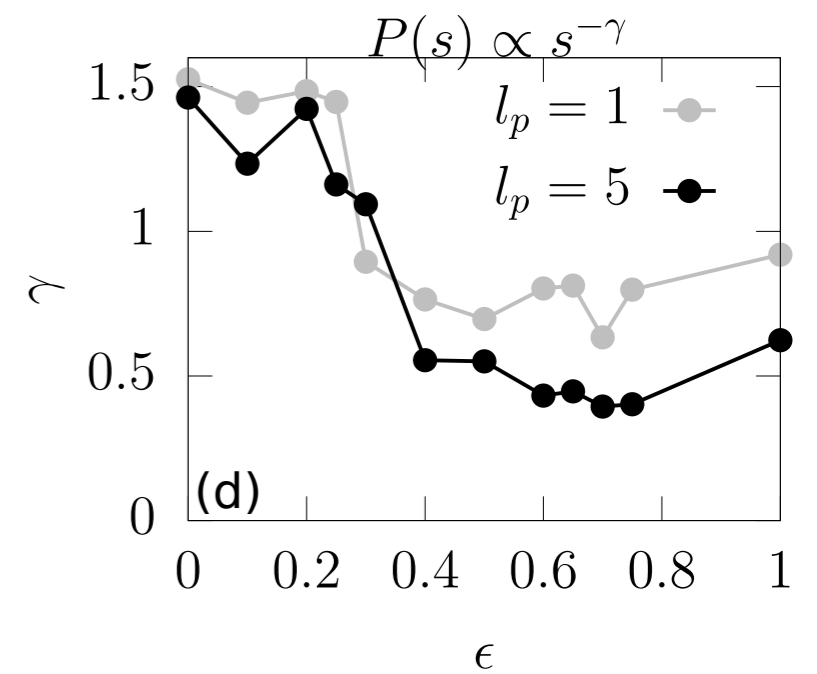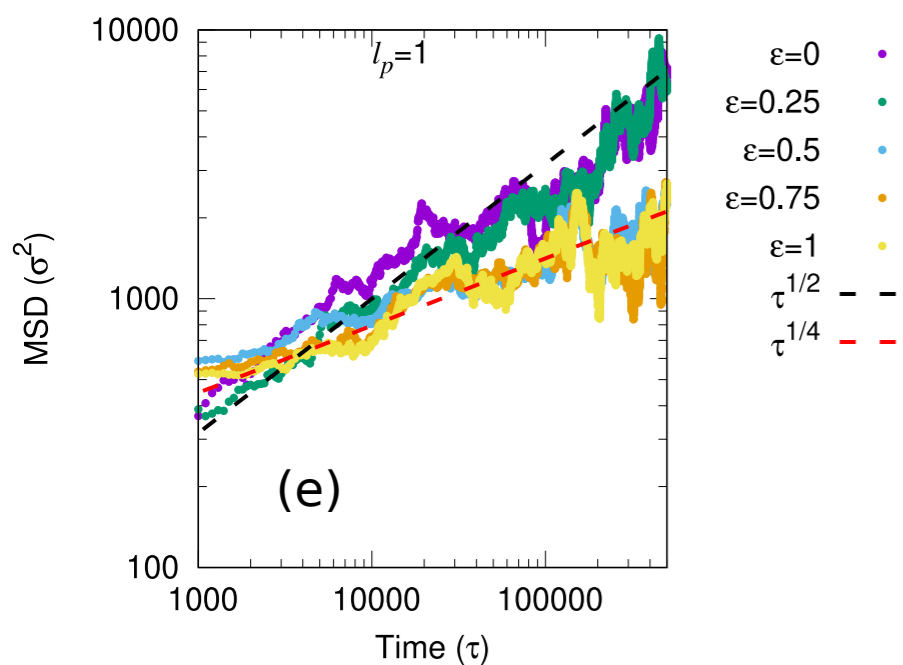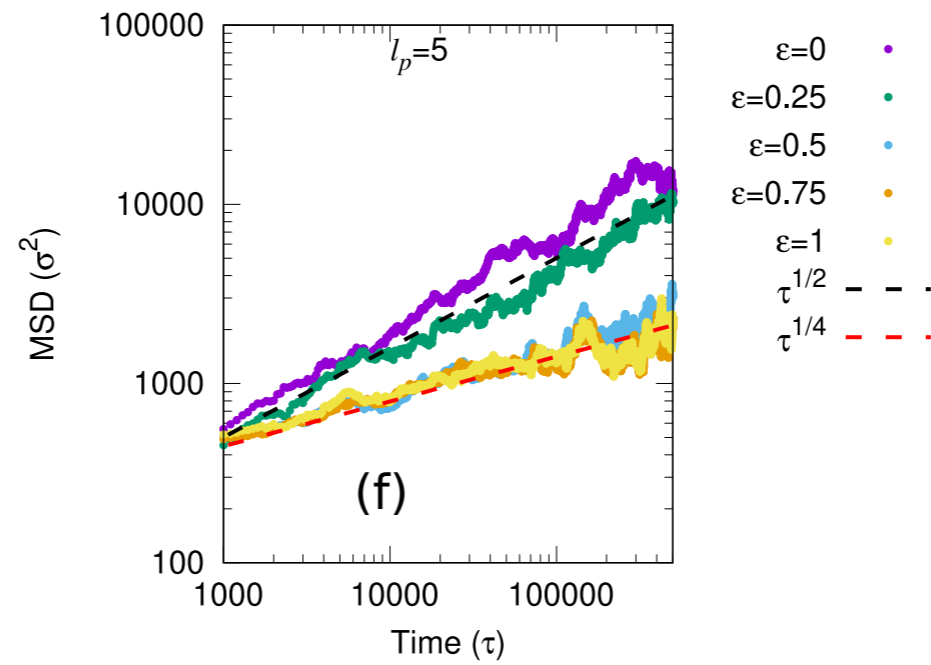

Supplement: S5 Fig — (a) Late time snapshots (t = 106τ time steps) of simulated, unconfined single chains for persistence lengths of lp = 1 and lp = 5 beads as a function of the LJ interaction strength, ϵ. The chain is colored from blue to red along its length to exhibit the mixing of different segments of the chain. The radius of gyration as a function of LJ attraction strength for the persistence length (b) lp = 1 beads and (c) lp = 5 beads. Simulated results are compared with the scaling law of the radius of gyration (Rg ∼ Mν). (d) Contact probability scaling exponent as a function of ϵ for lp = 1 bead (gray color) and lp = 5 bead (black color). Mean-square displacement (MSD) of a bead (averaged over all the beads of the chain) is calculated for the persistence length (e) lp = 1 bead and (f) lp = 5 beads. In both figures (e) and (f), the MSD fit to τ1/2 (black dotted line) for ϵ = 0 and 0.25 and fit to τ1/4 (red dotted line) for ϵ = 0.5, 0.75, 1. (PDF) [file pcbi.1011142.s009.pdf]

# Second virial coefficients for LJ potential

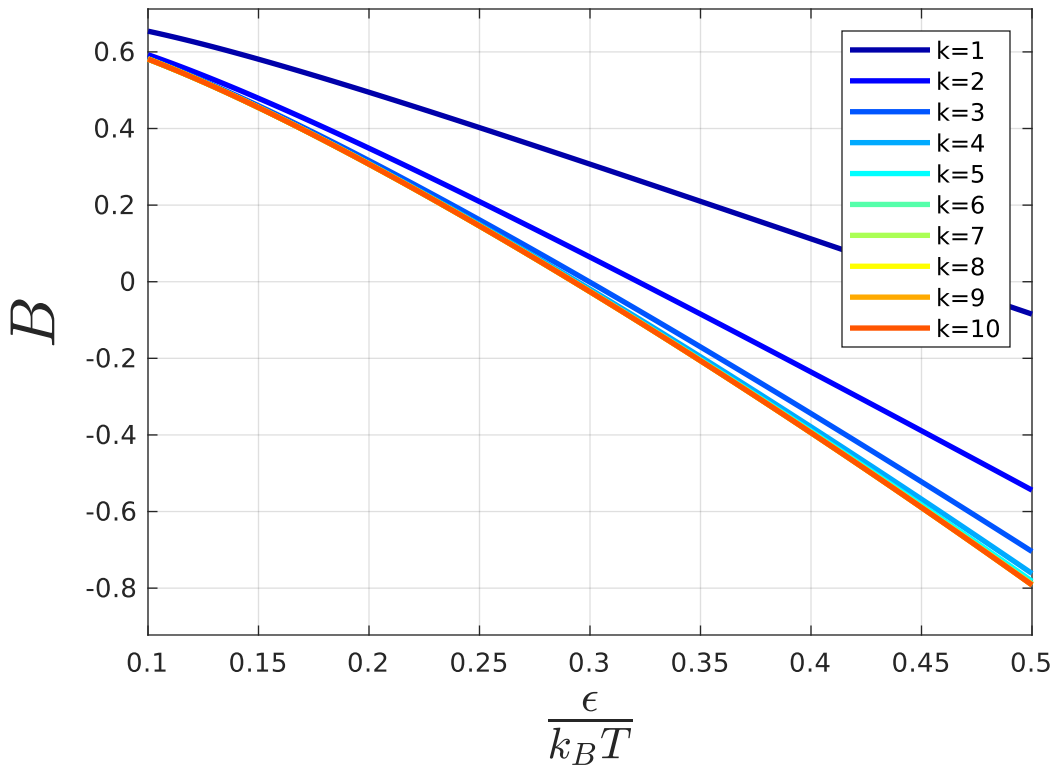

Supplement: S6 Fig — The LJ potential describes both the attraction and repulsion between non-bonded beads. SVC is used to determine the value of ϵ for which repulsive and attractive bead-bead interactions are equal (theta solvent condition). The integral for B(T) cannot be calculated analytically, but an accurate approximation can be derived from the expansion B(T)=-(2π/3)NAσ3limk→∞[∑j=0k2(j+1/2)4j!Γ(2j-14)(ϵkBT)(2j+1)/4] [88]. In the figure, the function B(T) is plotted as a function of ϵ/ kBT for k = 1 to 10. For larger values of k, the graph saturates, and at B = 0, we get ϵθ = 0.2925 kBT. (PDF) [file pcbi.1011142.s010.pdf]

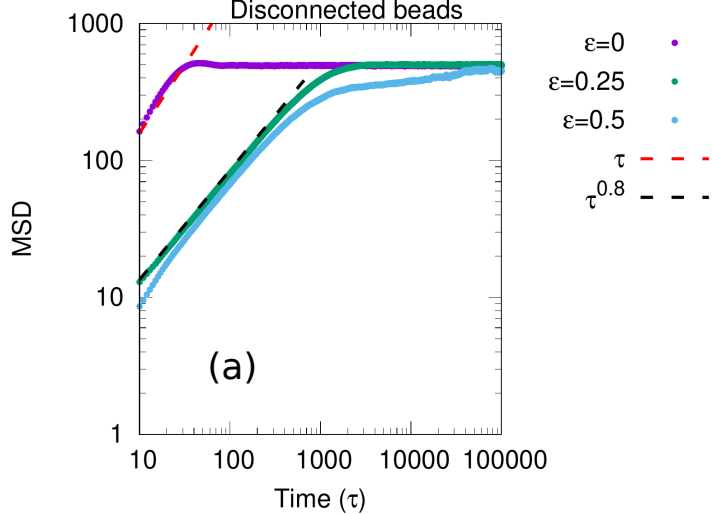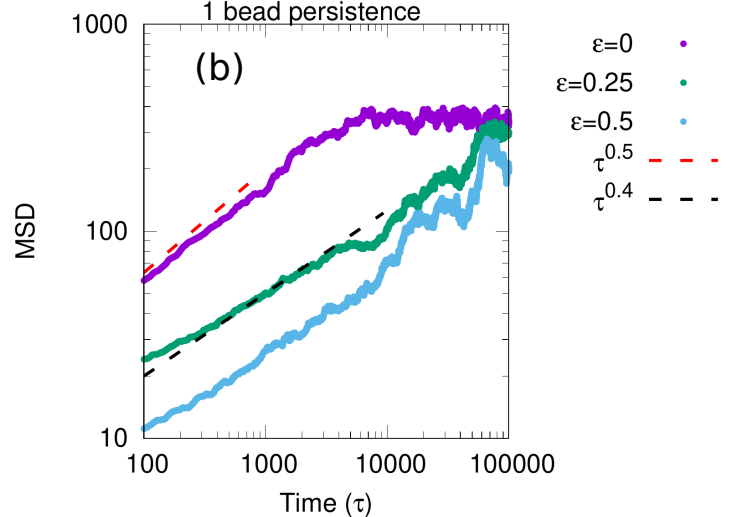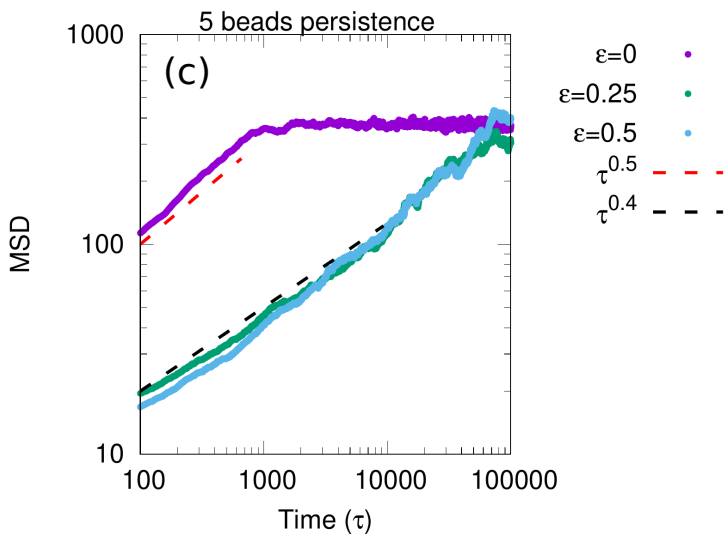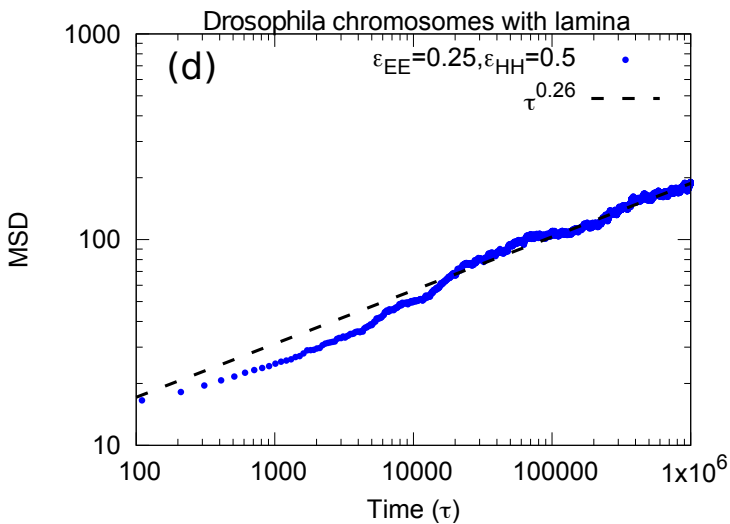

Supplement: S7 Fig — In (a),(b), and (c), MSD are calculated from simulations for phantom chains (ϵ = 0), repulsive chains (ϵ = 0.25), and attractive chains (ϵ = 0.5) for a confinement volume equivalent to a bead volume fraction of ϕ = 0.4. Within this confinement, the MSD of the bead increases with time, and when its value reaches the square of the confinement radius, the MSD saturates at a constant value. (a) When beads are disconnected, for ϵ = 0, the MSD increases linearly with time (MSD ∼ τ) and for ϵ = 0.25 and 0.5, MSD ∼ τ0.8. These results demonstrate that for ϵ = 0, beads behave as independent diffusive particles, and sub-diffusion occurs when we introduce interactions (repulsion or attraction) between them. MSD of chain with persistence lengths of (b) 1 bead and (c) 5 beads, for different interaction strengths, ϵ, are shown. For ϵ = 0, MSD ∼ τ0.5 is the result expected from Rouse chains [15]. For repulsive (ϵ = 0.25) and attractive (ϵ = 0.5) interactions, MSD ∼ τ0.4 [16]. We note that repulsion from the spherical wall speeds up the collapse of the chain compared to the unconfined case in S5f Fig. (d) MSD is calculated from our block copolymer model in the presence of lamina, for a confinement volume equivalent to a bead volume fraction of ϕ = 0.3. This result reveals very slow mixing dynamics, as evidenced by the MSD ∼ τ0.26 scaling law. Remarkably, the exponent of time in the MSD matches that predicted by reptation dynamics [12]. (PDF) [file pcbi.1011142.s011.pdf]

(a)

$\varepsilon=0.25$

$\varepsilon=0.3$

$\varepsilon=0.4$

$\varepsilon=0.5$

$\varepsilon=0.75$

$\varepsilon=1$

$l_p = 1$

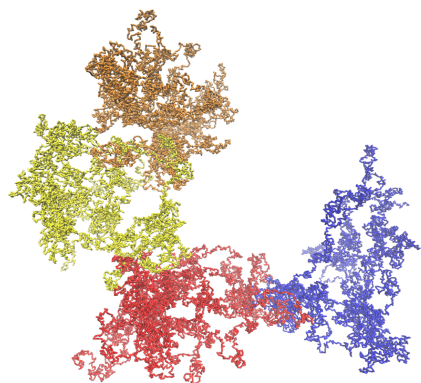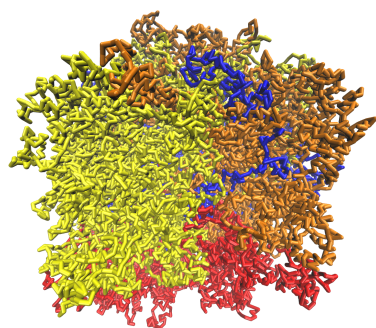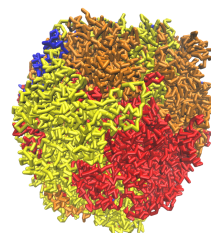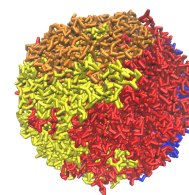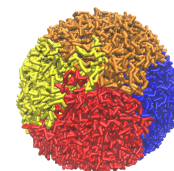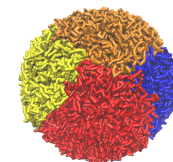

$l_p = 5$

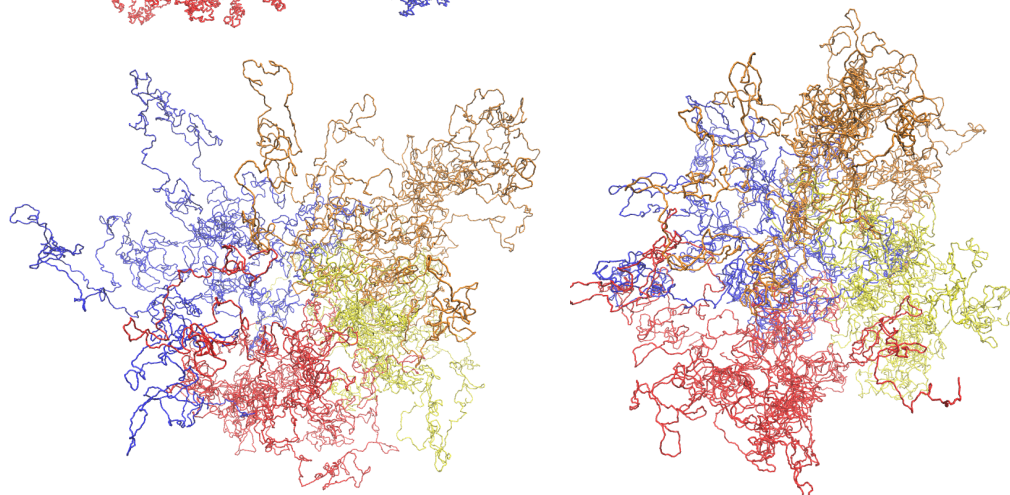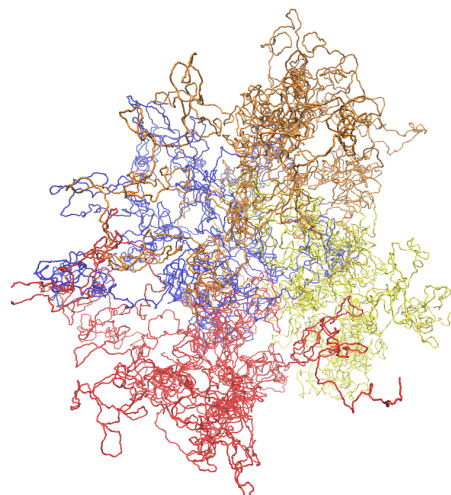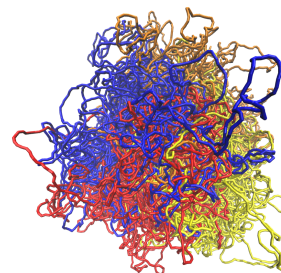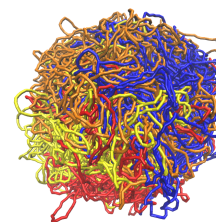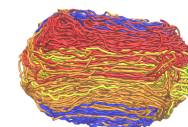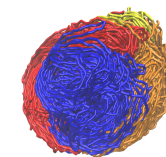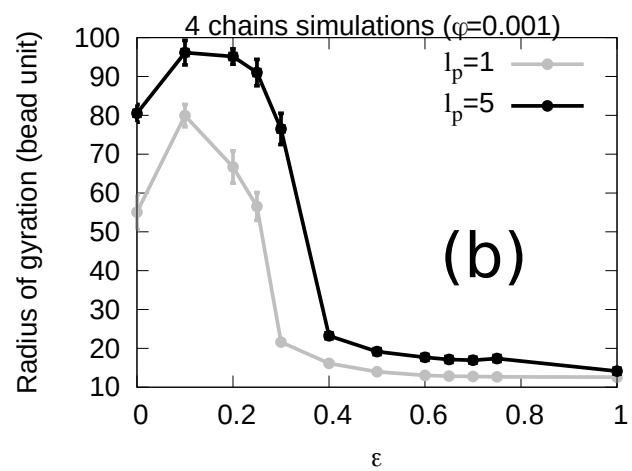

Supplement: S8 Fig — (a) Simulation snapshots show the mixing of four chains mixing as a function of interaction strength, ϵ, for persistence lengths lp = 1 bead and lp = 5 beads. Note that some snapshots were zoomed out because they were too large and would take up too much space if they were shown at their actual size. (b) The radius of gyration as a function of ϵ for lp = 1 bead (gray color) and lp = 5 beads (black color). Note that the attraction strength at which collapse occurs for the one-bead persistence length is ϵc = 0.3, whereas for the 5-beads persistence length, ϵc = 0.4. (PDF) [file pcbi.1011142.s012.pdf]

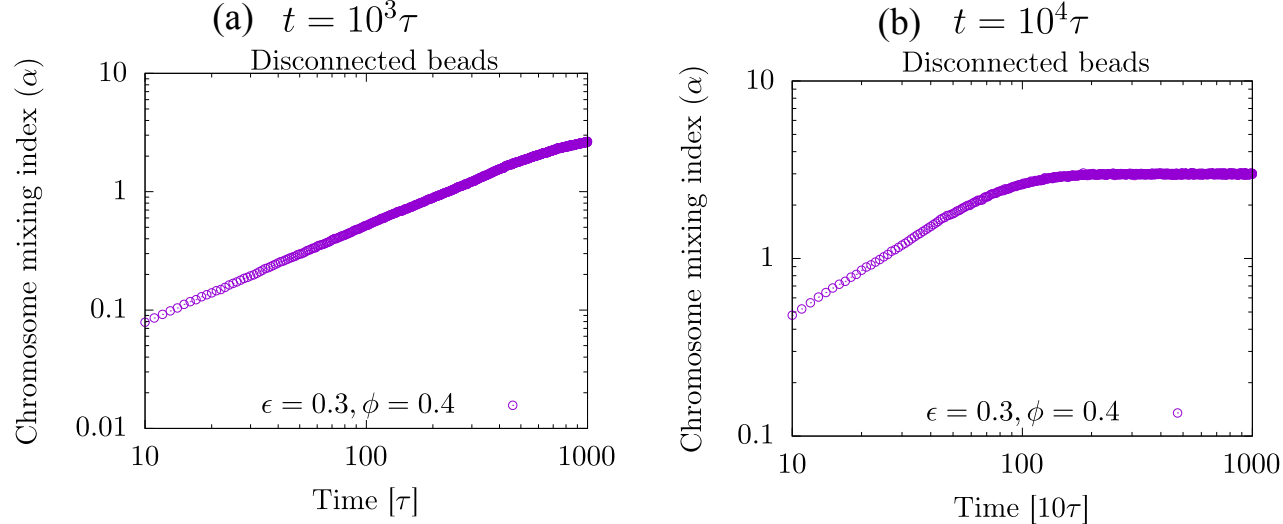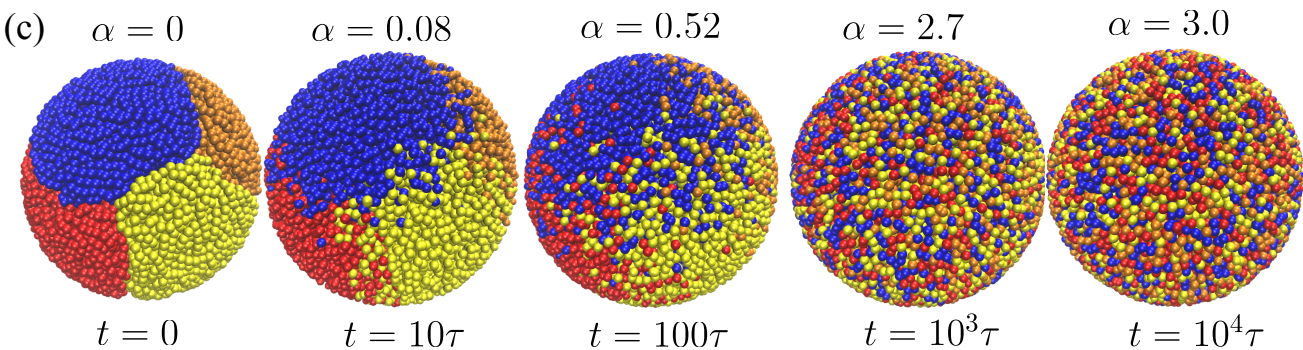

Supplement: S9 Fig — Figure shows the relationship between the chromosome mixing index and time in simulation runs of different durations. (a) When the simulation was run for a shorter period of time (t = 103τ), the chromosome mixing index follows a power law relationship with time. (b) When the simulation was run for a longer period of time (t = 104τ), the chromosome mixing index reaches a constant, maximal value (α = 3). (c) Snapshots and calculated chromosome mixing index values from the simulation of mixing disconnected beads at various times. (PDF) [file pcbi.1011142.s013.pdf]

Chromosome mixing index ( $\alpha$ )

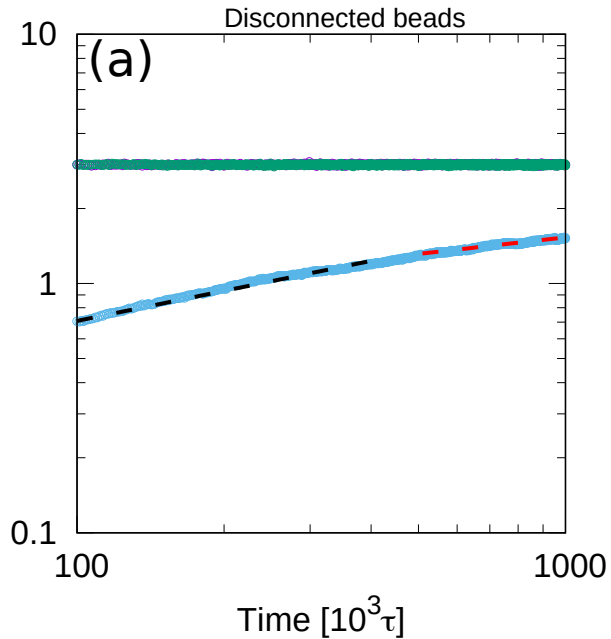

$\epsilon=0$  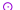  
 $\epsilon=0.25$  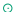  
 $\epsilon=0.5$  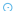  
0.4 - - -  
0.22 - - -

(b)  $\epsilon=0.25$

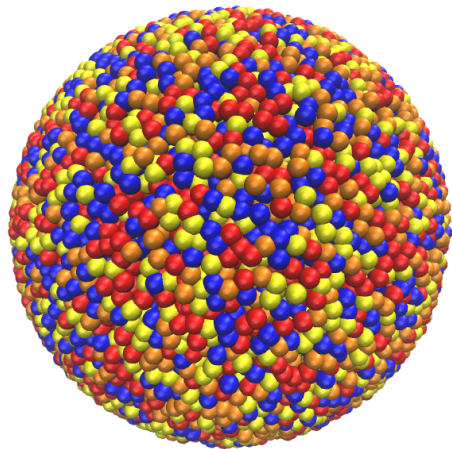

Supplement: S10 Fig — Simulation results are shown for volume fraction ϕ = 0.4. (a) Mixing index α are shown for different ϵ. For ϵ = 0.25, disconnected beads mix quickly and reach the maximum value of the mixing index (α = 3). (b) Late time snapshot (t = 106τ time steps) of simulated disconnected chromosome beads for attraction strength ϵ = 0.25. (PDF) [file pcbi.1011142.s014.pdf]

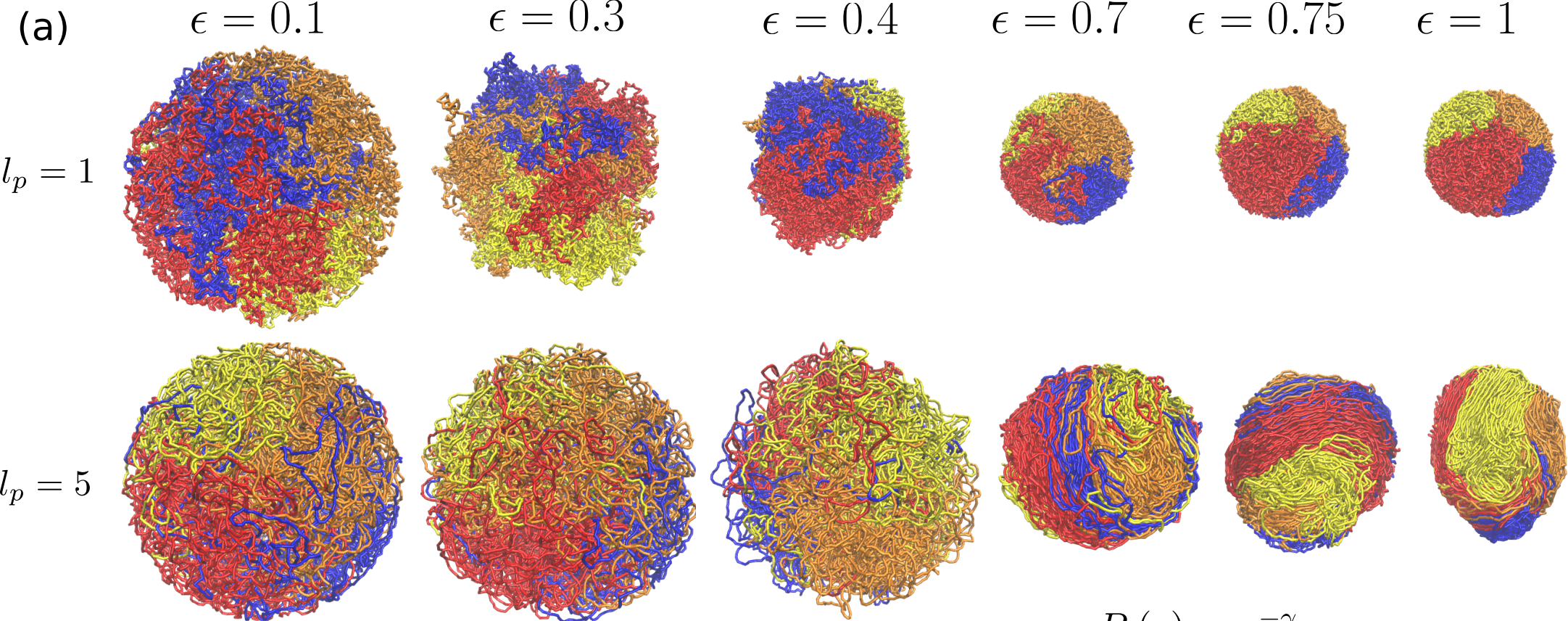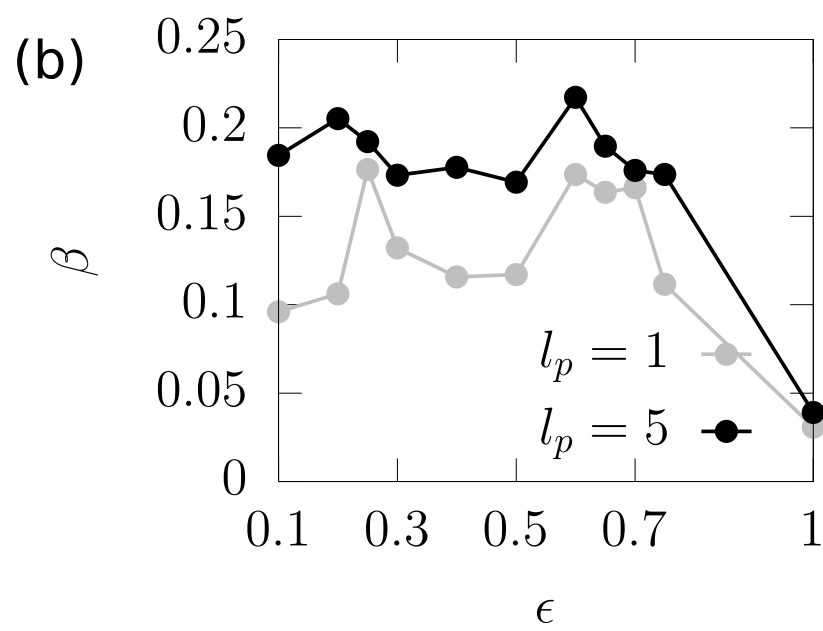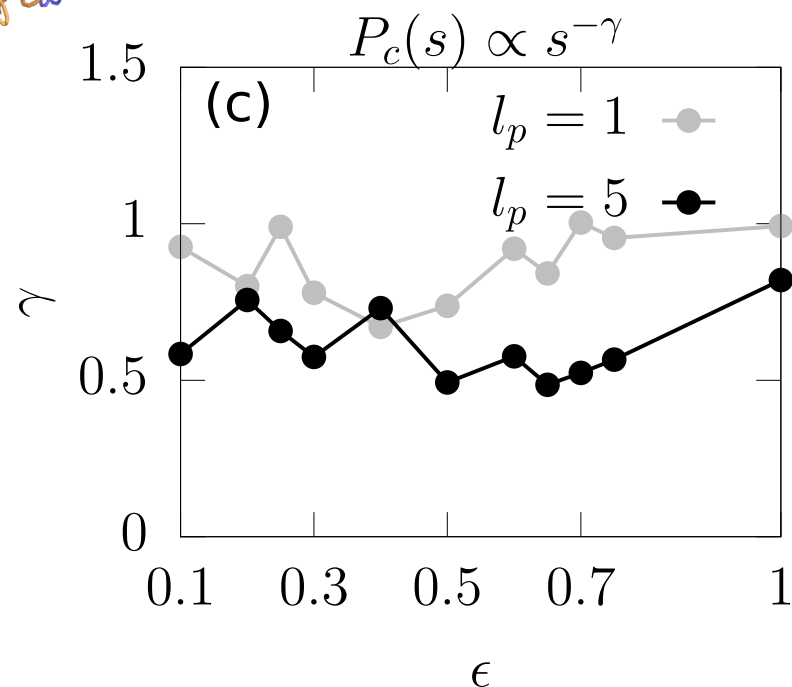

Supplement: S11 Fig — (a) Simulation snapshots of the mixing of four chains as a function of interaction strength, ϵ, for persistence lengths lp = 1 bead and lp = 5 beads. (b) The scaling exponent β of the time dependence of the chromosome mixing index. (c) The scaling exponent γ of the contact probability as a function of ϵ for lp = 1 (gray color) and lp = 5 (black color). (PDF) [file pcbi.1011142.s015.pdf]

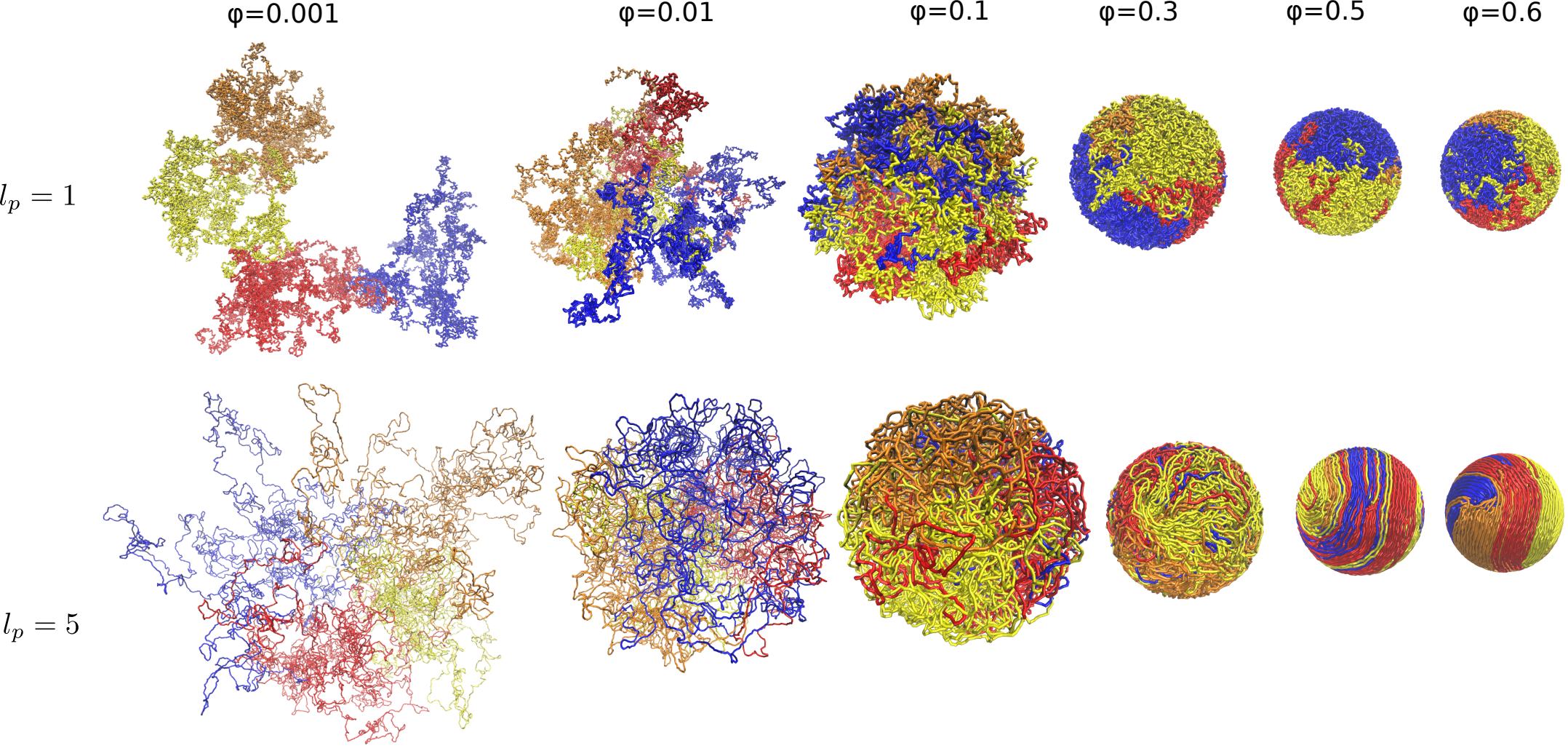

Supplement: S12 Fig — Simulation snapshots of the mixing of four chains as a function of volume fraction, ϕ, for persistence lengths lp = 1 bead and lp = 5 beads. Note that some snapshots were zoomed out because they were too large and would take up too much space if they were shown at their actual size. (PDF) [file pcbi.1011142.s016.pdf]

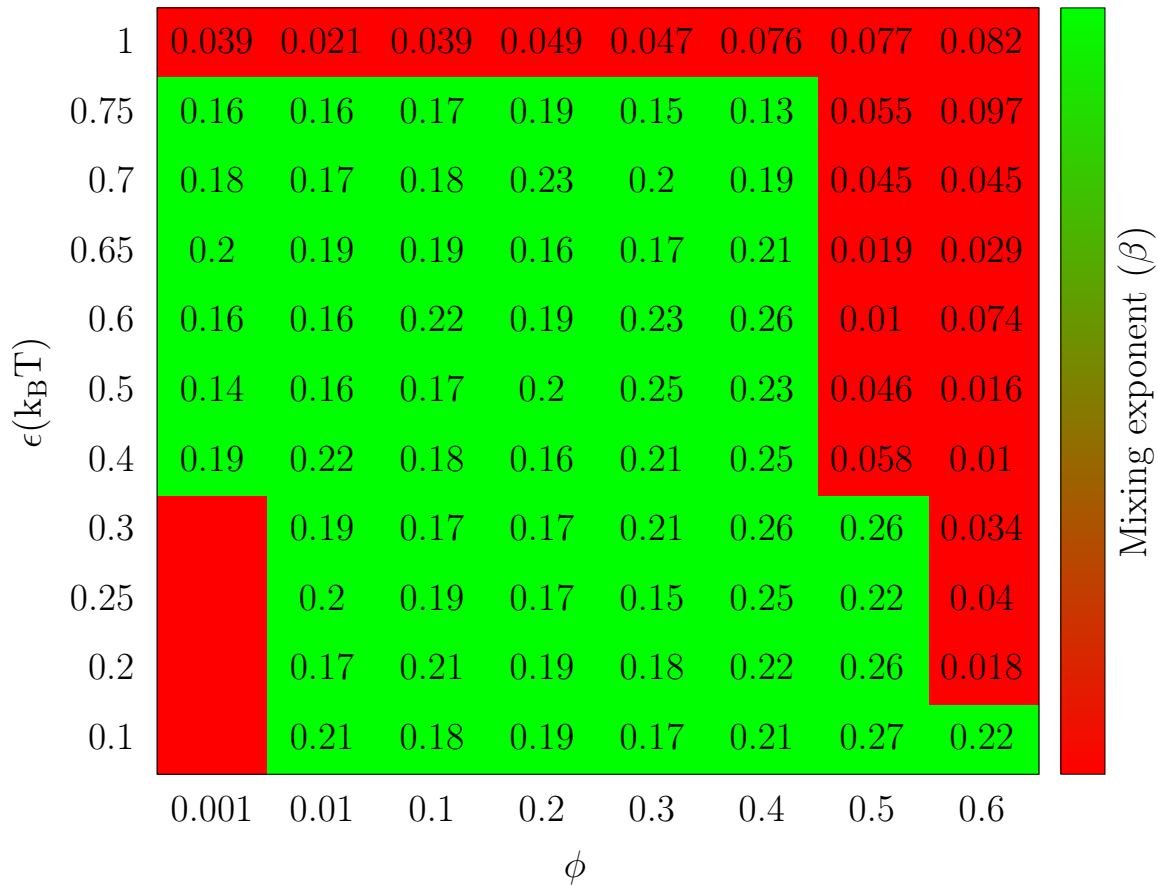

Supplement: S13 Fig — In the matrix, the value of the mixing exponent (β) is shown for each pair of volume fractions (confinement) and self-attraction (ϕ, ϵ). The matrix colors correspond to the values of β: green for β > 0.12 and red for β < 0.12. The green regions in the matrix correspond to the phase diagram in Fig 4b, which indicates that for β > 0.12, chromosomes mix within the reptation time. (PDF) [file pcbi.1011142.s017.pdf]

$\alpha = 0.22$ 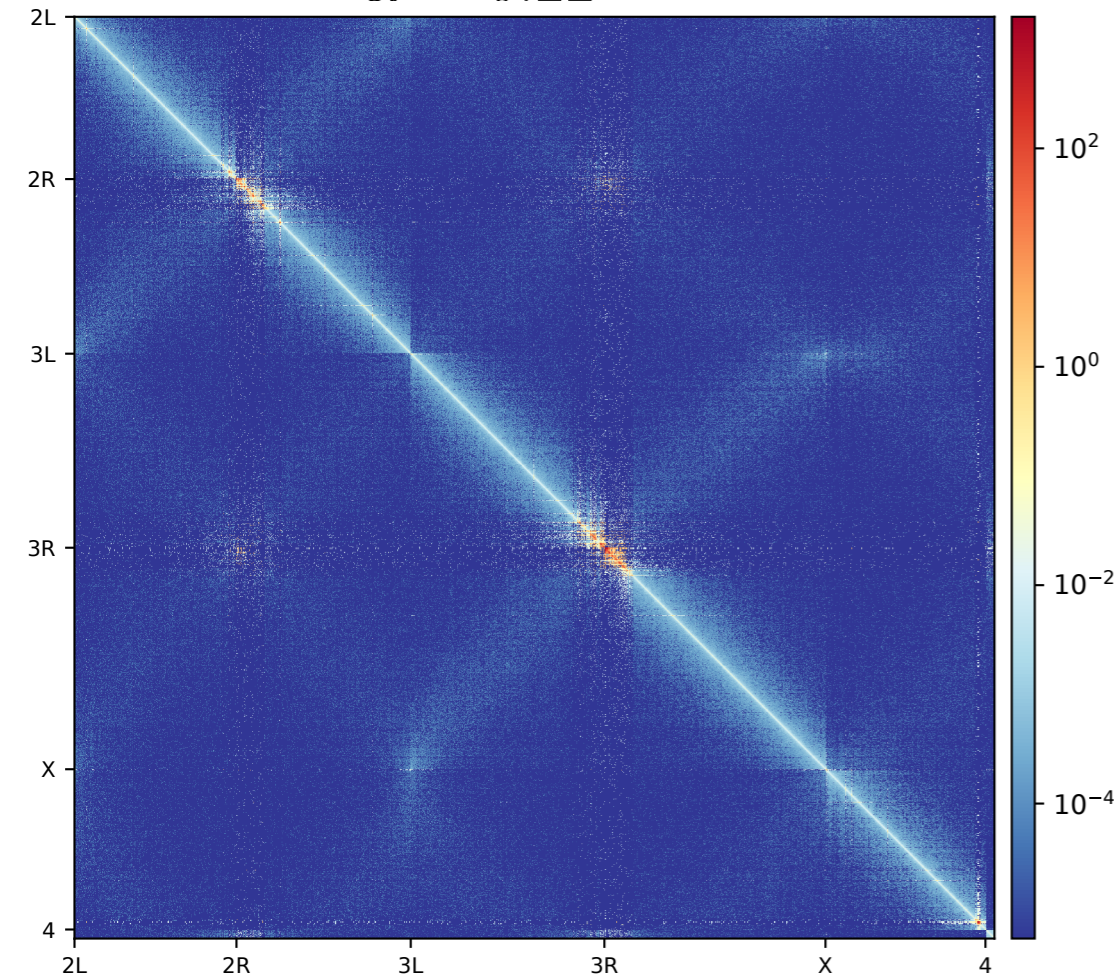

Nuclear cycle 12

 $\alpha = 0.24$ 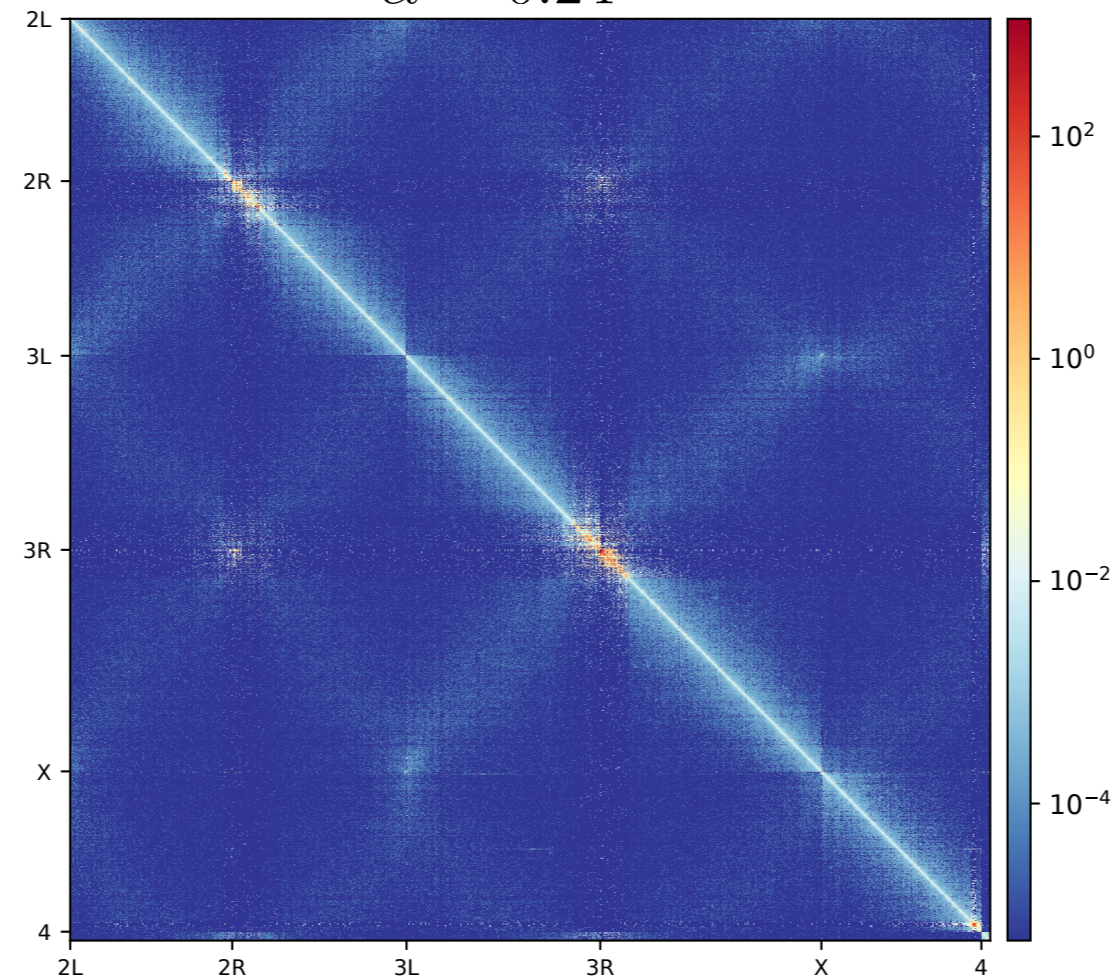

Nuclear cycle 13

 $\alpha = 0.28$ 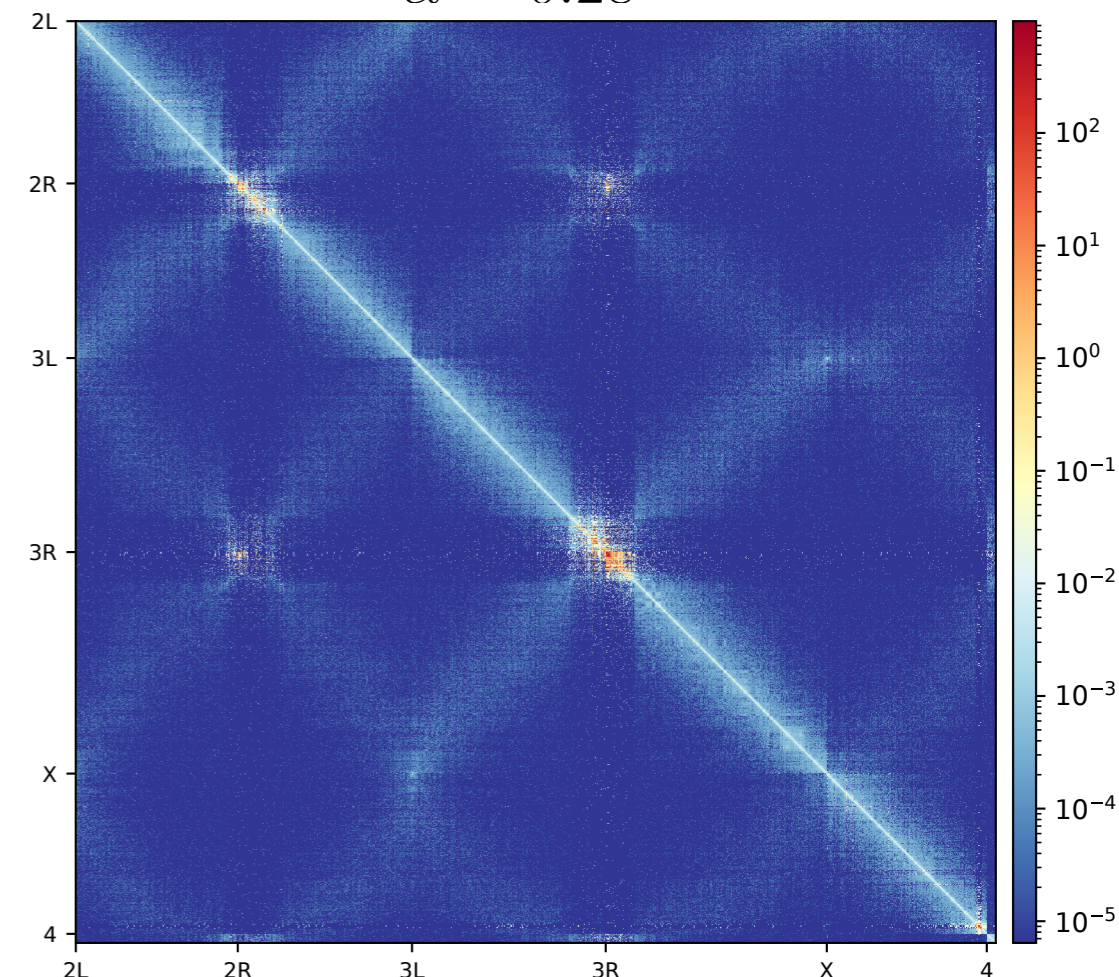

Nuclear cycle 14

Supplement: S14 Fig — The Hi-C data from the Drosophila genome [101] was used to compare the chromosome mixing index during the developmental stages of Drosophila, specifically in nuclear cycle 12 (nc12), 13 (nc13), and 14 (nc14). The comparison revealed that chromosome mixing increased progressively from nc12 to nc14. (PDF) [file pcbi.1011142.s018.pdf]

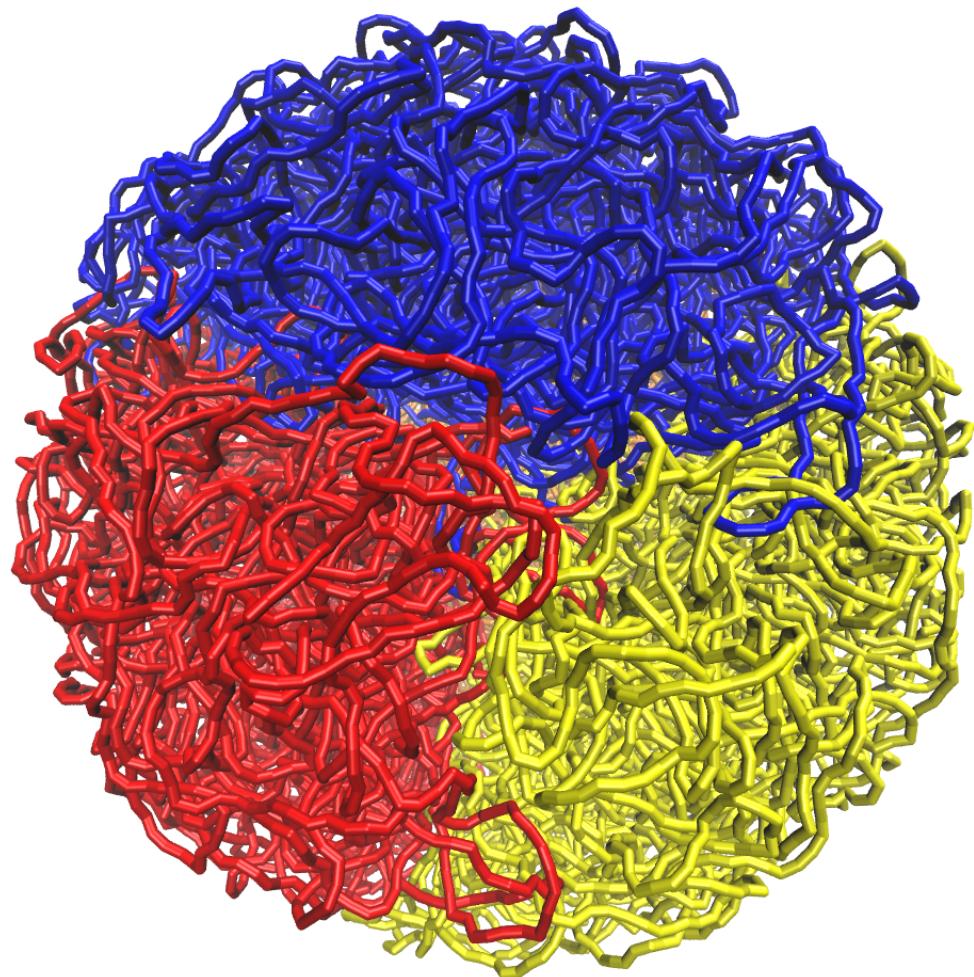

(a)

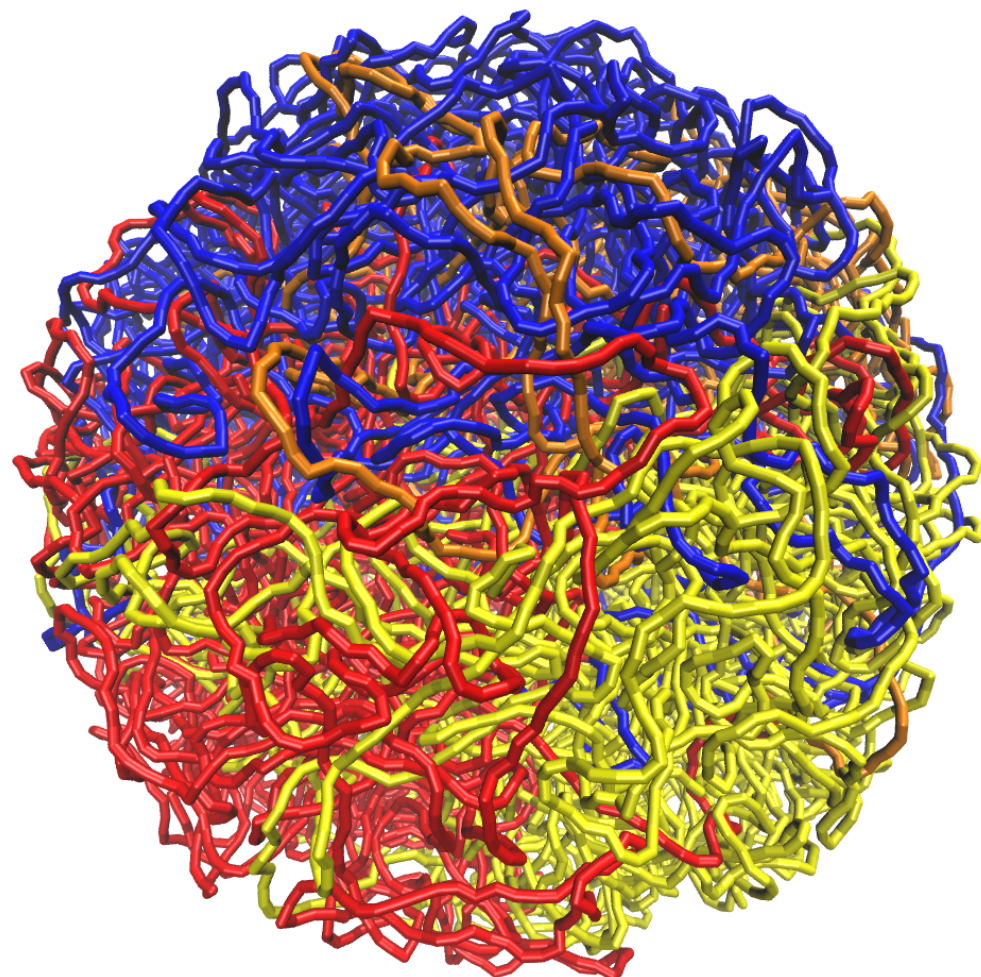

(b)

$$\epsilon_{\text{intra}} = 0.25, \epsilon_{\text{inter}} = 0.1$$

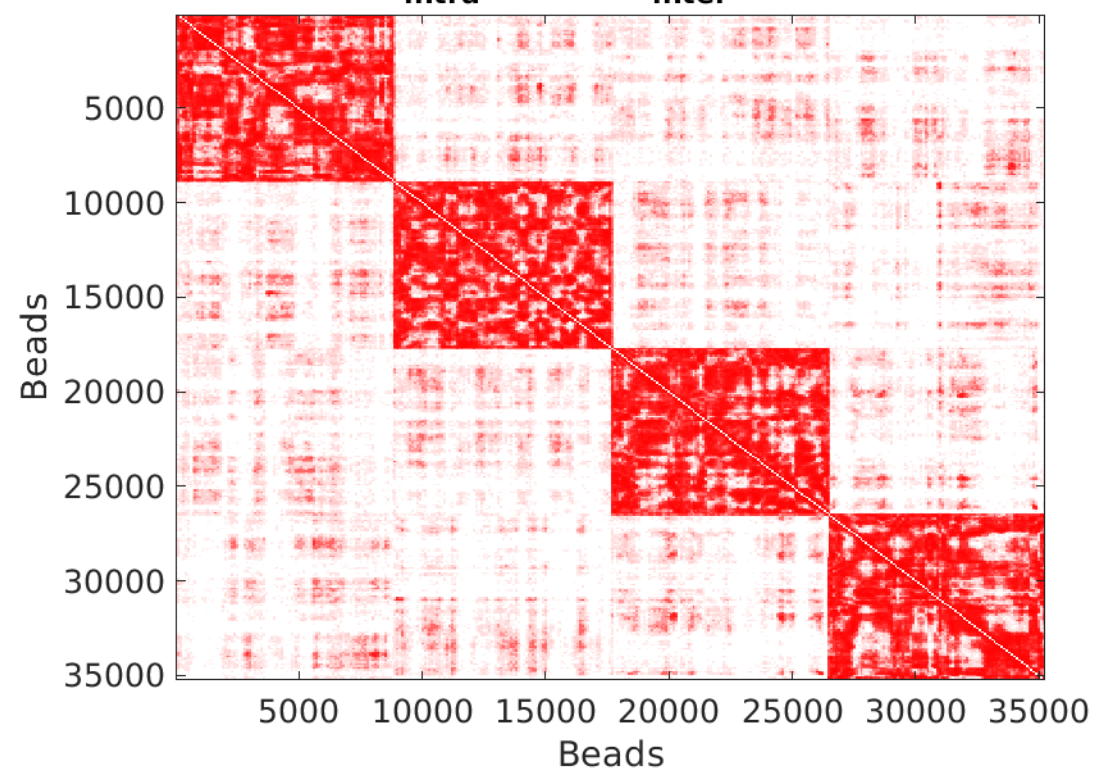

(c)

$$\epsilon_{\text{intra}} = \epsilon_{\text{inter}} = 0.25$$

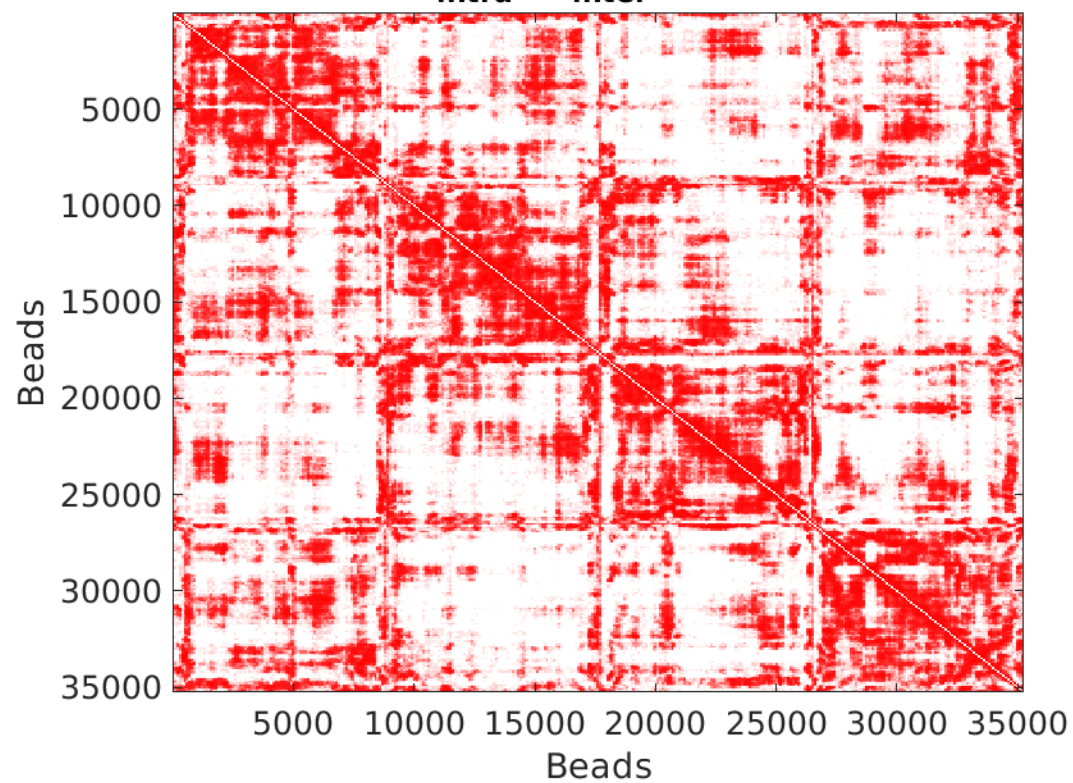

(d)

Supplement: S15 Fig — Same versus different: (a) Late time snapshot (t = 106τ) for the case where the inter-chain attraction is smaller than the intra-chromosomal attraction. In this case, the chromosomes remain phase-separated in territories and are not mixed. (b) Long time snapshot (t = 106τ) for the case where the inter-chain attraction is equal to the intra-chain attraction; this is the physically relevant situation and as time proceeds, the chains begin to mix. (c) and (d) Contact are maps calculated from the simulation for cases (a) and (b). (PDF) [file pcbi.1011142.s019.pdf]

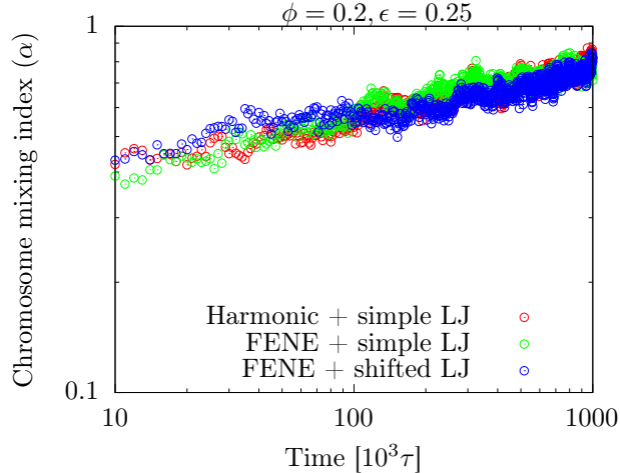

Supplement: S16 Fig — In the harmonic + simple Lennard-Jones (LJ) model, the bonded interaction between the chain’s beads is described by a harmonic potential, while the non-bonded interaction is described by a truncated LJ potential (for more information on these potentials, see the Materials and methods section). In the FENE + simple LJ model, the bonded interaction is described by a finite extensible non-linear elastic (FENE) spring, while the non-bonded interaction is described by a truncated LJ potential (for more information on the FENE potential, see the reference [48]). In the FENE + shifted LJ model, the bonded interaction is described by a FENE spring, while the non-bonded interaction is described by a Week-Chandler-Andersen (WCA) potential for excluded volume and a truncated and shifted LJ potential for attractions (for more information on the shifted LJ potential, see the reference [48]). In the figure, we see that the scaling law for the mixing index as a power of the time (obtained from the slope of the log-log plot) is very similar for all these potentials. (PDF) [file pcbi.1011142.s020.pdf]
