# Supplementary material for: Mesoscale, long-time mixing of chromosomes and its connection to polymer dynamics
Source: PLoS Comput Biol. 2023 May 25;19(5):e1011142. doi: 10.1371/journal.pcbi.1011142 (PMC10246856; doi:10.1371/journal.pcbi.1011142)
Supplement: S1 Table — (PDF) [file pcbi.1011142.s021.pdf]

S1 Table: Simulation parameters

Parameter	Description	reduced unit	SI unit
T	Temperature	1.0	310 K
$\mathrm{k_B T}$	Thermal energy	1.0	4.28×10^{-21} J
ϵ	LJ parameter	$0 - 1.0\,\mathrm{k_B T}$	
σ	Bead diameter	1.0	30 nm
R_c	Confinement radius	$20 - 164\,\sigma$	
k_{spring}	Spring constant	$100\,\mathrm{k_B T}/\sigma^2$	0.0005 $\mathrm{Jm^{-2}}$
l_p	Persistence length	$5.0\,\sigma$	
k_{bend}	Bending stiffness	$5.0\,\mathrm{k_B T}$	21.4×10^{-21} J
τ	Brownian time	$3\pi\eta\sigma^3/\mathrm{k_B T}$	60 µs
Γ	Damping coefficient	$10.0\,\tau$	
Δt	Time step	$0.01\,\tau$	

Table: We used reduced unit during the simulation.
