# Supplementary material for: Mesoscale, long-time mixing of chromosomes and its connection to polymer dynamics
Source: PLoS Comput Biol. 2023 May 25;19(5):e1011142. doi: 10.1371/journal.pcbi.1011142 (PMC10246856; doi:10.1371/journal.pcbi.1011142)
Supplement: S2 Table — (PDF) [file pcbi.1011142.s022.pdf]

S2 Table: Variation of scaling exponent of MSD

Type	Theory	Simulation LJ strength ϵ	Fits to simulation[2] $\phi = 0.4$	Fits to simulation[3] (unconfined)
Disconnected noninteracting beads	$\lambda = 1$	$\epsilon = 0$	$\lambda = 1$	
Disconnected interacting beads [1]	$\lambda = 3/4$	$\epsilon = 0.25, 0.5$	$\lambda = 0.8$	
Phantom polymer (Rouse dynamics [2])	$\lambda = 1/2$	$\epsilon = 0$	$\lambda = 0.5$	$\lambda = 0.5$
Polymer in good solvent[1]	$\lambda = 6/11$	$\epsilon = 0.25$	$\lambda = 0.4$	$\lambda = 0.5$
Polymer in poor solvent[1]	$\lambda = 2/5$	$\epsilon = 0.5$	$\lambda = 0.4$	$\lambda = 0.25$
Polymer melts (reptation [3])	$\lambda = 1/4$	$\epsilon = 1$		$\lambda = 0.25$

[1] Polymer of length M that is confined in a space has scaling exponent of MSD $\lambda = \frac{2\nu}{2\nu+1}$, where ν is related with MSD $\sim M^{2\nu}$ [4].

[2] S7 Fig

[3] S5 Fig

Table: MSD, $\langle r^2 \rangle \sim \tau^\lambda$

References

[1] Bénichou O, Illien P, Oshanin G, Sarracino A, Voituriez R. Diffusion and subdiffusion of interacting particles on comblike structures. Physical review letters. 2015;115(22):220601.

[2] De Gennes PG. Quasi-elastic scattering of neutrons by dilute polymer solutions: I. Free-draining limit. Physics Physique Fizika. 1967;3(1):37.

[3] De Gennes PG. Reptation of a polymer chain in the presence of fixed obstacles. The journal of chemical physics. 1971;55(2):572–579.

[4] Liu L, Shi G, Thirumalai D, Hyeon C. Chain organization of human interphase chromosome determines the spatiotemporal dynamics of chromatin loci. PLoS computational biology. 2018;14(12):e1006617.
