# Supplementary material for: Mesoscale, long-time mixing of chromosomes and its connection to polymer dynamics
Source: PLoS Comput Biol. 2023 May 25;19(5):e1011142. doi: 10.1371/journal.pcbi.1011142 (PMC10246856; doi:10.1371/journal.pcbi.1011142)
Supplement: S3 Table — (PDF) [file pcbi.1011142.s023.pdf]

S3 Table: Comparison between scaling exponent of RMSD and scaling exponent of mixing index

Type	LJ strength ϵ	$\lambda/2$ (for $\phi = 0.4$)	$\lambda/2$ (unconfined)	β (for $\phi = 0.4$)
Polymer in good solvent	$\epsilon = 0.25\,\mathrm{k_B T}$	$\lambda/2 = 0.2$	$\lambda/2 = 0.25$	$\beta = 0.25$
Polymer in poor solvent	$\epsilon = 0.5\,\mathrm{k_B T}$	$\lambda/2 = 0.2$	$\lambda/2 = 0.125$	$\beta = 0.22$
Polymer melts (reptation)	$\epsilon = 0.75\,\mathrm{k_B T}$		$\lambda/2 = 0.125$	$\beta = 0.13$
Polymer melts ("jammed")	$\epsilon = 1\,\mathrm{k_B T}$		$\lambda/2 = 0.125$	$\beta = 0.076$

Table: RMSD ($\sqrt{\langle r^2 \rangle} \sim \tau^{\lambda/2}$) and chromosome mixing index ($\alpha \sim t^{\beta}$)
