# Supplementary material for: Mesoscale, long-time mixing of chromosomes and its connection to polymer dynamics
Source: PLoS Comput Biol. 2023 May 25;19(5):e1011142. doi: 10.1371/journal.pcbi.1011142 (PMC10246856; doi:10.1371/journal.pcbi.1011142)
Supplement: Attachment — Submitted filename: PCOMPBIOL-D-22-01474_report.pdf [file pcbi.1011142.s030.pdf]

The authors in this work studied quantitatively the mixing of chromosome, an interesting yet often overlooked process after existing mitosis, based on simulations of a generic coarse-grained polymer model. They proposed a chromosome mixing index ($\alpha$) which increases with time in a power law ($\alpha \sim t^{\beta}$). To disentangle the effect of different factors on mixing, they raised the complexity of the polymer model step by step from phantom chains to more realistic model of Drosophila genome, analyzed how chromosome mixing depends on self-attraction, confinement, and heterogeneity in the bead-bead and bead-boundary interactions. In addition, the mixing index was discussed referring to the diffusion exponent (mean-square displacement, MSD$\sim t^{\lambda}$) and the decay exponent of contact probability ($P(s) \sim s^{-\gamma}$). The numeric experiments are well designed, and the well-organized article deepens our understanding of chromosome dynamics. I have following questions and comments.

1) The upper bound of mixing index deserves some discussion. In S2 Text, the authors claimed that the maximum value of mixing index $\alpha_{max} = n - 1$, where $n$ is the total number of chromosomes. However, based on Eq. (1, 2) in S2 Text, the mixing index will become larger than $\alpha_{max}$ if the total intrachromosomal contacts is less than $n \times M \times M$. Considering an extreme case where the system is composed of polymers which behave as rigid rods, $\alpha$ will diverge since there is no intrachromosomal contact.

2) In the first subsection of Results (page #7), the authors analyzed the dependence of mixing of phantom chains on confinement. Note that the chain length ($M$) may also affect the value of $\alpha$ at equilibrium. As the monomer density decrease as $M^{-1/2}$ [12], it is expected that $\alpha$ increases with $M$, at least in the dilute limit (i.e., at small $\phi$).

3) In Fig. 2 (b) and Fig. 3 (b), the mixing index seems to have two scaling regions at $\phi = 0.6$, which may correspond to two different microscopic mixing mechanisms. Some comments can be added on this point.

4) In the fourth subsection of Results (page #16), the completely mixing time ($t$) was estimated based on $\alpha(t) \approx \alpha_{max} = 3$. It was assumed that $\alpha$ will approach 3 at long time limit, which however is not guaranteed. For example, as shown in Fig. 1 (b), the phantom chains in confinement of $\phi = 0.01$ has a chromosome mixing index $\alpha < 2$ at equilibrium. This may explain the extremely slow mixing time (red pixels) at small $\epsilon$ and small $\phi$ in Fig. 4 (a).

5) The relationship between $\beta$, $\gamma$, and $\lambda$ is intriguing. Although the estimated value of $\beta$ is close to that of $\lambda/2$ (as shown by S3 Table), the underlying reason remains unclear. Previous work has suggested a link between the scaling exponent of the gyration radius ($\nu$) and $\lambda$ [70]. I would like to draw the authors' attention to a related reference (Rep. Prog. Phys. 77 (2014) 022601), which analyzed the contacts between subchains theoretically.

6) In Line #251-255, the authors briefly mentioned that "in our results we see evidence of no knots". It would be better if they specify what the evidence is. I cannot tell whether the chains are knotted or not by looking at their configurations.

7) As for the initial state of the simulations, there are many possible ways to prepare separated unknotted chains in spherical confinement. To resolve the ambiguity, I suggest the authors adding a snapshot of the initial state or a supplementary movie of the whole mixing process.

8) In Figs. 1 (c), 2 (d), and 3 (d), the minimum value of the contour length ($s$) shown on the $x$-axis is 200 beads due to the coarse graining procedure of contact matrix (S3 Text). How about $P_c(s)$ at $1 < s < 200$?

9) If I understand correctly, the contact matrices shown in Figs. 1 (f), 2 (c), 3 (c), and 5 (c,f) were calculated as an average over simulation time from *a single* trajectory. This should be emphasized in the Materials and Methods section, as we typically perform several independent simulations and present the *ensemble*- and time-averaged results. In addition, the division of different chromosomes or eu-/hetero-chromatin is not clear in the contact matrix heatmap. Adding dividing lines, like Fig. 1 (a), can be helpful.

10) The sentence in Line #315-316 is opposite to what is shown in Fig. 1 (f,g). Moreover, the heterogenous pattern of contact map at $\phi = 0.001$ may result from insufficient sampling, as implied by the fact that the chromosome mixing index did not reach equilibrium at the maximum simulation time for $\phi = 0.001$ (see Fig. 1 (b)).

11) In Fig. 4 (a), the $(\phi, \epsilon)$ pairs at (0.01, 0.2), (0.001, 0.75) and (0.3, 0.75) *do* have a yellow or orange color, which disagrees with the argument in Line #473.

12) In Line #515, how about interaction strength between euchromatic and heterochromatic beads, $\epsilon_{\mathrm{EH}}$?

13) Please fix the typos: "the number intra-chromosomal" in Line #74; "the extent chromosome mixing" in Line #98; "we also the mixing within a single chain by calculating" in Line #100; "and shown that" in Line #124; "In Section, we" in Line #169; "as functions of $\phi$" in Line #368; "between the mixing order times" in the caption of Fig. 4; the comma in Line #565 can be deleted.
