# Supplementary material for: Mesoscale, long-time mixing of chromosomes and its connection to polymer dynamics
Source: PLoS Comput Biol. 2023 May 25;19(5):e1011142. doi: 10.1371/journal.pcbi.1011142 (PMC10246856; doi:10.1371/journal.pcbi.1011142)
Supplement: Attachment — Submitted filename: Referees_Response_Bajpai_etal.pdf [file pcbi.1011142.s031.pdf]

# Response to reviewers' comments

We would like to thank the reviewers for their valuable and insightful comments. We are pleased to see that two out of three reviewers appreciated our work and its connections to polymer physics. In response to the comments, we have conducted additional simulations and revised the manuscript extensively to address all concerns. We have modified figures in the main text of the manuscript and included several new figures in the supplementary text. The modifications have been marked in red in the revised manuscript.

## Response to reviewer 1

**Reviewer 1 said:** *The authors in this work studied quantitatively the mixing of chromosome, an interesting yet often overlooked process after existing mitosis, based on simulations of a generic coarse-grained polymer model. They proposed a chromosome mixing index ($\alpha$) which increases with time in a power law ($\alpha \sim t^{\beta}$). To disentangle the effect of different factors on mixing, they raised the complexity of the polymer model step by step from phantom chains to more realistic model of Drosophila genome, analyzed how chromosome mixing depends on self-attraction, confinement, and heterogeneity in the bead-bead and bead-boundary interactions. In addition, the mixing index was discussed referring to the diffusion exponent (mean-square displacement, $MSD \sim t^{\lambda}$) and the decay exponent). The numeric experiments are well designed, and the well-organized article of contact probability ($P(s) \sim s^{-\gamma}$ deepens our understanding of chromosome dynamics. I have following questions and comments.*

**Our response:** We are very grateful that you appreciate our work and its scientific spirit. The revisions that are in large part due to your excellent comments, will further enhance our paper and make it more understandable.

**Reviewer 1 said:** *1) The upper bound of mixing index deserves some discussion. In S2 Text, the authors claimed that the maximum value of mixing index $\alpha_{max} = n - 1$, where n is the total number of chromosomes. However, based on Eq.(1,2) in S2 Text, the mixing index will become larger than $\alpha_{max}$ if the total intrachromosomal contacts is less than $n \times M \times M$. Considering an extreme case where the system is composed of polymers which behave as rigid rods, $\alpha$ will diverge since there is no intrachromosomal contact.*

**Our response:** We agree with the reviewer's comment that the chromosome mixing index $\alpha_{max} = n - 1$ is not applicable to systems with rigid rods. Our aim was to find a chromosome mixing index for chromosome-like long polymers in which the persistence length of the polymer is much shorter than its total length. In revised manuscript, we added this limitation of our theoretical calculation on chromosome mixing index.

We added following lines in the text in section S2 Text of the supporting information (SI).

"In the calculation of the chromosome mixing index, the number of intrachromosomal contacts enters into the denominator. Therefore, if total intrachromosomal contacts are zero, as in the case of rigid rods, the mixing index will not be applicable. The chromosome mixing index is most appropriate for long polymers whose persistence length is significantly smaller than their total length ($l_p << L$) so that intrachromosomal contacts within a single polymer will be formed; the confined environment of the nucleus also increases this tendency. This is because in such a scenario, the chromosomes are able to mix effectively without diffusing too much in the larger space."

**Reviewer 1 said:** *2) In the first subsection of Results (page #7), the authors analyzed the dependence of mixing of phantom chains on confinement. Note that the chain length (M) may also affect the value of $\alpha$ at equilibrium. As the monomer density decrease as $M^{-1/2}$[12], it is expected that $\alpha$ increases with $M$, at least in the dilute limit (i.e., at small $\phi$).*

**Our response:** We agree with the reviewer's comments that chain length also affects the value of $\alpha$. That is why, we introduced the dimensionless quantity, the chromosome volume fraction in the nucleus ($\phi$). The value of $\phi$ is dependent on both the length of the polymer and the radius of confinement in which the polymer is located. We note that our focus is on chromosomes confined to the nucleus and the very dilute limit, while important for understanding, is not that relevant. In our phantom chain simulations, we varied $\phi$. Our simulations showed that when $\phi$ is relatively small, the chromosome mixing index does not reach its maximum value. We found that when the radius of gyration of the polymers exceeds the diameter of confinement, the confinement has a significant effect and the chromosome mixing index reaches its maximum value, i.e., $\alpha = n - 1$ (for phantom chains). This indicates that chromosomes are completely mixed when the density is high, which is consistent with the relatively high chromatin density observed within the cell nucleus.

To support our findings, we have included a new figure (Fig.S4) that illustrates the relationship between the mixing index of chromosomes and the size and confinement of polymers. The figure shows that the mixing index is maximized when the ratio of the diameter of confinement ($2R_c$) to the radius of gyration ($R_g$) of an unconfined chain is less than one ($2R_c/R_g < 1$), demonstrating the influence of the size and confinement of the polymers on the mixing index.

Old text: We can easily understand this by comparing the radius of gyration and the diameter of confinement. The chromosome mixing index of phantom chains (with radius of gyration $R_g$) reaches its maximal value for relatively small confinement volumes $R_c$,

$R_g \gg R_c$ but has a small value when $R_g \ll R_c$.

Revised text: In Fig.S4, the ratio of the confinement size (diameter, $2R_c$) to the radius of gyration ($R_g$) of the phantom chain is plotted for various values of $\phi$. The results indicate that when $\phi \leq 0.01$, the confinement volume is relatively large, as $2R_c/R_g > 1$. Conversely, when $\phi \geq 0.1$, the confinement volume is relatively small, as $2R_c/R_g < 1$, and the chromosome mixing index for phantom chains reaches its maximum for these values of $\phi$.

**Reviewer 1 said:** *3) In Fig. 2 (b) and Fig. 3 (b), the mixing index seems to have two scaling regions at* $\phi = 0.6$*, which may correspond to two different microscopic mixing mechanisms. Some comments can be added on this point.*

**Our response:** We agree with the reviewer's comments that there are two scaling regions for these cases. The second region shows that mixing slows down with time. However, in both cases, the exponent of the chromosome mixing index, $\beta$, is less than or equal to 0.1 ($\beta <= 0.1$), as indicated by the red color in the heatmap of mixing times in Fig. 4a, indicating slow mixing which might then evolve into a jammed state that can mix no further.

As suggested by the reviewer, we now show by a dashed and solid line the early and late time exponents of $\beta$ in revised versions of Fig. 2(b) and Fig. 3(b). Including the second exponent did not change our main results in the state diagram of Fig. 4b since both regimes mix so slowly that mixing does not occur on any practical time scale. We attribute the extremely small second exponent 0.04. to the "jamming" that occurs when a very small confinement or very high attraction strength increases the density of molecules so much that they are kinetically prevented from mixing. This is explained further in the revised manuscript.

Starting on line 371 of the revised manuscript, we have added the following sentences.

"Fig.2 (b) for $\epsilon = 1$ shows two mixing exponents, $\beta_1$ and $\beta_2$, indicating that the beads begin to mix at early times with a mixing exponent of $\beta_1 = 0.14$, while at late times, the mixing exponent is very close to zero ($\beta_2 = 0.04$), indicating a "jammed" situation."

Starting on line 429 of the revised manuscript, we have added the following sentences.

"For high volume fractions ($\phi \geq 0.6$), there are two mixing exponents: $\beta_1 = 0.19$ for short mixing times and $\beta_2 = 0.04$ for at longer time; this exponent indicates restricted motion of the chains due to the very small confinement volume."

The captions of Fig.2 and Fig.3 of the revised manuscript have also been updated.

**Reviewer 1 said:** *4) In the fourth subsection of Results (page #16), the completely mixing time (t) was estimated based on* $\alpha(t) = \alpha_{max} = 3$*. It was assumed that* $\alpha$ *will approach 3 at long time limit, which however is not guaranteed. For example, as shown*

*in Fig. 1 (b), the phantom chains in confinement of $\phi = 0.01$ has a chromosome mixing index $\alpha < 2$ at equilibrium. This may explain the extremely slow mixing time (red pixels) at small $\epsilon$ and small $\phi$ in Fig. 4 (a).*

**Our response:** Thank you for bringing this issue to our attention. You are correct that the chromosome mixing index may not approach its maximal value of 3 at the long time limit, as shown in Fig. 1(b) for the case of phantom chains in confinement with $\phi = 0.01$. For such a large confinement (very small chain volume fraction – at least relative to the biological case), the chains can diffuse away from each other and not mix.

We have made the necessary revisions to the manuscript to address this issue. As previously discussed, when $R_c << R_g$, the chromosome mixing index will not reach its maximal value. In Fig.S4, we have plotted $2R_c/R_g$ as a function of the volume fraction and shown that only when $2R_c/R_g < 1$, the chromosome mixing index will reach its maximal value. Additionally, we have clarified in the text that when $2R_c/R_g > 1$, the power exponent is undefined ($\beta \approx 0$), indicating a diffusive region where the chromosomes will never fully mix. Since the mixing time goes to infinity for $\beta = 0$, this does not change our main results in the phase diagram of Fig. 4b.

Please see above, where we first refer to this new figure Fig.S4 and the revised text. Starting on line 469 of the revised manuscript, we have added the following sentences in the fourth subsection of results.

"When the attraction is weak ($\epsilon \leq \epsilon_c$), the chains do not reach equilibrium if the volume fraction is low ($\phi \leq 0.001$) or ($2R_c/R_g > 1$ in Fig.S4 (b)). This is because the chains diffuse in the large confinement volume and never fully mix. The extrapolation time goes to infinity since the mixing exponent $\beta \approx 0$. These regions are represented by red colors (lower left regions) in the heatmap, as any mixing that takes longer than $t = 10^{16}\tau$ is considered red."

**Reviewer 1 said:** *5) The relationship between $\beta$, $\gamma$, and $\lambda$ is intriguing. Although the estimated value of $\beta$ is close to that of $\lambda/2$ (as shown by S3 Table), the underlying reason remains unclear. Previous work has suggested a link between the scaling exponent of the gyration radius ($\nu$) and $\lambda$ [70]. I would like to draw the authors' attention to a related reference (Rep. Prog. Phys. 77 (2014) 022601), which analyzed the contacts between subchains theoretically.*

**Our response:** In the revised manuscript starting at line 510, we wrote a few lines to support how mixing index is related to RMSD:

"We note that the mixing at the lowest level depends on the motion of individual beads in their environment of other chains and confinement. The correspondence of the chromosome mixing index $\alpha$ and the RMSD time dependence for the motion of a bead shows that the coarse-grained $\alpha$ has dynamics that follows a simple mean-field behavior of the single bead dynamics, but appropriately modified so that the RMSD exponent

accounts for the environment of the bead; this depends on the attraction strength and the overall confinement volume (bead volume fraction)."

Starting line 343, we added lines related to previous work on relation between $\nu$ and $\lambda$.

"High-throughput chromatin motion tracking experiments in living yeast cells have revealed that the mean squared displacement (MSD) of chromosomes follows a scaling law of MSD $\sim t^{1/2}$, indicating that they obey random walk statistics [1, 2]. However, the MSD can be influenced by the organization and confinement of chromosomes and can vary among cells and cell types [3]. The scaling law for MSD of a polymer is $\mathrm{MSD}(t) \sim t^{2\nu/(2\nu+1)}$, where $\nu = 1/2$ for a random walk chain and $\nu = 1/3$ for a collapsed, self-attractive chain, a chain in a poor solvent, or a chain in a small confinement volume. Our study (shown in Fig.S1) demonstrates how chromatin-chromatin interactions affect the MSD. In Fig.S11, we calculated the average mean-squared displacement (MSD) of four chains over time for different attraction strengths, $\epsilon$. The range of mixing exponents $\beta$ is consistent with Rouse dynamics for weak interactions and a smaller exponent for stronger interactions [3–5]."

In the caption of Fig.S5, we have included information on the mean field relation, $P(s) \sim r^{-3}(s)$, where $r(s)$ is the subchain size [6].

**Reviewer 1 said:** *6) In Line #251-255, the authors briefly mentioned that "in our results we see evidence of no knots". It would be better if they specify what the evidence is. I cannot tell whether the chains are knotted or not by looking at their configurations.*

**Our response:** Starting on line 637 of the revised manuscript, we have rewritten the sentences including the evidence.

Old text: In our results we see evidence of no knots in two important limiting cases (i) phantom chains (see Fig.1) that can be thought of as being dominated by chromosome cutting enzymes (e.g. Type II topoisomerase [7]). (ii) Strongly attractive chromatin [8,9] where we show (see Fig.2 and Fig.3) that the steady state structure is that of a fractal globule which again has no knots [10–12].

Revised text: Even though we began simulations with unknotted chains, chain mixing can result in knots forming between chains. Type II topoisomerases enzymes are known for removing DNA knots [7]. In our results, we found that there are no knots in two important limiting cases: (i) phantom chains (see Fig.1) that can be thought of as being dominated by chromosome cutting enzymes (e.g. Type II topoisomerase [7]). These chains easily mix in relatively small confinement volumes, but completely unmix when the confinement volume is increased, indicating a lack of knots. (ii) Strongly attractive

chromatin [8, 9] where we show (see Fig.2 and Fig.3) that the steady state structure is that of a fractal globule which again has no knots [10–12]. This can be understood by coloring subchains with different colors, and observing that when these subchains remain separated, the structure is unknotted [13]. Our results in Fig.S1 (a) for the case of $\epsilon = 1$ and persistence lengths of 1 and 5 beads, show that the subchains correspond to different colors are separated.

**Reviewer 1 said:** *7)As for the initial state of the simulations, there are many possible ways to prepare separated unknotted chains in spherical confinement. To resolve the ambiguity, I suggest the authors adding a snapshot of the initial state or a supplementary movie of the whole mixing process.*

**Our response:** We have now added the snapshots in new Figs. S2 and S3. To prepare the separated unknotted chains in spherical confinement for our simulations, we followed the following protocol:

1. We used the moltemplate software and python code *interpolate_coords.py* [14] to generate an initial configuration of a single chain with 8810 beads. This code creates a smooth polymer structure within a cubic box using cubic spline interpolation (see Fig.S2 (a), (b)).

2. To confirm that the generated initial configuration was unknotted, we performed a self-avoiding chain simulation with a persistence length of 1 bead and a simple Lennard-Jones potential with a strength of $\epsilon = 1k_BT$ and a cutoff of $r_c = 2^{1/6}\sigma$. This generated an open chain structure, which is not possible when the initial structure is knotted (see Fig.S2 (c)).

3. We used the packmol software [15] to generate four copies of the chain in a cubic box with a size of $L = 100\sigma$.

4. We then compressed the polymers system to a small spherical confinement radius of $R_c = 20\sigma$ (or $\phi = 0.6$) using indented walls and Lennard-Jones interactions (see Fig.S3). The force exerted by a spherical indenter on each bead is represented by the equation $F(r) = -K_{\text{indent}}(r - R_{indent})^2$, where $K_{\text{indent}}$ is the specified force constant, $r$ is the distance from the bead to the center of the indenter, and $R_{\text{indent}}$ is the radius of the indenter [14]. The inter-chain interactions were repulsive during compression, with a strength of 1 $k_BT$ ($\epsilon_{\text{inter}} = 1k_BT$) and a cutoff distance of $2^{1/6}$ sigma ($r_c = 2^{1/6}\sigma$). The intra-chain interactions were attractive, with a strength of 1 $k_BT$ ($\epsilon_{\text{intra}} = 1k_BT$) and a cutoff distance of $2.5\sigma$ ($r_c = 2.5\sigma$).

New figures Fig.S2 and Fig.S3 contain the snapshots of the initial state and their captions include the protocol listed above. We also included a new supplementary movie

of the entire mixing process in the revised version of the manuscript for clarity. We hope that these additions will address your concerns and provide a better understanding of our simulations.

Starting on line 224 of the revised manuscript, we have added the following sentences.

"For this biological reason and to obtain a lower bound on the mixing time, we used unknotted chromosome chains as the initial condition. Using moltemplate software [14], we created a single chain of 8810 beads and confirmed that it was unknotted (see the details in Fig.S2). We then placed four copies of this chain in a cubic box and compressed them into a small spherical confinement using indented walls [16]. The Lennard-Jones interactions between the intra-chain were attractive, while the inter-chain interactions were repulsive in the creation of the initial structure. This can be seen in Fig.S3, which illustrates the initially separated and condensed 4 chains in a confinement."

**Reviewer 1 said:** *8) In Figs. 1 (c), 2 (d), and 3 (d), the minimum value of the contour length (s) shown on the x-axis is 200 beads due to the coarse graining procedure of contact matrix (S3 Text). How about* $P_c(s)$ *at* $1 < s < 200$*?*

**Our response:** Thank you for your question about the contour length range shown in Figs. 1(c), 2(d), and 3(d). You are correct that the minimum value of the contour length shown on the x-axis is 200 beads due to the coarse graining procedure used to calculate the contact matrix (as described in S3 Text). In the revised manuscript, we have also analyzed the simulation data with a finer grained resolution and calculated the contact probability ($P_c(s)$) for the range of 1 to 200 beads.

In our simulations, we have 4 chains: We first calculated the contact probability for each chain and then took the average, so the final plot shows the average contact probability of the 4 chains. Since the attraction between the intra-chain and inter-chain interactions is the same and each chain has the same length, the contact probability of the first chain ($P_1(s)$) is similar to the average contact probability ($P_c(s)$).

In the new Fig.S5, we have plotted the contact probability for a range of 1 to 8000 beads. For relatively small confinement (green curve), the contact probability shows two regions: a power law for 1 to 200 beads, followed by saturation for values greater than 200 beads. Because we focused on the saturation, we had plotted the range of $s > 200$ to compare the contact probabilities.

Starting on line 276 of the revised manuscript, we have added the following sentences.
"We have also calculated scaling exponent $\gamma$ for the range $1 \leq s \leq 8000$, which shows two exponents (at early and late times) when the confinement is small, and one exponent when the confinement is large (see Fig.S5)."

**Reviewer 1 said:** *9) If I understand correctly, the contact matrices shown in Figs. 1 (f), 2 (c), 3 (c), and 5 (c,f) were calculated as an average over simulation time from a single trajectory. This should be emphasized in the Materials and Methods section, as we typically perform several independent simulations and present the ensemble- and time-averaged results. In addition, the division of different chromosomes or eu-/hetero-chromatin is not clear in the contact matrix heatmap. Adding dividing lines, like Fig. 1 (a), can be helpful.*
**Our response:** In Section S3 Text, we already explained in detail how we calculated the contact map, contact probability, and chromosome mixing index from simulation data.

To make the division of different chromosomes in the contact matrix heatmaps clearer, we have added along the axes of these maps, colored lines that correspond to the colors of the chains in Fig. 1 (f), (g), Fig. 2 (c), Fig. 3 (c), Fig. 5 (c), (f), and Fig. 6 (c), (d).

In the revised manuscript, Fig.6's caption, we have added the following sentence.

"Note that in the contact maps, vertical and horizontal colored lines are added to distinguish the chains, which correspond to the colors of the chromosomes."

**Reviewer 1 said:** *10)The sentence in Line #315-316 is opposite to what is shown in Fig. 1 (f,g). Moreover, the heterogenous pattern of contact map at* $\phi = 0.001$ *may result from insufficient sampling, as implied by the fact that the chromosome mixing index did not reach equilibrium at the maximum simulation time for* $\phi = 0.001$ *(see Fig. 1 (b)).*
**Our response:** We apologize if any confusion has been caused by the original manuscript. We have revised those lines to clarify that we meant "larger confinement radius" (small polymer volume fraction with a heterogeneous map, due to polymer diffusion) and "smaller confinement radius" (larger polymer volume fraction) with a homogeneous map. With regard to the case of phantom chains, where there is no inter-chain attraction, we acknowledge that at a volume fraction of $\phi = 0.001$, the chromosomes are able to diffuse freely within the large confinement and thus, the system does not reach a steady state of mixing. As a result, a heterogeneous probability distribution of the contact map is observed. We have addressed this in our previous comments and in the paper in the new Fig.S4, as we mentioned above.

**Reviewer 1 said:** *11) In Fig. 4 (a), the* $(\phi, \epsilon)$ *pairs at (0.01, 0.2), (0.001, 0.75) and (0.3, 0.75)* do *have a yellow or orange color, which disagrees with the argument in Line #473.*

**Our response:** We apologize for the mistake in our previous submission. The heatmap we provided was based on mixing exponent $\beta$ calculated from 100 time frames of simulations, but we later used 1000 time frames to recalculate $\beta$ for the updated heatmap. Our argument in the paper was based on the new heatmap. The heatmap includes many colors corresponding to each order of timestep. These colors are sensitive to the value of the mixing exponent ($\beta$). The revised manuscript now contains the new heatmap based on 1000 time frames and is now consistent with the text. We also added to Fig. 4(a) a color-coded scale showing the mixing time.

Even the old heatmap did show yellow and orange colors for the $(\phi, \epsilon)$ pairs at (0.01, 0.2), (0.001, 0.75), and (0.3, 0.75), but the mixing time for these pairs is less than 5 years, which is shorter than the reptation time. Therefore, even the old heatmap does not affect the final state diagram in Fig. 4(b).

To clarify this for readers, we have added a new Fig.S13 with a matrix which shows the value of $\beta$ for each pair of $(\phi, \epsilon)$. This figure also demonstrates that the phase diagram in Fig. 4(b) can be derived based on the value of $\beta$.

**Reviewer 1 said:** *12) In Line #515, how about interaction strength between euchromatic and heterochromatic beads, $\epsilon_{EH}$?*
**Our response:** Euchromatic and heterochromatic beads only interact through excluded volume interactions. Therefore, the Lennard-Jones potential between these beads is truncated at the distance where the attractive and repulsive forces are equal, which occurs at a distance of $r_c = 2^{1/6}\sigma$ and an energy of $\epsilon_{EH} = 1\,\mathrm{k_B T}$.

We added following lines in revised manuscript on line 548.

"Euchromatic and heterochromatic beads only interact through excluded volume interactions."

**Reviewer 1 said:** *13) Please fix the typos: "the number intra-chromosomal" in Line #74; "the extent chromosome mixing" in Line #98; "we also the mixing within a single chain by calculating" in Line #100; "and shown that" in Line #124; "In Section, we" in Line #169; "as functions of $\phi$" in Line #368; "between the mixing order times" in the caption of Fig. 4; the comma in Line #565 can be deleted.*
**Our response:** In the revised manuscript, we have corrected those typos.

# Response to reviewer 2

**Reviewer 2 said:** *In this manuscript, Bajpai & Safran propose to systematically study chromosome intermingling using polymer simulations. In particular, they focus on the role of confinement and self-interaction on the dynamics of chromosome territories mixing.*

*Recently, in the biological community, the interest for functional inter-chromosomal contacts increased (see recent Peter Fraser's work for example). Therefore, a generic and systematic analysis of chromosome intermingling might be of interest. In particular, since the number of modeling works addressing that question are quite rare.*

*However, the manuscript contains several major issues, both on substance and form, that have to be addressed or discussed.*

**Our response:** Thank you for your review of our manuscript. We appreciate your positive feedback on the focus of our work, which aims to systematically study chromosome intermingling using polymer simulations, particularly the role of confinement and self-interaction in the dynamics of chromosome territory mixing. We agree with you that the topic of functional inter-chromosomal contacts is of growing interest in the biological community and hope that our generic and systematic analysis of chromosome intermingling will be of value, given the limited number of modeling works addressing this question.

**Reviewer 2 said:** - *On the form: o The manuscript is very descriptive and contains lots of unclear/unjustified statements (see other remarks below). o It contains also many typos (a careful proofreading is required) and lots of repetitions that can be removed (eg, on the definition of $P_c$ or $\beta$ in lots of results subsection). o The introduction is strangely written: first there is a brief standard-like introduction reviewing the literature (but containing again many repetitions : the abstract is there (top of page 3) or L87-99 Page 3 versus L50-61 Page 2), followed by a kind of extended summary of the results that would be more appropriate in the conclusion/discussion part.*

**Our response:** Thank you for your review and for bringing these issues to our attention. We have carefully proofread the manuscript and eliminated unnecessary repetitions and typos. We have also reorganized the introduction to follow a more standard structure, including moving the extended summary of our results to the conclusion/discussion section.

**Reviewer 2 said:** *On the methods: o Authors may want to say that homologous chromosomes in Drosophila are paired to motivate that they only simulate 4 chromosomes (instead of 4 pairs).*

**Our response:** Thank you for this comment. Starting on line 159 of the revised manuscript, we have added the following sentences.

"In *Drosophila*, the genome is organized into four pairs of chromosomes. However, when simulating *Drosophila* genome we only consider four chromosomes instead of 4 pairs. This is because homologous chromosomes share significant sequence similarity and generally behave similarly.'

**Reviewer 2 said:** *The authors use a very rigid polymer model (Kuhn length of 300 nm,* $L_k/\sigma = 10$*) which is certainly non-biologically relevant in vivo. More recent estimations of chromatin fiber rigidity lead to* $L_k = 100$ *nm (eg, https://doi.org/10.1093/nar/gkz374). May be nice to discuss more the differences/similarities between the simulations done of* $l_p = 5$ *and 1.*

**Our response:** The Kuhn length of chromatin fibers, which is a measure of the persistence length of the polymer, has been reported with several values in in different papers, indicating that the precise value is not yet known. Some papers have reported a value of 300 nm for the Kuhn length of chromatin fibers [6, 17–22]. However, upon reviewing many papers on this topic, we have found that the reported range of Kuhn lengths for chromatin fibers is between 100 and 300 nm. In our simulation, we have used the extreme values of this range and simulated chromatin fibers with Kuhn lengths of 60 nm and 300 nm (persistence lengths of 1 bead and 5 beads).

In Fig. 2 (b) and (d), Fig. 3 (b) and (d), Figs. S1, S7, S10, and S11, we have already compared the results of simulations with persistence lengths of 1 and 5 for the exponent of chromosomal mixing index, contact probabilities, and radius of gyration.

**Reviewer 2 said:** *Usually, FENE potentials are more adapted to simulate topologically constraint polymers. Why choosing a simple harmonic potential?*

**Our response:** The choice of a potential function used in a simulation depends on the specific goals of the simulation and the properties of the system being modeled. In our simulation we studied the scaling, power-law behavior of the chromosome mixing index with time. As is known from polymer physics, scaling laws depend on generic regimes (e.g., in classical polymer physics: dilute, semi-dilute, melt), and the power-law exponents are not sensitive to many of the microscopic details. We found that the power law behavior did not change when using FENE, and that the harmonic potential was more computationally efficient as it had fewer parameters and was faster to evaluate. This was especially important because the simulation ran for a large number of time steps or on a system with 35,000 beads. Indeed, there are several previous works related to chromatin modeling where they used harmonic potential [11, 23, 24].

In response to reviewer question, we did one simulation using FENE potential and calculated the power law behavior of chromosome mixing index with time. We compared the results with the harmonic potential and found that the exponent was very similar.

To address this, a new Fig.S16 has been included in the revised supplementary information, with the following caption

"Comparing the power law of the chromosome mixing index calculated from different

types of potentials: In the harmonic + simple Lennard-Jones (LJ) model, the bonded interaction between chain's beads is described by a harmonic potential, while the non-bonded interaction is described by a truncated LJ potential (for more information on these potentials, see the Materials and Methods section). In the FENE + simple LJ model, the bonded interaction is described by a finite extensible non-linear elastic (FENE) spring, while the non-bonded interaction is described by a truncated LJ potential (for more information on the FENE potential, see the reference [25]). In the FENE + shifted LJ model, the bonded interaction is described by a FENE spring, while the non-bonded interaction is described by a Week-Chandler-Andersen (WCA) potential for excluded volume and a truncated and shifted LJ potential for attractions (for more information on the shifted LJ potential, see the reference [25]). In the figure, we see that the scaling law for the mixing index as a power of the time (obtained from the slope of the log-log plot) is very similar for all these potentials."

**Reviewer 2 said:** *Authors use the same Lennard-Jones potential to describe steric interactions and attractions. Again, this is non-standard and can actually be confusing. Indeed, as epsilon is changed, it modifies both the excluded volume and attraction contributions, in particular as the LJ potential is not shifted (but truncated). It might be better to use a standard Week-Chandler-Andersen potential for excluded volume and a truncated and shifted LJ potential for attractions (see eg, https://doi.org/10.1016/j.celrep.2019.08.045).*

**Our response:** We conducted simulations using a truncated Lennard-Jones potential and compared the power law of the chromosome mixing index with the results from a simple Lennard-Jones potential. We found that the results were similar. (See our comments above about the nature of scaling laws.)

A new Fig.S16 has been included in the revised supplementary information, with the its caption that is copied in our response to the previous comment.

Starting on line 621 of the revised manuscript, we have added the following sentences.

"Also, we used simple LJ and harmonic potentials, which are commonly used in polymer simulations. Scaling laws depend on generic regimes (e.g., in classical polymer physics: dilute, semi-dilute, melt), and the power-law exponents are not sensitive to many of the microscopic details. We have also shown that the power-law behavior did not change when using FENE and shifted LJ potentials (see Fig.S16) [25]."

**Reviewer 2 said:** *The details about the block copolymer simulations (and LAD) are not described.*

**Our response:** In the revised manuscript we have added a new section of the SI, S4

text, titled "Block copolymer model for *Drosophila* genome: Incorporating chromosome size, euchromatin and heterochromatin domains, and the interaction of lamin with LAD and non-LAD regions" where we have added details of our block copolymer model. The text is somewhat lengthy, and we do not copy it here; we request that the referee see the new text in section S4 of the SI.

**Reviewer 2 said:** *For a more realistic time mapping between simulation and real time units, it is better to map the simulated MSD with experimental measurements (see eg https://doi.org/10.1016/j.molcel.2018.09.016) (with Fig.S1 for example).*

**Our response:** Here, the experiment data suggested by the reviewer is of yeast chromosomes. In living yeast cells, experiments on high-throughput chromatin motion tracking have shown that the mean squared displacement (MSD) follows a scaling law for $MSD \sim t^{1/2}$, with a power-law exponent of 1/2 [1,2]. More generally, Ref. [3] shows that the MSD of a polymer is related to how it diffuses in its surroundings which might include other monomers of the same chain, other chains, or a confinement. The general scaling law is: $MSD(t) \sim t^{2\nu/(2\nu+1)}$. When the scaling exponent is $\nu = 1/2$, as is the case for a random walk chain, the MSD obeys $MSD \sim t^{1/2}$. The observation of this pattern in yeast chromosomes suggests that they obey random walk statistics.

However, it is important to note that the MSD of chromosomes can be influenced by their organization and can vary among cells and cell types. In cases where the scaling exponent is $\nu = 1/3$, as is the case for a collapsed, self-attractive chain, a chain in a poor solvent or a chain in small confinement volume, the MSD has a power-law of 0.4. Our study, shown in Fig. S1, demonstrate how the MSD changes with chromatin-chromatin interactions, and Fig. S11 shows how confinement can change the scaling exponent from 0.5 to 0.4.

Starting on line 343 of the revised manuscript, we have added the following sentences. "High-throughput chromatin motion tracking experiments in living yeast cells have revealed that the mean squared displacement (MSD) of chromosomes follows a scaling law of MSD $\sim t^{1/2}$, indicating that they obey random walk statistics [1, 2]. However, the MSD can be influenced by the organization and confinement of chromosomes and can vary among cells and cell types [3]. The scaling law for MSD of a polymer is MSD($t$) $\sim t^{2\nu/(2\nu+1)}$, where $\nu = 1/2$ for a random walk chain and $\nu = 1/3$ for a collapsed, self-attractive chain, a chain in a poor solvent, or a chain in a small confinement volume. Our study (shown in Fig.S1) demonstrates how chromatin-chromatin interactions affect the MSD. In Fig.S11, we calculated the average mean-squared displacement (MSD) of four chains over time for different attraction strengths, $\epsilon$. The range of mixing exponents $\beta$ is consistent with Rouse dynamics for weak interactions and a smaller exponent for stronger interactions [3–5]."

**Reviewer 2 said:** *To calculate the predicted Hi-C data, authors use a threshold distance of* $1.5\sigma = 45$ *nm, while Hi-C data have been associated to threshold distance of*

*the order of 100-200 nm (eg, doi:10.1101/gr.275827.121). Does it affect the main results ?*

**Our response:** If we increase the cutoff to $> 2\sigma$, a space can exist that allows an additional bead to fit in between. Previous theoretical studies have considered the size of a chromatin bead to be approximately 30 nm, similar to our model. One study used a cutoff of $1.5\sigma$, equivalent to 45 nm, for determining contact probability [26]. Another work used $1.8\sigma$ [27] as the cutoff. In our initial simulation tests, we also calculated the contact probability when two beads were separated by $2.5\sigma$, and we observed that this did not change the chromatin mixing index maximal value ($\alpha = n - 1$) in simulations for the case of small confinement volumes.

**Reviewer 2 said:** *It is unclear how the initial configurations are constructed, in particular to force that there is no knot.*

**Our response:** A similar comment was made by reviewer 1. In response, we copy here our response to him/her.

To prepare the separated, unknotted chains in spherical confinement for our simulations, we followed this protocol:

1. We used the moltemplate software and python code *interpolate_coords.py* [14] to generate an initial configuration of a single chain with 8810 beads. This code creates a smooth polymer structure within a cubic box using cubic spline interpolation (see Fig.S2 (a), (b)).

2. To confirm that the generated initial configuration was unknotted, we performed a self-avoiding chain simulation with a persistence length of 1 bead and a simple Lennard-Jones potential with a strength of $\epsilon = 1k_BT$ and a cutoff of $r_c = 2^{1/6}\sigma$. This generated an open chain structure, which is not possible when the initial structure is knotted (see Fig.S2 (c)).

3. We used the packmol software [15] to generate four copies of the chain in a cubic box with a size of $L = 100\sigma$.

4. We then compressed the polymers system to a small spherical confinement radius of $R_c = 20\sigma$ (or $\phi = 0.6$) using indented walls and Lennard-Jones interactions (see Fig.S3). The force exerted by a spherical indenter on each bead is represented by the equation $F(r) = -K_{\text{indent}}(r - R_{indent})^2$, where $K_{\text{indent}}$ is the specified force constant, $r$ is the distance from the bead to the center of the indenter, and $R_{\text{indent}}$ is the radius of the indenter [14]. The inter-chain interactions were repulsive during compression, with a strength of 1 $k_BT$ ($\epsilon_{\text{inter}} = 1k_BT$) and a cutoff distance of $2^{1/6}$ sigma ($r_c = 2^{1/6}\sigma$). The intra-chain interactions were attractive, with a strength of 1 $k_BT$ ($\epsilon_{\text{intra}} = 1k_BT$) and a cutoff distance of $2.5\sigma$ ($r_c = 2.5\sigma$).

We have now added the snapshots in new Figs. S2 and S3.

Starting on line 224 of the revised manuscript, we have added the following sentences.

"For this biological reason and to obtain a lower bound on the mixing time, we used unknotted chromosome chains as the initial condition. Using moltemplate software [14], we created a single chain of 8810 beads and confirmed that it was unknotted (see the details in Fig.S2). We then placed four copies of this chain in a cubic box and compressed them into a small spherical confinement using indented walls [16]. The Lennard-Jones interactions between the intra-chain were attractive, while the inter-chain interactions were repulsive in the creation of the initial structure. This can be seen in Fig.S3, which illustrates the initially separated and condensed 4 chains in a confinement."

**Reviewer 2 said:** *The predicted HiC shown in the figures are very noisy, meaning that authors should compute them with more statistics (ie independent simulated trajectories)*
**Our response:** We agree with reviewer predicted contact map shown in figures are noisy. It may be due to the use of a minimal (number of) parameters in our simulation to allow for conceptual simplicity and because scaling laws are often robust and not sensitive to many of the microscopic details. In our simulations, we always used the same interaction for intra-chromosome and inter-chromosome interactions. Previous studies that simulated genomes used different interactions for intra-chromosome and inter-chromosome interactions. We show in the new are Fig.S15 that even when intra-chromosome and inter-chromosome interactions are the same, a decrease in inter-chromosome interactions can be observed in Hi-C maps due to lamin interaction in the LAD domains of each chromosome.

In one simulation, we decreased inter-chromosome interactions compared to intra-chromosome interactions and were able to generate a contact map that closely resembles Hi-C genome data, where each separated chromosome can be seen in a diagonal pattern (see Fig.S15).

We emphasize that our study does not focus was not on comparing (in detail) the contact map to Hi-C data, but rather on the scaling laws that quantify how the chromosome mixing index changes with time. We included the contact map in our study as a visual representation of the extent of inter-chromosome mixing.

Regarding generating more statistics, it is not feasible without conducting new simulations. Our current simulation generated 1000 frames and we calculated contact maps from the last 500 frames. To generate more frames, we would have to conduct new simulations for 84 pairs of $(\phi, \epsilon)$. Each simulation takes between 3 and 5 days to run on 32 processors, depending on $\epsilon$. This is not feasible for us at this time.

Starting on line 610 of the revised manuscript, we have added the following sentences. "Although our calculated contact maps are noisy, it may be due to the use of a minimal number of parameters in our simulation in order to maintain conceptual simplicity. This is because scaling laws are often robust and not sensitive to many of the microscopic

details. In our simulations, we always used the same interaction for intra-chromosome and inter-chromosome interactions. Previous studies that simulated genomes used different interactions for intra-chromosome and inter-chromosome interactions, even in data-driven models where interactions were taken from Hi-C contact maps to match the same Hi-C contact maps [28–30]. In one simulation, we decreased inter-chromosome interactions compared to intra-chromosome interactions and were able to generate a contact map that more closely resembles Hi-C genome data, where each separated chromosome can be seen in a diagonal pattern (see Fig.S15)."

**Reviewer 2 said:** *Authors observe a non monotonic behaviors for the exponent beta as a function of $\epsilon$ or $\phi$, but give little (and argued) physical explanations for this non monotonicity.)*

**Our response:** We have observed that the mixing exponent ($\beta$) is non-monotonic when the chromosome-chromosome attraction strength ($\epsilon$) is varied in a large confinement volume. When the attraction strength is weak, the chromosomes do not attract each other and each chromosome diffuses independently within the large confinement volume, resulting in little mixing. However, when the attraction strength is greater than or equal to the critical value ($\epsilon \geq \epsilon_c$), the chromosomes mix because the intra-chromosome and inter-chromosome interactions are same. Here, the critical attraction strength, $\epsilon_c$, is the point at which a single chromosome first collapses. For a persistence length of 1 bead, $\epsilon_c = 0.3k_BT$, and for a persistence length of 5 beads, $\epsilon_c = 0.4k_BT$. When the attraction strength is very strong ($\epsilon \geq 1k_BT$), the chromosomes mix little due to a jamming situation. Similarly, when the volume fraction ($\phi$) is varied with a small attraction strength, little mixing occurs in small volume fractions due to diffusion and in large volume fractions, the dynamic of the beads is restricted due to the small confinement.

**Reviewer 2 said:** *Extrapolation subsection: authors estimate the mixing times by imposing $\alpha(t) = \alpha_{max}$. But this $\alpha_{max}$ value has a meaning only in a highly confined system where the equilibrium state is a fully mixed state. For other situations (less confinement for example), the mixing time would be better defined as the typical time to reach the equilibrium alpha value (which is likely to be different and lower than $\alpha_{max}$). Also, in the case where the mixing time is minimal ($\phi = 0.4, \epsilon = 0.3$), it may be good to launch long simulations to check if the extrapolation is correct.)*

**Our response:** In the revised manuscript, we did not calculate the mixing time when the confinement diameter is larger than the polymer radius of gyration, since chromosomes will not mix as each chromosome diffuses in a large confinement region and does not reach equilibrium.

A long simulation is not possible for the mixing time is minimal ($\phi = 0.4$, $\epsilon = 0.3$), because the chromosomes mix on the order of $10^8\tau$, and the simulation was only run for $10^6\tau$. This would take more than 300 days to complete. To address the reviewer's question, we ran a simulation where there are no springs between beads. In that case, the mixing is fast, so we started with separated molecules and reached equilibrium for mixing.

The simulation also showed a power law for the mixing index, and when it reaches its maximal value $\alpha = n - 1$ (3 in our case of 4 chains), it saturates (see Fig.S8).

Starting on line 380 of the revised manuscript, we have added the following sentences.

"In Fig.S8, we have shown that the mixing index in disconnected beads with excluded volume interactions also follows a power law with time but reaches its maximum value more quickly than for beads connected into chains. This suggests that the connectivity leads to slow mixing."

**Reviewer 2 said:** *Fig.5:"we observe that heterochromatic regions do not mix..": well experimentally pericentromeric regions do mix (look at the contacts between them in a Hi-C map, eg, https://doi.org/10.1371/journal.pgen.1008673).*

**Our response:** In this case, we meant that there is no interchromosomal mixing of the heterochromatic regions. The pericentromeric heterochromatin (PCH) domains of each chromosome are in a condensed state with intrachromosomal mixing, but do not mix with PCH domains from other chromosomes."

We have corrected the caption of Fig.5 to say: "We observe that heterochromatic regions of different chains do not mix due to their large self-attraction that maintains intrachromosomal mixing."

**Reviewer 2 said:** *Fig.5c,f+Fig6c-d: visually it is clear that the predicted Hi-C maps poorly described the experimental one (Fig6e) even if the alpha values are similar. Any comment ? (note that to be fair the pericentromeric regions in the experimental Hi-C map should be represented [even if no data can be assigned to them]). It seems also that the predictions for gamma are not really quantitative compared to the Hi-C data in fly.*

**Our response:** The contact map calculated from our simulation (Fig. 6(d)) is noisy, but similar to the experimental contact map (Fig. 6(e)). Both maps show that the euchromatic parts of chromosome 2L and 2R are separated with little mixing, as well as chromosome 3L and 3R, as evidenced by the five red squares on the diagonal of Fig. 6(d) and 6(e). This is interesting because it was achieved using only one interaction parameter in the simulation (attraction strengths between all euchromatic beads were the same in our simulation). The mixing index calculated from the simulation is similar to the experimental one, but it should be noted that the mixing index also depends on the confinement radius (or volume fraction $\phi$). In the Hi-C experiment, the radius of the nucleus is unknown.

Regarding the pericentromeric region, we were unsure of what interaction strength to use between PCH domains, as experimental PCH domain Hi-C data is also unknown. A high interaction strength of $\epsilon_{HH} = 1$ was used in our simulation, but any value $\epsilon \geq 0.5$

could be used as it leads to a smaller radius of gyration due to collapse. This can also change the simulation results and calculated contact map. The simulation results also show that $\gamma \approx 1$ when the chains are little mixed (for $\epsilon = 1$), which is observed in Hi-C data in *Drosophila* [6].

**Reviewer 2 said:** *There are now available data at different time point along the cell cycle or embryogenesis for example (Dekker lab, Blobel lab, Vaquerizas lab). Would be interesting to see the experimental dynamics of alpha extracted from these data.*
**Our response:** In revised manuscript we now compared the $\alpha$ at different time point along the cell cycle, using HiC data.

A new Fig.S14 (showing $\alpha = 0.64$ for the early embryo and $\alpha = 0.3$ for the late embryo) has been included in the revised supplementary information.

Starting on line 605 of the revised manuscript, we have added the following sentences.

"In Fig.S14, we calculated the chromosome mixing index ($\alpha$) using Hi-C data from two different stages of *Drosophila* development. We found that the index was $\alpha = 0.64$ in the early embryo and $\alpha = 0.3$ in the late embryo, which supports previous experimental results indicating that chromosome mixing occurs in the early embryo [29]."

**Reviewer 2 said:** *In the abstract, in the results and in the conclusion, authors state that the beta exponent is related to the MSD diffusion exponent by just observing some correspondences. While it is indeed likely that both are connected, it is unclear if it is just a coincidence or if there is more physical explanations (that are not demonstrated in the present paper) behind this connections.*
**Our response:** In the revised manuscript starting at line 510, we wrote a few lines to support how mixing index is related to RMSD:

"We note that the mixing at the lowest level depends on the motion of individual beads in their environment of other chains and confinement. The correspondence of the chromosome mixing index $\alpha$ and the RMSD time dependence for the motion of a bead shows that the coarse-grained $\alpha$ has dynamics that follows a simple mean-field behavior of the single bead dynamics, but appropriately modified so that the RMSD exponent accounts for the environment of the bead; this depends on the attraction strength and the overall confinement volume (bead volume fraction)."

**Reviewer 2 said:** *Minor comments - Some unclear statements that need to be rephrased or more detailed:*
*o Page 2 L27-32*
*o P7 L268 "ideal limit": unclear.*
*o P7 L283-286*
*o P9 L320-322: and so what ? what is the connection with the rest of the paragraph?*

*What do we learn ?*
*o P9 L341-345: and so what ? what is the connection with the rest of the paragraph? What do we learn ?*
*o P11 L378-381: this statement is true for every* $\epsilon$ *value.*
*o P12 L402-410*
*o P12 L420-421: I do not think there is evidence that a nematic ordering is observed in mitotic chromosome.*

**Our response:** Page 2 L27-32
Old text: However, mixing can occur without reptation by topological entanglement, just by intermingling of the different polymers. Such motion can be characterized by its mean square displacement (MSD) of a polymer segment which increases with the square root of time as in the Rouse model (MSD $\sim \tau^{1/2}$) [31, 32] and with smaller exponents for interacting chains as described below.

Revised text: However, mixing can occur without reptation (that involves topological entanglement), by simple intermingling of the separate chains. This type of motion can be characterized by the mean square displacement (MSD) of a polymer segment, which increases with the square root of time as in the Rouse model (MSD $\sim \tau^{1/2}$) and with smaller exponents for chains that interact with each other [31, 32].

o P7 L268 “ideal limit”: unclear.
Old text: In the Supporting Information (SI) S2 Text, we theoretically show that the ideal limit of the chromosome mixing index is equal to $n-1$, where $n$ is the number of chromosomes within the nucleus, e.g., the maximum value of chromosome mixing index is 3 for a genome with four chromosomes of the same size.

Revised text: In the Supporting Information (SI) S2 Text, we theoretically show that in the ideal limit (when there are no interactions), the chromosome mixing index is equal to $n-1$, where $n$ is the number of chromosomes within the nucleus. For example, the maximum value of the chromosome mixing index for a genome with four chromosomes of the same size is 3.

o P7 L283-286
Old text: We can easily understand this by comparing the radius of gyration and the diameter of confinement. The chromosome mixing index of phantom chains (with radius of gyration $R_g$) reaches its maximal value for relatively small confinement volumes $R_c$, $R_g \gg R_c$ but has a small value when $R_g \ll R_c$.
Revised text: In Fig.S4, the ratio of the confinement size (diameter, $2R_c$) to the radius of gyration ($R_g$) of the phantom chain is plotted for various values of $\phi$. The results

indicate that when $\phi \leq 0.01$, the confinement volume is relatively large, as $2R_c/R_g > 1$. Conversely, when $\phi \geq 0.1$, the confinement volume is relatively small, as $2R_c/R_g < 1$, and the chromosome mixing index for phantom chains reaches its maximum for these values of $\phi$.

P9 L320-322: and so what ? what is the connection with the rest of the paragraph? What do we learn ?

Old text: Additionally, we show in Fig.S2 that disconnected beads in small confinements (large volume fraction of beads) achieve maximal mixing index for non-zero attraction strengths ($\epsilon = 0.25$).

Revised text: In Fig.S8, we have shown that the mixing index in disconnected beads with excluded volume interactions also follows a power law with time but reaches its maximum value more quickly than for beads connected into chains. This suggests that the connectivity leads to slow mixing. Additionally, we have found that mixing is also slow (small mixing exponent) for disconnected beads that are strongly attractive, as seen in Fig.S9, highlighting the effect of attraction on mixing.

o P9 L341-345: and so what ? what is the connection with the rest of the paragraph? What do we learn ?
Old text: To better understand how the attraction strength influences the condensation of a single-chain, we first simulated a single chain without confinement and calculated the radius of gyration as a function of the attraction of the beads (see Fig.S1). In the Fig.S3, we also discuss the analytical virial coefficient [33] for the LJ potential which we compare with the value obtained from the simulations at which the chains collapse due to the attractive interactions between the beads.

Revised text: To better understand how the attraction strength influences the condensation of a single-chain, we first simulated a single chain without confinement and calculated the radius of gyration as a function of the attraction of the beads (see Fig.S1). In the Fig.S6, we also discuss the analytical virial coefficient [33] for the LJ potential which we compare with the value obtained from the simulations at which the chains collapse due to the attractive interactions between the beads. For a given value of $\epsilon$, computation from the simulations of the radius of gyration of the chain indicates the range of attraction strengths for which the chain is open and those where the chain is collapsed.

o P11 L378-381: this statement is true for every $\epsilon$ value.
Our response: Yes.

Old text: At larger attraction strengths ($\epsilon = 1\,\mathrm{k_B T}$), each chain is collapsed but rarely

mixes with the other because the kinetics slow down significantly as the time required to break the bond is $e^{(z\epsilon/k_B T)}$, where $z$ is the number of nearest neighbors (not counting the two nearest neighbors to which each beads is bonded).

Revised text: At larger attraction strengths ($\epsilon = 1\,\mathrm{k_B T}$), each chain collapses but rarely mixes with the other because the kinetics slow down significantly as the time required to break the bond is $e^{(z\epsilon/\mathrm{k_B T})}$, where $z$ is the number of nearest neighbors (not counting the two nearest neighbors to which each bead is bonded). In addition, the concentration of the chain is relatively larger in the collapsed state, which may make the chain kinetics more "jammed" for the condensed states at large values of the attraction. Fig.2 (b) for $\epsilon = 1$ shows two mixing exponents, $\beta_1$ and $\beta_2$, indicating that the beads begin to mix at early times with a mixing exponent of $\beta_1 = 0.14$, while at late times, the mixing exponent is very close to zero ($\beta_2 = 0.04$), indicating a "jammed" situation. In summary, when polymer chains are in a large confinement volume (corresponding to small volume fractions $\phi \leq 0.001$), they are able to mix effectively only if they have moderate attractive interactions ($0.4 \leq \epsilon \leq 0.75$).

o P12 L402-410

Old text: In order to see contact probability behavior across several (four) chromosomes, we calculated contact probability from each chromosome and plotted the average in Fig.2 (d). In right panel of Fig.2 (d), we calculated the scaling exponent ($\gamma$) as a function of attraction strength ($\epsilon$) which shows similar trends like single chromosome in which $\gamma > 1$ for open chains and $\gamma \leq 1$ for collapse chain. Collapsed chain shows an intermediate level mixing for $0 < \gamma < 1$. We have also shown simulation results of mixing of four chains in relatively small confinement (see Fig. S5) for simulation snapshots, mixing index, and contact probability. Also see Fig.S6 and S2 Table for MSD).

Revised text: In order to analyze the contact probability behavior for the four chromosomes, we calculated the contact probability from each chromosome and plotted the average in Fig.2 (d). In the right panel of Fig.2 (d), we calculated the scaling exponent ($\gamma$) as a function of attraction strength ($\epsilon$), which shows similar trends to that of a single chromosome, where $\gamma > 1$ for open chains and $\gamma \leq 1$ for collapsed chains. Collapsed chains show an intermediate level of mixing for $0 < \gamma < 1$. We also show simulation results of the mixing of four chains in relatively small confinement volume (see Fig.S10 for simulation snapshots, mixing index, and contact probability). The results indicate that when confinement volume is small, the chromosome mixing index follows a power law for any attraction strength less than 1 ($\epsilon < 1$). In Fig.S11, we calculated the average mean-squared displacement (MSD) of four chains over time for different attraction strengths, $\epsilon$. The range of mixing exponents $\beta$ is consistent with Rouse dynamics for weak interactions and a smaller exponent for stronger interactions [3–5].

o P12 L420-421: I do not think there is evidence that a nematic ordering is observed in mitotic chromosome.

**Our response:** We agree with reviewer's comment. We deleted the line mitotic chromosome.

Old text: The nematic ordering observed at this high volume fraction is related to the comparable size of the confinement radius and the persistence length of 5 beads for this simulations, which gives a structure similar to mitotic chromosomes.

Revised text: The nematic ordering observed at this high volume fraction is related to the comparable size of the confinement radius and the persistence length of 5 beads for this simulation.

**Reviewer 2 said:** *P7 L251-252: really, no knots in phantom chains? where is the evidence?*

**Our response:** These chains easily mix in relatively small confinement, but completely unmix when confinement size is increased, indicating a lack of knots.

Old text: In our results we see evidence of no knots in two important limiting cases (i) phantom chains (see Fig.1) that can be thought of as being dominated by chromosome cutting enzymes (e.g. Type II topoisomerase [7]). (ii) Strongly attractive chromatin [8, 9] where we show (see Fig.2 and Fig.3) that the steady state structure is that of a fractal globule which again has no knots [10–12].

Revised text: Even though we began simulations with unknotted chains, chain mixing can result in knots forming between chains. Type II topoisomerases enzymes are known for removing DNA knots [7]. In our results, we found that there are no knots in two important limiting cases: (i) phantom chains (see Fig.1) that can be thought of as being dominated by chromosome cutting enzymes (e.g. Type II topoisomerase [7]). These chains easily mix in relatively small confinement volumes, but completely unmix when the confinement volume is increased, indicating a lack of knots. (ii) Strongly attractive chromatin [8, 9] where we show (see Fig.2 and Fig.3) that the steady state structure is that of a fractal globule which again has no knots [10–12]. This can be understood by coloring subchains with different colors, and observing that when these subchains remain separated, the structure is unknotted [13]. Our results in Fig.S1 (a) for the case of $\epsilon = 1$ and persistence lengths of 1 and 5 beads, show that the subchains correspond to different colors are separated.

**Reviewer 2 said:** *VIBGYOR?*

**Our response:** In revised manuscript, we now explain this.

Old text: The heat map displays the time in seven colors according to the VIBGYOR colors pattern for the various pairs of $(\phi, \epsilon)$; this shows which pairs show complete mix and on which time scale.

Revised text: The heat map displays the time in seven colors according to VIBGYOR (Violet, Indigo, Blue, Green, Yellow, Orange, Red) colors pattern for the various pairs of $(\phi, \epsilon)$; this shows which pairs show complete mixing and on which time scale.

**Reviewer 2 said:** *P15 L474-483: biological relevance of such long time-scales?*
*- In Drosophila, an 8 min cell cycle is only observed during early embryogenesis (cycle number ¡ 13), then cell cycles become longer (hours) and potentially cells stop dividing.*
**Our response:** The biological importance of long time-scales in chromosome mixing is demonstrated by the fact that chromosomes can survive for extended periods of time without intermingling.

In revised manuscript we have now written that during early Drosophila embryogenesis, the cell cycle is shorter (8 min).

**Reviewer 2 said:** *P4 L140: regarding gel-like analysis, authors may consider https://doi.org/10.1038/s41467-019-10628-9 or doi:10.1101/gr.275827.121*
**Our response:** We have added the citations regarding gel-like analysis in the revised manuscript.

**Reviewer 2 said:** *P 5, L166-168, authors may cite previous work integrating LAD at a genome-wide scale (eg, https://doi.org/10.1016/j.bpj.2021.11.936)*
**Our response:** We have added the citation to the previously mentioned work integrating LAD at a genome-wide scale in the revised manuscript.

**Reviewer 2 said:** *Fig1b right: bar(alpha) is the time-average of alpha, but once the system reach equilibrium or since the beginning of the simulations?*
**Our response:** In Fig1b right, we calculated the time-averaged mixing index ($\bar{\alpha}$) using the last 300 frames out of a total of 1000 frames.

Old text: We varied the volume fraction $\phi$ of chains (representing the chromosomes) within the nucleus, and calculated the time-averaged chain mixing index ($\overline{\alpha}$) as shown in right panel of Fig.1 (b).

Revised text: We varied the volume fraction, $\phi$, of chains (representing chromosomes) within the nucleus and calculated the time-averaged chain mixing index, ($\overline{\alpha}$), from the last 300 frames out of a total of 1000 frames, as shown in the right panel of Fig.1 (b).

**Reviewer 2 said:** *Fig.1b-1c: please use the same color code*
**Our response:** In the revised manuscript, we have now used the same color for Fig.1b and 1c.

**Reviewer 2 said:** *Fig.1c: I found very suspicious that for such a low density (0.001), the authors do not recover exactly the expected behavior for a phantom chain ($\gamma = 1.5$).*
**Our response:** In the revised manuscript, the newly added Fig.S5 demonstrates that the scaling exponent of the contact probability, $\gamma$, for an completely ($phi = 0$) unconfined phantom chain is indeed $\approx 1.5$. When confinement is introduced even for a volume fraction of 0.001, $\gamma$ decreases to 1.25, and for a small confinement with a volume fraction of 0.1, $\gamma$ decreases further to 1.

**Reviewer 2 said:** *Fig.1f-g: please add vertical and horizontal lines to help visualizing the separation between the different chromosomes.*
**Our response:** We have added vertical and horizontal lines to the revised manuscript to improve visualization of the separation between different chromosomes.

We added following line in Fig.1 of the revised manuscript

"In contact maps (f) and (g), vertical and horizontal colored lines have been added to distinguish the chains, which correspond to the colors of the chromosomes."

**Reviewer 2 said:** *Fig.2b: for eps=1kT, is alpha really a power law ?*
**Our response:** In the revised manuscript, we demonstrate that when the attraction between chromosomes is very strong ($\epsilon = 1k_BT$), there are two regimes of mixing behavior. On short time scales, the chromosomes mix according to a certain exponent. However, at longer times, the high density of molecules leads to a "jamming" situation, causing the mixing exponent to decrease. In this scenario, complete mixing may not occur, and the mixing exponent may not reach its maximum value ($\alpha = n - 1$).

Starting on line 371 of the revised manuscript, we have added the following sentences.

"Fig.2 (b) for $\epsilon = 1$ shows two mixing exponents, $\beta_1$ and $\beta_2$, indicating that the beads begin to mix at early times with a mixing exponent of $\beta_1 = 0.14$, while at late times, the mixing exponent is very close to zero ($\beta_2 = 0.04$), indicating a "jammed" situation."

**Reviewer 2 said:** *Fig.2b & Fig.3b: the evolution of beta (as a function of eps or phi) seems quite noisy (lots of fluctuations), is it due to a lack of statistics ?*
**Our response:** It can be due to lack of statistics. To calculate statistics, we generate 1000 frames from our simulations. However, if we want to generate more frames, we will need to rerun the simulations. We also note that it is possible that there are only a few

universality classes for these scaling exponents. At this time, doing longer simulations is not feasible for us.

**Reviewer 2 said:** *Fig.3b, 3d: seems that there are a discontinuity between the first two black dots of the beta and gamma curves, why ?*

**Our response:** In Fig. 3b and 3d, the values of the mixing exponent $\beta$ and contact probability exponent $\gamma$ for chromosome volume fractions $\phi = 0.001$ and $\phi = 0.01$ are represented by the first and second dots, respectively. There is a discontinuity between these points because for $\phi = 0.001$, the chromosomes are in a relatively large confinement volume (larger than $R_g$) and are unlikely to be mixed as they freely diffuse across a large portion of the confinement. However, for $\phi = 0.01$, the chromosomes are able to mix due to smaller confinement volume, as indicated by the condition $2R_c/R_g < 1$, where $R_c$ is the confinement radius related to $\phi$ and $R_g$ is the radius of gyration of an unconfined random-walk chain of 4M beads (M is the total number of beads in one chain and we took M = 8810). It is worth noting that in the phantom case, both $\phi = 0.001$ and $\phi = 0.01$ are in the diffusive region).

We demonstrate this more fully in the revised manuscript where we introduced a new figure in the supplementary material, (Fig.S4) that illustrates the relationship between the mixing index of chromosomes and the size and confinement of polymers. The figure shows that the mixing index is maximized when the ratio of the diameter of confinement ($2R_c$) to the radius of gyration ($R_g$) of an unconfined chain is less than one ($2R_c/R_g < 1$), demonstrating the influence of the size and confinement of the polymers on the mixing index.

The caption for this new figure reads: "Confinement smaller than the radius of gyration maximizes the chromosome mixing index: (a) In the phantom chain case ($\epsilon = 0$), the ratio of the diameter of confinement ($2R_c$) to the radius of gyration of a random-walk chain ($R_g$) is plotted as a function of the chain volume fraction in the nucleus. Fig. 1 (c) shows that the radius of gyration of a random-walk chain of $M = 8810$ beads is $R_g = 90\sigma$ when the persistence length is 5 beads. The inset of the figure shows the time-average of the chromosome mixing index ($\bar{\alpha}$) as a function of the chain volume fraction ($\phi$) in the nucleus. From both figures, it is clear that the chromosome mixing index reaches its maximum value when $2R_c/R_g < 1$. Thus when $R_g$ is large relative to $2R_c$ the confinement is effective; in the opposite case, the confinement is so large that the chains hardly mix with their diffusion in the large confinement volume greatly impeding mixing, even for phantom chains. (b) In the case of excluded volume chains (where $\epsilon \neq 0$), the confinement has an even larger effect. The radius of gyration for 4 chains of M beads each (4M beads in total, equivalent to the *Drosophila* genome) is taken to be $R_g = 180\sigma$. It is noteworthy that for $\phi = 0.01$, in the phantom chain case (a) $2Rc/Rg > 1$, but in the non-phantom chains case (b) $2Rc/Rg < 1$."

**Reviewer 2 said:** *Does the authors account for the Rabl-like organization of chromosomes in Drosophila?*
**Our response:** In the Rabl-like organization of chromosomes, the centromere of each chromosome is positioned at one nuclear pole, while the telomeres are positioned at the opposite side. Our polymer model did not take into account the effect of telomeres and centromeres, and we did not verify whether our organization was Rabl-like.

**Reviewer 2 said:** *In Drosophila, knock-down of condensin II during interphase leads to more chromosome mixing (https://doi.org/10.1371/journal.pgen.1007393), suggesting that SMC might have a impact on territories formation. May be interesting to discussed that.*
**Our response:** Thank you for the citation. In our revised manuscript, we have included a paragraph discussing knock-down of condensin II during interphase leads to more chromosome mixing.

Starting on line 578 of the revised manuscript, we have added the following sentences.

"Recent studies have revealed the role of SMC (structural maintenance of chromosomes) proteins, such as condensin II, in the formation of chromosome territories [34]. Research has shown that when condensin II is knocked down in *Drosophila*, there is an increase in chromosome mixing, suggesting that these proteins play a role in the spatial organization of chromosomes within the nucleus. Our simulations show that when the mixing exponent ($\beta$) is greater than 0.1 ($\beta > 0.1$), indicating weak attraction between chromosomes in small confinement volume, chromosome mixing is similar to what would be expected without condensin II. However, when $0 < \beta < 0.1$, indicating strong attraction between chromosomes, chromosome mixing is similar to what would be expected with condensin II present. We suggest that on long-time scales, the activity of condensin II may be captured by an effective self-attraction of the beads. Additionally, we found that lamin proteins, which are involved in chromosome organization and play a role in the formation of chromosome territories, can also reduce chromosome mixing."

# Response to reviewer 3

**Reviewer 3 said:** *The paper "Mesoscale, long-time mixing of chromosomes and its connection to polymer dynamics" by Bajpai & Safran presents results of a computational polymer model for chromosome organization. In particular, the authors are interested in the phenomenology of chromosome mixing during interphase and, to this purpose, they study different scenarios: ideal chains (i.e., no interactions between polymers/chromosomes), interacting chains with short-range monomer-monomer attractions, polymer-lamina in-*

*teractions.*

*In order to distinguish how polymer chains mix in these different contexts, the authors introduce a simple classifier: the mixing index $\alpha$, corresponding to the ratio between the inter-chromosomal interactions and the intra-chromosomal interactions. In the intentions of the authors, the time behavior of this exponent monitors how different chromosomes mix over time.*

*The main topic of the paper (chromosome organization) is timely and using generic polymer models to understand it is certainly interesting. Said that, I see no element of novelty in this paper which may justify its publication in Plos Computational Biology, and I try to motivate my decision:*

**Our response:** We respectfully disagree with the reviewer's assessment that our paper lacks novelty. Our study makes significant contributions to the field by systematically investigating the dynamics of chromosome intermingling using polymer simulations. Our approach is novel in several key ways.

Firstly, we introduce a novel mixing index, $\alpha$, which serves as a simple classifier for comparing the mixing dynamics of different scenarios. To our knowledge, this is the first time such a classifier has been used to study chromosome mixing and we believe it will be a useful tool for future studies in this area.

An important new result is that $\alpha$ obeys scaling laws in which it shows a power-law increase with time. The powers (scaling exponents) can vary but are robust in the sense that they are different for different, generic classes of polymer behavior: phantom chains, excluded-volume chains, weakly attractive chains, strongly attractive chains (where reputation behavior correlates with the scaling exponent), and very attractive (or highly condensed) chains (where there is little dynamics due to jamming). Thus, there are several classes of exponents, but the exponents themselves do not vary with the details of the molecular potentials. We believe that the readers of PLOS Computational Biology are interested not only in detailed, many parameter, data-driven models but also conceptual ones with a minimal number of parameters, where the behavior of a complex system (several chains (chromosomes), confinement in the nucleus, attraction to the lamina) can be understood in a step-by-step manner.

Secondly, our work provides new insights into the role of polymer interactions in chromosome mixing, demonstrating that the mixing dynamics can be strongly influenced by whether the short-range attractions are repulsive or moderately or very strongly attractive, as well and polymer-lamina interactions. These results add to our current understanding of chromosome organization and have potential implications for other areas of biology and biophysics. Our previous modeling of the structural organization of confined and interacting polymers as a model of chromatin enabled the understanding of novel experimental data that measured the phase separation of chromatin in *Drosophila* using intact, live

organism imaging, as published with our experimental, biological collaborators in Science Advances and eLife [35, 36].

Finally, we believe that the topic of functional inter-chromosomal contacts is of growing interest in the biological community and our generic and systematic analysis of chromosome intermingling is of value given the limited number of modeling works addressing this question.

We believe that our contribution will be considered as a valuable addition to the field and look forward to acceptance of our paper in Plos Computational Biology.

**Reviewer 3 said:** *1) I have found the paper confusing, in particular I do not understand why the authors employ different models: ideal polymers (i.e., no excluded-volume), polymers with excluded volume in bulk, confined polymers. If the purpose of the authors is to test different models, how does all this connect to chromosomes?*

**Our response:** It is essential for us to understand how different polymer models, such as ideal polymers, polymers with excluded volume in bulk, and confined polymers, impact the chromosome mixing index since we have introduced it. These models have been used in chromatin modeling and have been used in various applications. For example, the phantom chain model has been used to study the kinetics of chromosome condensation in the presence of topoisomerases [37]. Polymers with different attraction strengths have been used to investigate the chromatin organization in senescence, progeria, and growing cells [25]. Confined polymers are widely used in chromatin biology to predict Hi-C maps from simulations [38, 39]. Furthermore, polymers confined within different volumes of the nucleus have been used to predict chromatin organization in live and fixed cells [36].

The main reason for studying the change in $\alpha$ of these model situations is that simulations with a minimal number of parameters can more easily highlight the physical processes that are important. That will allow experimenters who may measure the dynamics of $\alpha$ to understand which processes and basic scenarios are involved as we explain in more detail in our response to the next point.

**Reviewer 3 said:** *2) The authors claim that the mixing index $\alpha$ may be used to quantify chromosome mixing within the cells: I find this argument particularly weak, because - with respect to the simulations presented by the authors - $\alpha$ depends 'a lot' from parameters like polymer density, polymer overlap in solution, etc... Given the great variability across different cells and different organisms, it is hard to believe that $\alpha$ gives any new insight on chromosomes.*

**Our response:** The organization of chromosomes within the nucleus is not fixed and varies based on a number of different factors. It is important to systematically study

the impact of these factors on key parameters, such as the chromosome mixing index. Recent studies have shown that the volume of the nucleus and the density of the HP1 protein, which is known for binding to heterochromatin domains, change during early Drosophila embryogenesis and vary between cell cycles [29, 40]. This suggests that chromatin-chromatin interactions also change with the cell cycle. Therefore, it is important to determine how the chromosome mixing index is affected by altering chromatin-chromatin interactions, the volume of the nucleus and the interactions with the lamina.

It is important to emphasize that the dyanmics of $\alpha$ obey scaling relations, with $\alpha$ varying as a power-law in time. The power-law is characterized by an exponent and this exponent shows only a small number of values sometimes called classes, depending on the polymer environment (interactions, confinement and lamina binding). Within each class, the exponent does *not* vary with the molecular details but is robust. This is a well-known feature of polymer physics and our work shows how this applies to mixing dynamics of different chains, which is an archetype for chromosome mixing.

In our case, these classes include: (i) polymer diffusion with no mixing in large confinement volumes, (ii) mixing characterized by monomer diffusion as described by non-interacting chains for Rouse dynamics in smaller volumes, (iii) mixing characterized by monomer diffusion in the surroundings of other, moderately attractive chains, (iv) much slower mixing for more strongly attractive chains where polymer reptation dynamics explains how the RMS displacement of a bead varies with time as well as our mixing exponent, and (v) the very strongly interacting case where almost no mixing occurs and the system is highly condensed and jammed. Each of these regimes results in a different power-law which is robust and does not vary with molecular details (within each class of system).

This conceptual classification may enable future experiments to measure the mixing exponent dynamics. By comparing the measured value of the scaling exponent with these archetypical cases, the experimenters will understand more about the environmental (interactions, confinement, laminar attraction) factors in their system.

**Reviewer 3 said:** *3) I have found the presentation of the work poorly organized. There are frequent repetitions in the text (for instance, in the Introduction, the sentence starting at line 57 and the sentence starting at line 93 are basically the same), not to mention that there are too many frequent typos as "the the" in the caption of Fig. 3, line 8 which indicate that the text has not been proofread duly.*

**Our response:** We have worked to address these issues in the revised version of the paper by carefully proofreading the text and eliminating repetitions and typos. We believe that the revised version is more clear and better organized, and we hope that it addresses your concerns.

Thank you for your feedback. We appreciate the opportunity to improve the quality of our work, and we hope that the revised version of the paper will be more satisfactory.

**Reviewer 3 said:** *Last but not least, the authors dedicate a whole paragraph to 'Physical units of chromosome mixing models' , in particular they say that their time units corresponds to 60us in 'real time'. This looks ok, but why then - throughout the text - do they seem to forget about it and continue referring to MD time units $\tau$? They should have used 'real time' units everywhere.*

**Our response:**

The numerical value of the time parameter, $\tau$, depends on the radius of the chromatin blobs where coarse-grained DNA resides. There are many ways to determine how much DNA can fit within a given radius of chromatin blob. Thus, if the radius of the chromatin bead changes, $\tau$ also changes. The use of dimensionless quantities in each figure allows for the identification of how a specific quantity changes in relation to another. For example, in our calculation of mixing time, we found that there is a difference of many orders of magnitude in time between the mixing of polymers whose dynamics are diffusional (including interactions and confinement) and those whose that are so condensed that their dynamics is governed by reputation. In addition, we find that there are polymers that are so condensed that they are jammed and hardly mix, with scaling exponents significantly smaller than those predicted from reptation. Additionally, previous studies have also used dimensionless quantities in figures, even though they define physical parameters [3, 11]. The dimensionless parameters allow one to focus on the physical phenomena and its conceptual basis which is the focus of our paper. We agree that in more specific, data-driven studies, real units may be more useful, but such studies almost always involve a large number of parameters.

**Reviewer 3 said:** *In conclusion, it is my opinion that this work does not contain material suitable for publication in Plos CB. In addition, the level of presentation is not adequate for publication and, should the authors decide to resubmit elsewhere, careful proofreading of the text is urged.*

**Our response:** The paper has been thoroughly revised by adding more detail to the text, modifying the main figures, and including many new supplementary figures and text. Please see the questions and responses to the other referees (who did not question the basic philosophy of the paper) to get a sense of the extent of the scientific revisions. We also carefully proofread the paper to minimize typos. We believe that these revisions have significantly improved the quality of our work and will make a meaningful contribution to the field of chromatin research. Your consideration and the time you have taken

to review our manuscript is greatly appreciated. We respectfully request that in your re-evaluation, you consider as appropriate for PLOS Computational Biology, papers that are more conceptual (minimal parameter models), that focus on scaling behavior (which is indeed experimentally measurable), in addition to highly data-driven studies.

## References


[1] Hajjoul H, Mathon J, Ranchon H, Goiffon I, Mozziconacci J, Albert B, et al. High-throughput chromatin motion tracking in living yeast reveals the flexibility of the fiber throughout the genome. Genome research. 2013;23(11):1829–1838.

[2] Buckle A, Brackley CA, Boyle S, Marenduzzo D, Gilbert N. Polymer simulations of heteromorphic chromatin predict the 3D folding of complex genomic loci. Molecular cell. 2018;72(4):786–797.

[3] Liu L, Shi G, Thirumalai D, Hyeon C. Chain organization of human interphase chromosome determines the spatiotemporal dynamics of chromatin loci. PLoS computational biology. 2018;14(12):e1006617.

[4] Ghosh SK, Jost D. How epigenome drives chromatin folding and dynamics, insights from efficient coarse-grained models of chromosomes. PLoS computational biology. 2018;14(5):e1006159.

[5] Tortora MM, Salari H, Jost D. Chromosome dynamics during interphase: a biophysical perspective. Current opinion in genetics & development. 2020;61:37–43.

[6] Halverson JD, Smrek J, Kremer K, Grosberg AY. From a melt of rings to chromosome territories: the role of topological constraints in genome folding. Reports on Progress in Physics. 2014;77(2):022601.

[7] Champoux JJ. DNA topoisomerases: structure, function, and mechanism. Annual review of biochemistry. 2001;70(1):369–413.

[8] Gibson BA, Doolittle LK, Schneider MWG, Jensen LE, Gamarra N, Henry L, et al. Organization of Chromatin by Intrinsic and Regulated Phase Separation. Cell. 2019;179(2):470 – 484.e21. doi:https://doi.org/10.1016/j.cell.2019.08.037.

[9] Bajpai G, Padinhateeri R. Irregular Chromatin: Packing Density, Fiber Width, and Occurrence of Heterogeneous Clusters. Biophysical Journal. 2019;118(1):207–218. doi:10.1016/j.bpj.2019.11.004.

[10] Mirny LA. The fractal globule as a model of chromatin architecture in the cell. Chromosome research. 2011;19(1):37–51.

[11] Naumova N, Imakaev M, Fudenberg G, Zhan Y, Lajoie BR, Mirny LA, et al. Organization of the mitotic chromosome. Science. 2013;342(6161):948–953.

[12] Lieberman-Aiden E, Van Berkum NL, Williams L, Imakaev M, Ragoczy T, Telling A, et al. Comprehensive mapping of long-range interactions reveals folding principles of the human genome. science. 2009;326(5950):289–293.

[13] Imakaev MV, Tchourine KM, Nechaev SK, Mirny LA. Effects of topological constraints on globular polymers. Soft matter. 2015;11(4):665–671.

[14] Jewett AI, Stelter D, Lambert J, Saladi SM, Roscioni OM, Ricci M, et al. Moltemplate: A tool for coarse-grained modeling of complex biological matter and soft condensed matter physics. Journal of molecular biology. 2021;433(11):166841.

[15] Martínez L, Andrade R, Birgin EG, Martínez JM. PACKMOL: A package for building initial configurations for molecular dynamics simulations. Journal of computational chemistry. 2009;30(13):2157–2164.

[16] Plimpton S. Fast Parallel Algorithms for Short-Range Molecular Dynamics. Journal of Computational Physics. 1995;117(1):1 – 19.

[17] Bystricky K, Heun P, Gehlen L, Langowski J, Gasser SM. Long-range compaction and flexibility of interphase chromatin in budding yeast analyzed by high-resolution imaging techniques. Proceedings of the National Academy of Sciences. 2004;101(47):16495–16500.

[18] Rosa A, Becker NB, Everaers R. Looping probabilities in model interphase chromosomes. Biophysical journal. 2010;98(11):2410–2419.

[19] Dekker J, Steensel Bv. Chapter 7 - The Spatial Architecture of Chromosomes. In: Handbook of Systems Biology. San Diego: Academic Press; 2013. p. 137–151.

[20] Rippe K. Making contacts on a nucleic acid polymer. Trends in biochemical sciences. 2001;26(12):733–740.

[21] Florescu AM, Therizols P, Rosa A. Large scale chromosome folding is stable against local changes in chromatin structure. PLoS computational biology. 2016;12(6):e1004987.

[22] Emanuel M, Radja NH, Henriksson A, Schiessel H. The physics behind the larger scale organization of DNA in eukaryotes. Physical biology. 2009;6(2):025008.

[23] Jost D, Carrivain P, Cavalli G, Vaillant C. Modeling epigenome folding: formation and dynamics of topologically associated chromatin domains. Nucleic acids research. 2014;42(15):9553–9561.

[24] Gibcus JH, Samejima K, Goloborodko A, Samejima I, Naumova N, Nuebler J, et al. A pathway for mitotic chromosome formation. Science. 2018;359(6376):eaao6135. doi:10.1126/science.aao6135.

[25] Chiang M, Michieletto D, Brackley CA, Rattanavirotkul N, Mohammed H, Marenduzzo D, et al. Polymer modeling predicts chromosome reorganization in senescence. Cell reports. 2019;28(12):3212–3223.

[26] Huang K, Li Y, Shim AR, Virk RK, Agrawal V, Eshein A, et al. Physical and data structure of 3D genome. Science advances. 2020;6(2):eaay4055.

[27] Michieletto D, Orlandini E, Marenduzzo D. Polymer model with epigenetic recoloring reveals a pathway for the de novo establishment and 3D organization of chromatin domains. Physical Review X. 2016;6(4):041047.

[28] Fujishiro S, Sasai M. Generation of dynamic three-dimensional genome structure through phase separation of chromatin. Proceedings of the National Academy of Sciences. 2022;119(22):e2109838119.

[29] Zenk F, Zhan Y, Kos P, Löser E, Atinbayeva N, Schächtle M, et al. HP1 drives de novo 3D genome reorganization in early Drosophila embryos. Nature. 2021;593(7858):289–293.

[30] Qi Y, Reyes A, Johnstone SE, Aryee MJ, Bernstein BE, Zhang B. Data-driven polymer model for mechanistic exploration of diploid genome organization. Biophysical Journal. 2020;119(9):1905–1916.

[31] De Gennes PG. Reptation of a polymer chain in the presence of fixed obstacles. The journal of chemical physics. 1971;55(2):572–579.

[32] De Gennes PG. Quasi-elastic scattering of neutrons by dilute polymer solutions: I. Free-draining limit. Physics Physique Fizika. 1967;3(1):37.

[33] Hirschefelder JO, Curtiss CF, Bird RB. Molecular theory of gases and liquids. New York. 1954;.

[34] Rosin LF, Nguyen SC, Joyce EF. Condensin II drives large-scale folding and spatial partitioning of interphase chromosomes in Drosophila nuclei. PLoS genetics. 2018;14(7):e1007393.

[35] Amiad-Pavlov D, Lorber D, Bajpai G, Reuveny A, Roncato F, Alon R, et al. Live imaging of chromatin distribution reveals novel principles of nuclear architecture and chromatin compartmentalization. Science Advances. 2021;7(23):eabf6251.

[36] Bajpai G, Pavlov DA, Lorber D, Volk T, Safran S. Mesoscale phase separation of chromatin in the nucleus. Elife. 2021;10:e63976.

[37] Sikorav J, Jannink G. Kinetics of chromosome condensation in the presence of topoisomerases: a phantom chain model. Biophysical journal. 1994;66(3):827–837.

[38] Di Pierro M, Zhang B, Aiden EL, Wolynes PG, Onuchic JN. Transferable model for chromosome architecture. Proceedings of the National Academy of Sciences. 2016;113(43):12168–12173.

[39] Zhang B, Wolynes PG. Topology, structures, and energy landscapes of human chromosomes. Proceedings of the National Academy of Sciences. 2015;112(19):6062–6067.

[40] Götz M, Messina O, Espinola S, Fiche JB, Nollmann M. Multiple parameters shape the 3D chromatin structure of single nuclei at the doc locus in Drosophila. Nature Communications. 2022;13(1):1–14.
