# Supplementary material for: Mesoscale, long-time mixing of chromosomes and its connection to polymer dynamics
Source: PLoS Comput Biol. 2023 May 25;19(5):e1011142. doi: 10.1371/journal.pcbi.1011142 (PMC10246856; doi:10.1371/journal.pcbi.1011142)
Supplement: Attachment — Submitted filename: 2_Referee_Response_Bajpai_etal.pdf [file pcbi.1011142.s032.pdf]

Response to reviewers' comments

Response to reviewer 2

Reviewer 2: *Authors have addressed some of my previous concerns but eluded many and, while the subject of study is potentially interesting, the revised manuscript still does not meet the standards of a comprehensive, complete paper that can be published in PLoS CB.*
Regarding author answers to my previous comments:
Our response: Thank you for reviewing the first revised version of the manuscript. We appreciate your feedback and apologize for not fully addressing some of your questions. In the second revised version, we have conducted new simulations and analyzed Hi-C experimental data to provide answers to the remaining questions. We have also made modifications to the figures in the main text of the manuscript and added new figures in the supplementary text. The revisions have been highlighted in red for your convenience. Thank you for your continued support.

Reviewer 2: *(1) The authors still have not done a proper time mapping. With their estimation, they find* $\tau = 60\,\mu s$. *Looking at FigS11, a MSD of 100 (bead unit)*^{2} *(that would correspond to about* $0.1\,\mu m^2$*) is achieved after about* $10^4\tau \sim 0.6\,s$ *from their estimation. While in yeast but also in other species a MSD of* $0.1\,\mu m^2$ *is obtained at time of the order of* $100\,s$
Our response: Thank you for your comment. Our simulations show a faster MSD compared to the experimentally calculated MSD of chromosomes. The MSD of a molecule is inversely proportional to viscosity (η). In our simulations, we assumed the viscosity of the nucleoplasm to be 1 cP, which is the same as the viscosity of water. However, the apparent viscosity of the nuclear environment varies widely among different experimental reports, ranging from η = 1–3 cP [1] to 7 cP [2]. In modeling chromosome dynamics, a viscosity of 10 cP was assumed [3]. If we use a higher viscosity of 10 cP instead of 1 cP, it could slow down the MSD by a factor of 10.

As mentioned in our revised manuscript, we believe that the fast MSD ($0.1\,\mu m^2$ in $0.6\,s$) of homopolymers in our simulation is due to the absence of other protein assemblies - specifically, the lamina - that interact with chromatin to slow down the MSD. To test this hypothesis, we added a new supplementary figure (FigS11 (d)) to our revised manuscript, in which we calculated the MSD of chains (chromosomes) from our block copolymer model that interact attractively with the nuclear envelope (as a model of the lamina). Our results show that the MSD ($0.1\,\mu m^2$ in $6\,s$) is slower with polymers in the absence of the lamina, which supports previous experimental work that demonstrates loss of lamin A function increases chromatin dynamics in the nuclear interior [4]. In addition, the MSD

can be slowed by attractive interactions of the chromatin to itself mediated by other proteins (such as free Lamin A in the nucleoplasm as suggested by the experiments of Ref. [4]. We believe that adding lamin A to our simulation will further increase attraction strength between chromosome beads. Since we have shown that MSD slows down when we increase chromatin interaction strength (ϵ), this means that including the effects of additional crosslinking proteins (such as free Lamin A in the nucleoplasm) will slow down the MSD further.

Starting on line 570 of the revised manuscript, we have added the following sentences.

"In Fig.S11 (d), we computed the mean-squared displacement (MSD) of chains (chromosomes) using our block copolymer model modified to include the attraction of chromatin to the nuclear envelope (as a model of the lamina). Our findings reveal that the exponent of MSD $\sim \tau^{0.26}$ is smaller compared to homopolymers that do not interact with the nuclear envelope (as a model of the lamina) (see S11 Fig.). This result supports previous experimental research indicating that the loss of lamin A function increases chromatin dynamics in the nuclear interior [4]."

Reviewer 2: *(2) On the fact that Hi-C map are very noisy: I do not see why the use of a minimal model may be the cause of this "noise". The four polymers are strictly identical so their average intra-chromosome contact maps should be similar and the inter-chromosome patterns also. In all the shown Hi-C maps, intra and inter- patterns are very different. It is not acceptable to make solid conclusions with such poor statistics. I do understand that it may take time but this is required (and I do not see why "scaling laws are often robust and not sensitive to the many microscopic details" may change that). Or maybe one possibility is to correctly compute/estimate error bars that accounts for the lack of statistics. For example, in Fig.2 c, visually it seems that the intra-chromosomal contact are stronger for $\epsilon = 0.5$ than for $\epsilon = 1$, while on Fig.2d $P(s)$ for $\epsilon = 1$ is higher than for $\epsilon = 0.4$. How is it possible ? Idem for Fig.3c,d*

Our response: We are certainly aware that data-driven, multiparameter models can reproduce many more features of the data than the types of models we use. However, our purpose is to identify how much of the mixing dynamics can be captured by minimal models of the type presented here. For that reason, our emphasis is on the scaling exponents that characterize the mixing, in particular the exponent γ associated with the contact probability of two beads within the same chain and the exponent β that characterizes the subdiffusive dynamics and hence mixing of the chains.

In our homopolymer simulations, the original color maps designated only whether there was very little mixing, some degree of mixing, or complete mixing with the use of only two colors in the contact map. With regards to Fig. 2c, it visually appears that the intra-chromosomal contacts are stronger for $\epsilon = 0.5$ than for $\epsilon = 1$, while in Fig. 2d, $P(s)$ for $\epsilon = 1$ is higher than for $\epsilon = 0.4$. This difference is due to the fact that in each contact

map, we used only two colors (red and white) that did not quantitatively account for the degree of mixing. In the revised manuscript, we used many colors (via the jet color scheme) and also displayed the color bar ranges in the contact maps for Fig. 1, 2, 3, 5, and 6. From the color bar ranges, it is now clear that with increasing attraction strength, the contact between two specific chromatin loci also increases. That is why $P(s)$ for $\epsilon = 1$ is higher than for $\epsilon = 0.4$ in Fig. 2d.

We have added the following sentence in Fig. 1's caption.

"Note that contact maps were generated by applying the $\log_2$ transformation to the raw counts of the contact matrix and displaying the color bar in $\log_2$ ratios."

Reviewer 2: *(3) On the fact that in the phantom chains,* $\gamma = 1.25$ *for* $\phi = 0.001$. *According to their computation for* $\phi = 0.001$, *the radius of gyration of the chain is lower than the sphere diameter. Thus, the effect of confinement should be limited and thus one would expect an exponent of* -1.5 *almost over the whole range of genomic distance. Even in the globular case (strong confinement), the first part of the* $P(s)$ *curve (short genomic distance) towards the constant exponent (FigS5) should have an exponent of -1.5 (see Mirny, Chromosome research, 2011).*

Our response: Thank you for your feedback. We agree with your assessment that using $\phi = 0.001$ in the phantom chains simulation resulted in a radius of gyration that is lower than the sphere diameter, which limited the effect of confinement. We acknowledge that γ should be 1.5 in this case. Furthermore, we agree with your suggestion that even in the globular case (with strong confinement), the first part of the $P(s)$ curve that obeys a power law, should have an exponent of $\gamma = 1.5$. As a result, we re-evaluated our simulation data, considering a smaller value of the interbead distance. We now find that $\gamma = 1.5$ for all cases. We have included updated Fig.S5 and Fig.1(c) in the revised version to show this.

Starting on line 284 of the revised manuscript, we have rewritten the sentences.

Old text: For $\phi = 0.1$, the scaling exponent (for the large values of s of beads near the confinement surface) is $\gamma = 0$, which indicates that chromosomes form an equilibrium globule [5,6]. However, for $\phi = 0.001$, the contact probability of two beads separated by a contour length s is characterized by an exponent $\gamma = 1.25$, which is roughly equivalent to that of an unconfined phantom chain where the Gaussian statistics (and our simulations) result in a scaling exponent $\gamma = 3/2$ (see S1 Fig. (d)).

Revised text: For $\phi = 0.1$, the contact probability is constant for large values of s, representing beads that are near the confinement surface. For smaller values of s, we obtain a scaling exponent of $\gamma = 1.5$ (see S5 Fig.). This is consistent with an equilibrium globule structure [5, 6]. However, for $\phi = 0.001$, the contact probability of two beads separated by a contour length s does not significantly saturate and is characterized by an exponent $\gamma = 1.5$, which is equal to that of an unconfined phantom chain where the

Gaussian statistics (and our simulations presented in S1 Fig. (d)) result in a scaling exponent of $\gamma = 3/2$ [7].

Reviewer 2: *(4) On the mixing of pericentromeric domains: experimentally, PCH domains do mix while in authors' model they don't. This should be discussed.*
Our response: Thank you for bringing this matter to our attention. Based on the Hi-C contact map of *Drosophila* in reference [8], indeed show that the pericentromeric heterochromatin (PCH) domains of chromosomes are intermingling with one another. Motivated by this, we have made changes to the interaction potential between PCH domains in our simulation. Instead of using an attraction strength of $\epsilon_{HH} = 1$, we have changed it to $\epsilon_{HH} = 0.5$. As a result of this alteration, we have now observed that the pericentromeric heterochromatin domains mix [8] in updated Figs. 5 and 6 of the revised manuscript.

These changes also apply to our other findings on homopolymers mixing. In our study, we have demonstrated that when the attraction interaction strength is $\epsilon = 0.5$ in small volume confinement, each polymer collapses and mixes with one another. However, for very strong attraction strengths, such as $\epsilon = 1$, each polymer collapses but does not mix with the others due to the significant slowdown of the the kinetics for two attracting monomers to unbind.

We have added the revised text on pages 16 and 17.

Old text: The attraction between any two "euchromatic" beads is taken as $\epsilon_{\mathrm{EE}} = 0.25\,\mathrm{k_B T}$ and between any two "heterochromatic" beads is $\epsilon_{\mathrm{HH}} = 1\,\mathrm{k_B T}$. Euchromatic and heterochromatic beads only interact through excluded volume interactions. In the simulation snapshot in Fig.5 (b), euchromatic regions of the different polymers mix, while their heterochromatic regions do not. The contact map in Fig.5 (c), calculated from the block copolymer model shows more than 4 regions along the diagonal (as in Fig.1 (a)) due to the separate intra-chain mixing of the euchromatic and heterochromatic blocks, corresponding to the chromosomes in Fig.5 (a). From these results, it is clear that heterochromatin regions in the genome mix only rarely due to their large self-attraction. This serves to decrease the overall mixing of the chromosomes compared to homopolymers with smaller attraction strengths.

Revised text: The attraction between any two "euchromatic" beads is $\epsilon_{\mathrm{EE}} = 0.25\,\mathrm{k_B T}$, while between any two "heterochromatic" beads, the interaction is larger, with $\epsilon_{\mathrm{HH}} = 0.5\,\mathrm{k_B T}$. In this simulation, euchromatic and heterochromatic beads do not attract and have only excluded volume interactions. In the simulation snapshot shown in Fig.5 (b), euchromatic regions of the different polymers mix less, while their heterochromatic regions mix to a greater extent. The contact map in Fig.5 (c), calculated from the block copolymer model, shows more than four regions along the diagonal (as in Fig.1(a)) due to the separate

intra-chain mixing of the euchromatic and heterochromatic blocks, corresponding to the chromosomes in Fig.5(a). From these results, it is clear that heterochromatin regions in the genome mix largely due to their large self-attraction [8]. This serves to increase the overall mixing of the chromosomes compared to homopolymers with smaller attraction strengths.

Reviewer 2: *(5) On the comparison between predicted and experimental Hi-C maps: claiming that both are comparable is not justified (look at Fig 5 & 6), and this should be discussed in the text more deeply. Claiming that in Hi-C experiment, the radius of the nucleus is unknown is wrong. There certainly exist estimation of the size of the nucleus in late embryos and so authors may estimate a ϕ value.*

Our response: We calculated the chromosome mixing index from confined volume simulations at three different volume fractions ($\phi = 0.1, 0.2, 0.3$). At a volume fraction of $\phi = 0.3$, we found that our calculated chromosome mixing index (α) was similar to the experimental value. It is worth noting that recent experiments on Drosophila muscle nuclei have suggested a chromatin/nucleus volume ratio of 0.31 [9].

Additionally, it should be noted that the contact maps presented in Fig. 5 and Fig. 6 are different because Fig. 5 shows simulation results for a volume fraction of $\phi = 0.1$, while Fig. 6 shows results for a volume fraction of $\phi = 0.3$.

The simulated contact map we obtained displays similar features to the Hi-C contact map, as both maps show five distinct domains (squares) along the diagonal for chromosomes 2L, 2R, 3L, 3R, and X. This outcome was achieved even though we used identical intra/inter chain interaction strengths in our simulation, and we applied the same interaction strength for sub-chains Chr2L and Chr2R.

In light of the referee comments, we have now calculated the Pearson correlation coefficient ($-1 \leq \rho \leq 1$) between the Hi-C and simulated contact maps. This gives us a quantitative measure of how similar the two maps are [10]. A high correlation coefficient indicates a strong similarity between the two maps, while a low correlation coefficient indicates a weak similarity. The Pearson correlation coefficient between the simulated and experimental contact maps was 0.9.

We have added the revised text on page 20.

Old text: Comparing this with our simulation results for the case shown in Fig.6 (a), we find that the simulations predict a chromosome mixing index of $\alpha = 0.34$ (see Fig.6 (d)).

Revised text: We determined the chromosome mixing index (α) using our block copolymer model for three different volume fractions ($\phi = 0.1, 0.2, 0.3$) in the presence of attractive interactions of the chromatin with the nuclear envelope (as a model of the lamina). The values of α obtained were 0.18, 0.24, and 0.34, respectively. Notably, our calculation

of α for $\phi = 0.3$, as shown in Fig.6 (d), was in good agreement with the experimental value presented in Fig.6 (e). It is worth noting that recent experiments on *Drosophila* muscle nuclei have suggested a chromatin/nucleus volume ratio of 0.31 [9]. The simulated contact map we obtained in Fig.6 (d) displays features that are similar to the Hi-C contact map in Fig.6 (e), as both maps show five distinct domains (squares) along the diagonal for chromosomes 2L, 2R, 3L, 3R, and X. This outcome was achieved even though we used identical intra/inter chain interaction strengths in our simulation and applied the same interaction strength for sub-chains Chr2L and Chr2R. We also calculated the Pearson correlation coefficient ($-1 \leq \rho \leq 1$) between the Hi-C and simulated contact maps, giving us a quantitative measure of how similar the two maps are [10]. A high correlation coefficient indicates a strong similarity between the two maps, while a low correlation coefficient indicates a weak similarity. The Pearson correlation coefficient between the simulated and experimental contact maps was 0.9.

Reviewer 2: *(6) On the changes of alpha during embryogenesis: I was suggesting some recent datasets: in Drosophila the one from Vaquerizas is of excellent quality (Cavalli lab also produced some). And it's clear from these data that chromosomes become more and more mixed as embryogenesis progresses (and not the reverse).*
Our response: As suggested by the reviewer, we conducted an analysis of the Hi-C data for *Drosophila* embryogenesis from the Vaquerizas lab, as reported in the study by Hug et al. (2017) [11]. Our analysis focused on calculating the chromosome mixing index for the developmental stages of Drosophila, specifically during nuclear cycle 12 (nc12), 13 (nc13), and 14 (nc14). Our findings indicate that chromosome mixing gradually increased from nc12 to nc14. As a result, we have added a new Fig. S14 to the second revised supplementary information.

We have added the revised text on page 20.

Old text: In S14 Fig., we calculated the chromosome mixing index (α) using Hi-C data from two different stages of *Drosophila* development. We found that the index was $\alpha = 0.64$ in the early embryo and $\alpha = 0.3$ in the late embryo, which supports previous experimental results indicating that chromosome mixing occurs in the early embryo [8].

Revised text: In S14 Fig., we calculated the chromosome mixing index (α) using Hi-C data from measurements during the different stages of *Drosophila* development [11]. We found that chromosome mixing gradually increased from nuclear cycle 12 to nuclear cycle 14.

Reviewer 2: *(7) New issues regarding the added text: - On the form: The paper is still sometimes hard to grasp (and still contains a non-negligible number of typos) (even for a specialist). Non-expert researchers interested in chromosome organization may still*

have hard times to read it. The introduction is still contained an extended summary of the results that would be more appropriate in the conclusion/discussion part (from Page 3 Line73 to Page 4 L157). The revised manuscript still contains many unclear, unjustified or even wrong statement.

Our response: Thank you for providing feedback on the revised manuscript. Our introduction is quite lengthy because we begin by introducing biological terminology for the general reader, followed by a summary of our main quantitative findings. We have taken into account the issues raised by the referee, including proofreading for typos and simplifying the language for non-expert researchers. In addition, we thoroughly reviewed and revised the introduction to ensure that it provides a clear and concise summary of the paper's objectives and findings. Furthermore, we have rectified some erroneous statements that were automatically corrected while responding to referee comments numbered 3, 4, and 6.

Reviewer 2: *(8)As I suggested in my first review, authors added some references to effect of condensin II or for homologous pairing. But did not discuss them (because they highlight some limitations of the study) or introduce them wrongly.*

- *For example, they cite the condensin II observation in the abstract and also elsewhere in the text, while indeed it may suggest that mixing is under control, it is via a mechanism (loop extrusion) that is not integrated in the present paper. By the way, the sentence "we do not include the short-time ....noise" (L45 Page 2) that is supposed to justify to not integrate loop extrusion is incorrect. Short-time dynamics mechanism (like loop extrusion) may have deep, large-scale effect on genome folding and may not be neglected ! This should be clearly (and fairly) discussed in the Discussion.*
- *Regarding homologous pairing, this is not because "homologous chromosomes share significant sequence similarity that they generally behave similarly" (Page 5 L 159) but in drosophila homologous chromosomes are paired at the molecular scale (btw this may also potentially affect the mixing).*

Our response: Thank you for taking the time to review our revised manuscript. We appreciate your feedback and suggestions, which have helped us improve the quality of our work. We have addressed your concerns regarding the references to condensin II and homologous pairing as follows:

We have added the revised text on page 18.

Old text: Recent studies have revealed the role of SMC (structural maintenance of chromosomes) proteins, such as condensin II, in the formation of chromosome territories [12]. Research has shown that when condensin II is knocked down in *Drosophila*, there is an increase in chromosome mixing, suggesting that these proteins play a role in the spatial organization of chromosomes within the nucleus.

Revised text: Recent studies have revealed the role of SMC (structural maintenance of chromosomes) proteins, such as condensin II, in the formation of chromosome territories [12]. Research has shown that SMC proteins are responsible for controlling mixing via a mechanism called loop extrusion [13, 14]. When SMC is knocked down in *Drosophila*, there is an increase in chromosome mixing, suggesting that these proteins play a role in the spatial organization of chromosomes within the nucleus. A recent theoretical study has shed light on how SMC proteins form large loops in DNA [15]. However, the inclusion of such loop motors and their effects is outside the scope of our paper.

We have added the revised text on page 2.

Old text: Because we focus on the mesoscale and long time dynamics, we do not include the short-time dynamics of molecular motors (e.g. cohesin [16]), whose temporal fluctuations are subsumed in the effective short-time noise [17–19].

Revised text: Because we focus on the mesoscale and long-time dynamics, we do not include the short-time dynamics of molecular motors (e.g. cohesin [16]), whose temporal fluctuations are subsumed in the effective short-time noise [17–19]. However, we cannot ignore the potential large-scale impact of short-term dynamic mechanisms, such as loop extrusion, on genome folding [16, 20]. Previous studies have shown that the average size of chromatin loops ranges from 5 to 200 kbps [21]. In our model, we treat 5 kbps of DNA as a single bead; thus, loops of that size are already coarse-grained. However, longer loops may be significant if they are stable, long-lived, or have a larger lifetimes than the timescale (τ) in our model.

We have added the revised text on page 5.

Old text: In *Drosophila*, the genome is organized into four pairs of chromosomes. However, when simulating *Drosophila* genome we only consider four chromosomes instead of 4 pairs. This is because homologous chromosomes share significant sequence similarity and generally behave similarly.

Revised text: In *Drosophila*, the genome is organized into four pairs of chromosomes. However, when simulating *Drosophila* genome, we only consider four chromosomes instead of 4 pairs because homologous chromosomes are paired at the molecular scale.

Reviewer 2: *(9) • Page 2 L20-25: unclear sentence*

• Page 3 L 88: "we note that all these are structural and not dynamical characterization": actually there exist many (theoretical) studies of chromatin dynamics (eg Ref 36, Ref 41, Ref2, Ref 58).

• Page 20 L627-637: unclear

Our response: Page 2 L20-25: unclear sentence

Old text: However, mixing can occur without reptation (that involves topological entanglement), by simple intermingling of the separate chains. This type of motion can be characterized by the mean square displacement (MSD) of a polymer segment, which increases with the square root of time as in the Rouse model (MSD $\sim \tau^{1/2}$) and with smaller exponents for chains that interact with each other [22, 23].

Revised text: However, mixing can occur without the necessity of reptation motion of a chain end through the tube formed by the other chains; some parts of the different chains can occupy a common volume without any topological effect involving the chain ends. This type of motion can be characterized by the mean square displacement (MSD) of a polymer segment, which increases with the square root of time, as in the Rouse model (MSD $\sim \tau^{1/2}$ [22,23]), and with smaller exponents for chains that interact with each other as theoretically discussed in Ref. [24].

Page 3 L 88: "we note that all these are structural and not dynamical characterization": actually there exist many (theoretical) studies of chromatin dynamics (eg Ref 36, Ref 41, Ref2, Ref 58).

Old text: We note that all these are structural and not dynamical characterizations.

Revised text: In this paper, we correlate the dynamics of chain mixing with the structural contact probability.

Page 20 L627-637: unclear

Old text: In our results, we found that there are no knots in two important limiting cases: (i) phantom chains (see Fig.1) that can be thought of as being dominated by chromosome cutting enzymes (e.g. Type II topoisomerase [25]). These chains easily mix in relatively small confinement volumes, but completely unmix when the confinement volume is increased, indicating a lack of knots. (ii) Strongly attractive chromatin [26, 27] where we show (see Fig.2 and Fig.3) that the steady state structure is that of a fractal globule which again has no knots [5, 28, 29]. This can be understood by coloring subchains with different colors, and observing that when these subchains remain separated, the structure is unknotted [7]. Our results in S1 Fig. (a) for the case of $\epsilon = 1$ and persistence lengths of 1 and 5 beads, show that the subchains correspond to different colors are separated.

Revised text: Our simulation results suggest the absence of knots in two important limiting cases: (i) phantom chains (see Fig.1) that may correspond to chromosomes in the presence of cutting enzymes such as Type II topoisomerase [25]. The simulations show that the chains mix relatively quickly when confined, but once the confinement is removed, they remain un-mixed due to entropy and the absence of interactions between chains. This suggests a lack of knots. (ii) Strongly attractive chromatin [26,27], where we

show (see Fig.2) that the structure is characterized by a contact probability that scales with an exponent $\gamma \approx 1$, which is characteristic of a fractal globule. The fractal globule has relatively little mixing of different sections of the chain, again suggesting the absence of knots [7]. Our results in S1 Fig. (a) for the case of $\epsilon = 1$ and persistence lengths of 1 and 5 beads show that the subchains corresponding to different colors are separated.

- Page 16 L510-516: unclear.

Old text: We note that the mixing at the lowest level depends on the motion of individual beads in their environment of other chains and confinement. The correspondence of the chromosome mixing index α and the RMSD time dependence for the motion of a bead shows that the coarse-grained α has dynamics that follows a simple mean-field behavior of the single bead dynamics, but appropriately modified so that the RMSD exponent accounts for the environment of the bead; this depends on the attraction strength and the overall confinement volume (bead volume fraction).

Revised text: While the details of the mixing dynamics are complex, our observation of a power law for the increase in the mixing index suggests that there may be a dominating effect. We identify this effect with the power-law time dependence of the RMSD, represented by $\lambda/2$. We found, as shown in Table S2, that the exponents $\lambda/2$ and α are very similar and change in response to the different polymer-solvent and polymer volume fraction conditions, such as polymers in a good solvent, poor solvent (self-attractions), high concentration (reptation, similar to melts).

Reviewer 2: *(10) I appreciate the new simulations to show that using FENE or truncated & shifted potentials results may be equivalent. But I cannot see reference into the main text to the corresponding Sup Fig.*

Our response: Thank you for your comments. We have now added a reference to the corresponding Supplementary Figure in the Discussion section of the revised manuscript.

"We have also shown that the power-law behavior did not change when using FENE and shifted LJ potentials (see S16 Fig.) [30]."

Reviewer 2: *(11) In all figures showing simulation snapshots, showing the spherical confinement would allow to visualize better the organization within the sphere (+ adding a ruler to give information on distances)*

Our response: Thank you for your feedback. We understand your suggestion to include a visual representation of the spherical confinement in our simulation snapshots. In the

revised manuscript, we have shown the confinement in Fig.1 and Fig.3.

We added following lines in caption of Fig.1 of the revised manuscript
"The snapshot was zoomed out because its actual size was too large to be shown without taking up an excessive amount of space."

However, in Fig.3, for chains with a radius of gyration less than the confinement radius ($Rg < Rc$), we acknowledge that representing both the snapshots and the spherical confinement in the same figure may be challenging. In such cases, if we represent the snapshots, the confinement would be much larger than the polymer size, and if we represent the confinement, the snapshots may not be visible.

We added following lines in caption of Fig.3 of the revised manuscript

"Note that spherical confinement has not been shown in snapshots because it was too large relative to the chain size to be represented."

Reviewer 2: *(12) Page 20 L 614-616: "previous studies that simulated... used different interactions for intra-....": well most hypothesis driven models do not assume different interactions but same energy strengths (including those in Ref 19 &27).*

Our response:

Old text: Previous studies that simulated genomes used different interactions for intra-chromosome and inter-chromosome interactions, even in data-driven models where interactions were taken from Hi-C contact maps to match the same Hi-C contact maps [8, 31, 32].

Revised text: Previous studies that simulated genomes used data-driven models, which utilized Hi-C contact maps to establish contacts between intra-chromosomal and inter-chromosomal regions, with the aim of comparing them to their corresponding simulated contact maps [8, 31, 32].

Reviewer 2: *(13) Page 14 L476-479: "greater than the typical cell cycle..." : indeed but lower than the typical cell cycle in late embryos...*

Our response:

Old text: which correspond to 1 hour in real time (greater than typical cell cycle time in early *Drosophila* embryogenesis which is 8 minutes [33]).

Revised text: which correspond to 1 hour in real time (greater than typical cell cycle time in early *Drosophila* embryogenesis but lower than the typical cell cycle in late embryos [33]).

References

[1] Baum M, Erdel F, Wachsmuth M, Rippe K. Retrieving the intracellular topology from multi-scale protein mobility mapping in living cells. Nature communications. 2014;5(1):1–12.

[2] Hajjoul H, Mathon J, Ranchon H, Goiffon I, Mozziconacci J, Albert B, et al. High-throughput chromatin motion tracking in living yeast reveals the flexibility of the fiber throughout the genome. Genome research. 2013;23(11):1829–1838.

[3] Brackley CA, Johnson J, Kelly S, Cook PR, Marenduzzo D. Simulated binding of transcription factors to active and inactive regions folds human chromosomes into loops, rosettes and topological domains. Nucleic acids research. 2016;44(8):3503–3512.

[4] Bronshtein I, Kepten E, Kanter I, Berezin S, Lindner M, Redwood AB, et al. Loss of lamin A function increases chromatin dynamics in the nuclear interior. Nature communications. 2015;6(1):8044.

[5] Naumova N, Imakaev M, Fudenberg G, Zhan Y, Lajoie BR, Mirny LA, et al. Organization of the mitotic chromosome. Science. 2013;342(6161):948–953.

[6] Gibcus JH, Samejima K, Goloborodko A, Samejima I, Naumova N, Nuebler J, et al. A pathway for mitotic chromosome formation. Science. 2018;359(6376):eaao6135. doi:10.1126/science.aao6135.

[7] Imakaev MV, Tchourine KM, Nechaev SK, Mirny LA. Effects of topological constraints on globular polymers. Soft matter. 2015;11(4):665–671.

[8] Zenk F, Zhan Y, Kos P, Löser E, Atinbayeva N, Schächtle M, et al. HP1 drives de novo 3D genome reorganization in early Drosophila embryos. Nature. 2021;593(7858):289–293.

[9] Amiad-Pavlov D, Lorber D, Bajpai G, Reuveny A, Roncato F, Alon R, et al. Live imaging of chromatin distribution reveals novel principles of nuclear architecture and chromatin compartmentalization. Science Advances. 2021;7(23):eabf6251.

[10] Tjong H, Gong K, Chen L, Alber F. Physical tethering and volume exclusion determine higher-order genome organization in budding yeast. Genome research. 2012;22(7):1295–1305.

[11] Hug CB, Grimaldi AG, Kruse K, Vaquerizas JM. Chromatin architecture emerges during zygotic genome activation independent of transcription. Cell. 2017;169(2):216–228.

[12] Rosin LF, Nguyen SC, Joyce EF. Condensin II drives large-scale folding and spatial partitioning of interphase chromosomes in Drosophila nuclei. PLoS genetics. 2018;14(7):e1007393.

[13] Fudenberg G, Imakaev M, Lu C, Goloborodko A, Abdennur N, Mirny LA. Formation of chromosomal domains by loop extrusion. Cell reports. 2016;15(9):2038–2049.

[14] Goloborodko A, Marko JF, Mirny LA. Chromosome compaction by active loop extrusion. Biophysical journal. 2016;110(10):2162–2168.

[15] Bonato A, Michieletto D. Three-dimensional loop extrusion. Biophysical Journal. 2021;120(24):5544–5552.

[16] Golfier S, Quail T, Kimura H, Brugués J. Cohesin and condensin extrude DNA loops in a cell cycle-dependent manner. Elife. 2020;9:e53885.

[17] Cugliandolo LF. The effective temperature. Journal of Physics A: Mathematical and Theoretical. 2011;44(48):483001.

[18] Rosa A, Everaers R. Structure and dynamics of interphase chromosomes. PLoS computational biology. 2008;4(8):e1000153.

[19] Gov N. Membrane undulations driven by force fluctuations of active proteins. Physical review letters. 2004;93(26):268104.

[20] Gabriele, Michele and Brandão, Hugo B and Grosse-Holz, Simon and Jha, Asmita and Dailey, Gina M and Cattoglio, Claudia and Hsieh, Tsung-Han S and Mirny, Leonid and Zechner, Christoph and Hansen, Anders S. Dynamics of CTCF- and cohesin-mediated chromatin looping revealed by live-cell imaging. Science. 2022;376(6592):496–501.

[21] Jackson D, Dickinson P, Cook P. The size of chromatin loops in HeLa cells. The EMBO Journal. 1990;9(2):567–571.

[22] De Gennes PG. Reptation of a polymer chain in the presence of fixed obstacles. The journal of chemical physics. 1971;55(2):572–579.

[23] De Gennes PG. Quasi-elastic scattering of neutrons by dilute polymer solutions: I. Free-draining limit. Physics Physique Fizika. 1967;3(1):37.

[24] Liu L, Shi G, Thirumalai D, Hyeon C. Chain organization of human interphase chromosome determines the spatiotemporal dynamics of chromatin loci. PLoS computational biology. 2018;14(12):e1006617.

[25] Champoux JJ. DNA topoisomerases: structure, function, and mechanism. Annual review of biochemistry. 2001;70(1):369–413.

[26] Gibson BA, Doolittle LK, Schneider MWG, Jensen LE, Gamarra N, Henry L, et al. Organization of Chromatin by Intrinsic and Regulated Phase Separation. Cell. 2019;179(2):470 – 484.e21. doi:https://doi.org/10.1016/j.cell.2019.08.037.

[27] Bajpai G, Padinhateeri R. Irregular Chromatin: Packing Density, Fiber Width, and Occurrence of Heterogeneous Clusters. Biophysical Journal. 2019;118(1):207–218. doi:10.1016/j.bpj.2019.11.004.

[28] Mirny LA. The fractal globule as a model of chromatin architecture in the cell. Chromosome research. 2011;19(1):37–51.

[29] Lieberman-Aiden E, Van Berkum NL, Williams L, Imakaev M, Ragoczy T, Telling A, et al. Comprehensive mapping of long-range interactions reveals folding principles of the human genome. science. 2009;326(5950):289–293.

[30] Chiang M, Michieletto D, Brackley CA, Rattanavirotkul N, Mohammed H, Marenduzzo D, et al. Polymer modeling predicts chromosome reorganization in senescence. Cell reports. 2019;28(12):3212–3223.

[31] Fujishiro S, Sasai M. Generation of dynamic three-dimensional genome structure through phase separation of chromatin. Proceedings of the National Academy of Sciences. 2022;119(22):e2109838119.

[32] Qi Y, Reyes A, Johnstone SE, Aryee MJ, Bernstein BE, Zhang B. Data-driven polymer model for mechanistic exploration of diploid genome organization. Biophysical Journal. 2020;119(9):1905–1916.

[33] Shermoen AW, O'Farrell PH. Progression of the cell cycle through mitosis leads to abortion of nascent transcripts. Cell. 1991;67(2):303–310.
